# SARS-CoV-2 infectivity can be modulated through bacterial grooming of the glycocalyx

**DOI:** 10.1128/mbio.04015-24

**Published:** 2025-02-25

**Authors:** Cameron Martino, Benjamin P. Kellman, Daniel R. Sandoval, Thomas Mandel Clausen, Robert Cooper, Alhosna Benjdia, Feryel Soualmia, Alex E. Clark, Aaron F. Garretson, Clarisse A. Marotz, Se Jin Song, Stephen Wandro, Livia S. Zaramela, Rodolfo A. Salido, Qiyun Zhu, Erick Armingol, Yoshiki Vázquez-Baeza, Daniel McDonald, James T. Sorrentino, Bryn Taylor, Pedro Belda-Ferre, Promi Das, Farhana Ali, Chenguang Liang, Yujie Zhang, Luca Schifanella, Alice Covizzi, Alessia Lai, Agostino Riva, Christopher Basting, Courtney Ann Broedlow, Aki S. Havulinna, Pekka Jousilahti, Mehrbod Estaki, Tomasz Kosciolek, Rayus Kuplicki, Teresa A. Victor, Martin P. Paulus, Kristen E. Savage, Jennifer L. Benbow, Emma S. Spielfogel, Cheryl A. M. Anderson, Maria Elena Martinez, James V. Lacey, Shi Huang, Niina Haiminen, Laxmi Parida, Ho-Cheol Kim, Jack A. Gilbert, Daniel A. Sweeney, Sarah M. Allard, Austin D. Swafford, Susan Cheng, Michael Inouye, Teemu Niiranen, Mohit Jain, Veikko Salomaa, Karsten Zengler, Nichole R. Klatt, Jeff Hasty, Olivier Berteau, Aaron F. Carlin, Jeffrey D. Esko, Nathan E. Lewis, Rob Knight

**Affiliations:** 1 Department of Pediatrics, University of California San Diego School of Medicine, La Jolla, California, USA; 2 Bioinformatics and Systems Biology Program, University of California San Diego, La Jolla, California, USA; 3 Center for Microbiome Innovation, University of California San Diego, La Jolla, California, USA; 4 Department of Cellular and Molecular Medicine, University of California San Diego, La Jolla, California, USA; 5 Copenhagen Center for Glycomics, Department of Molecular and Cellular Medicine, Faculty of Health and Medical Sciences, University of Copenhagen, Copenhagen, Denmark; 6 Department of Bioengineering, University of California San Diego, La Jolla, California, USA; 7 Université Paris-Saclay, INRAE, AgroParisTech, Micalis Institute, ChemSyBio, 78350, Jouy-en-Josas, France; 8 Sorbonne Université, Faculty of Sciences and Engineering, IBPS, UMR 8263 CNRS-SU, ERL INSERM U1345, Development, Adaptation and Ageing, F-75252 Paris, France; 9 Department of Medicine, University of California San Diego, La Jolla, California, USA; 10 Department of Biochemistry, Ribeirão Preto Medical School, University of São Paulo, Ribeirão Preto, São Paulo, Brazil; 11 School of Life Sciences, Arizona State University, Tempe, Arizona, USA; 12 Jacobs School of Engineering, University of California San Diego, La Jolla, California, USA; 13 Biomedical Sciences Graduate Program, University of California San Diego, La Jolla, California, USA; 14 Scripps Institution of Oceanography, University of California San Diego, La Jolla, California, USA; 15 Merck & Co., Inc., Rahway, NJ, 07065, USA; 16 Department of Biological & Medical Informatics, University of California San Francisco, School of Pharmacy, San Francisco, California, USA; 17 Department of Surgery, Division of Surgical Outcomes and Precision Medicine Research, Medical School, University of Minnesota, Minneapolis, Minnesota, USA; 18 National Institutes of Health, National Cancer Institute, Center for Cancer Research, Animal Models and Retroviral Vaccine Section, Bethesda, Maryland, USA; 19 Department of Infectious diseases, Luigi Sacco Hospital, Milan and Department of Biomedical and Clinical Sciences (DIBIC), University of Milan, Milan, Italy; 20 Department of Public Health and Welfare, Finnish Institute for Health and Welfare, Helsinki and Turku, Finland; 21 Institute for Molecular Medicine Finland, FIMM - HiLIFE, Helsinki, Finland; 22 Sano Centre for Computational Medicine, Krakow, Poland; 23 Laureate Institute for Brain Research, Tulsa, Oklahoma, USA; 24 Division of Health Analytics, Department of Computational and Quantitative Medicine, City of Hope, Duarte, California, USA; 25 UC Health Data Warehouse, University of California Irvine, Irvine, California, USA; 26 Herbert Wertheim School of Public Health and Human Longevity Science, University of California San Diego, La Jolla, California, USA; 27 Faculty of Dentistry, The University of Hong Kong, Hong Kong, China; 28 IBM T. J. Watson Research Center, Yorktown Heights, New York, USA; 29 AI and Cognitive Software, IBM Research-Almaden, San Jose, California, USA; 30 Division of Pulmonary, Critical Care and Sleep Medicine, Department of Medicine, University of California San Diego, La Jolla, California, USA; 31 International Biomedical Research Alliance, Bethesda, Maryland, USA; 32 Division of Cardiology, Brigham and Women’s Hospital, Boston, Massachusetts, USA; 33 Cedars-Sinai Medical Center, Los Angeles, California, USA; 34 Health Data Research UK Cambridge, Wellcome Genome Campus and University of Cambridge, Cambridge, United Kingdom; 35 Cambridge Baker Systems Genomics Initiative, Baker Heart and Diabetes Institute 104284 , Melbourne, Australia; 36 Cambridge Baker Systems Genomics Initiative, Department of Public Health and Primary Care, University of Cambridge, Cambridge, United Kingdom; 37 Division of Medicine, Turku University Hospital and University of Turku, Turku, Finland; 38 Department of Pharmacology, University of California, San Diego, La Jolla, California, USA; 39 Molecular Biology Section, Division of Biological Science, University of California San Diego, La Jolla, California, USA; 40 Glycobiology Research and Training Center, University of California San Diego, La Jolla, California, USA; 41 Center for Molecular Medicine, Complex Carbohydrate Research Center, and Dept of Biochemistry and Molecular Biology, University of Georgia, Athens, Georgia, USA; 42 Department of Biotechnology and Biomedicine, Technical University of Denmark, Lyngby, Denmark; 43 Department of Computer Science and Engineering, University of California San Diego, La Jolla, California, USA; University of Washington, Seattle, Washington, USA

**Keywords:** SARS-CoV-2, Covid, human microbiome, aging, Heparan Sulfate

## Abstract

The gastrointestinal (GI) tract is a site of replication of severe acute respiratory syndrome coronavirus 2 (SARS-CoV-2) and GI symptoms are often reported by patients. SARS-CoV-2 cell entry depends upon heparan sulfate (HS) proteoglycans, which commensal bacteria that bathe the human mucosa are known to modify. To explore human gut HS-modifying bacterial abundances and how their presence may impact SARS-CoV-2 infection, we developed a task-based analysis of proteoglycan degradation on large-scale shotgun metagenomic data. We observed that gut bacteria with high predicted catabolic capacity for HS differ by age and sex, factors associated with coronavirus disease 2019 (COVID-19) severity, and directly by disease severity during/after infection, but do not vary between subjects with COVID-19 comorbidities or by diet. Gut commensal bacterial HS-modifying enzymes reduce spike protein binding and infection of authentic SARS-CoV-2, suggesting that bacterial grooming of the GI mucosa may impact viral susceptibility.

**IMPORTANCE:**

Severe acute respiratory syndrome coronavirus 2 (SARS-CoV-2), the virus responsible for coronavirus disease 2019, can infect the gastrointestinal (GI) tract, and individuals who exhibit GI symptoms often have more severe disease. The GI tract’s glycocalyx, a component of the mucosa covering the large intestine, plays a key role in viral entry by binding SARS-CoV-2’s spike protein via heparan sulfate (HS). Here, using metabolic task analysis of multiple large microbiome sequencing data sets of the human gut microbiome, we identify a key commensal human intestinal bacteria capable of grooming glycocalyx HS and modulating SARS-CoV-2 infectivity *in vitro*. Moreover, we engineered the common probiotic *Escherichia coli* Nissle 1917 (EcN) to effectively block SARS-CoV-2 binding and infection of human cell cultures. Understanding these microbial interactions could lead to better risk assessments and novel therapies targeting viral entry mechanisms.

## INTRODUCTION

Although the severe acute respiratory syndrome coronavirus 2 (SARS-CoV-2) is responsible for respiratory symptoms associated with coronavirus disease 2019 (COVID-19), the gastrointestinal (GI) epithelium has also been shown to be directly infected (1). GI symptoms are reported in 20.3% of COVID-19 cases (2, 3), GI symptoms frequently appear early in infection before other symptoms develop (4, 5), and individuals with COVID-19 who exhibit GI symptoms often have more severe disease (6). Furthermore, there is evidence that enteric tissue acts as a replicative reservoir for SARS-CoV-2 that can prolong and increase the infectious burden on the host (7–14). Therefore, uncovering the mechanisms and predisposing factors governing potential GI infection by SARS-CoV-2, may lead to better risk stratification and possibly lead to improved therapeutic interventions for COVID-19.

Cell entry and infection by SARS-CoV-2 is dependent upon spike (S) protein binding to both heparan sulfate (HS) and angiotensin-converting enzyme 2 (ACE2) (15, 16), which are present in the GI tract (17). HS is a highly negatively charged component of the glycocalyx, a dense forest of glycans and glycoconjugates that coats all living cells (18). This dense surface of exposed glycocalyx is often the first point of contact for many viruses, including SARS-CoV-2 (19). Nearly all human epithelial surfaces are also home to microbes. The abundance and composition of these microbes, termed the human microbiome, have a close relationship with human health and disease (20–22).

**Fig 1:**
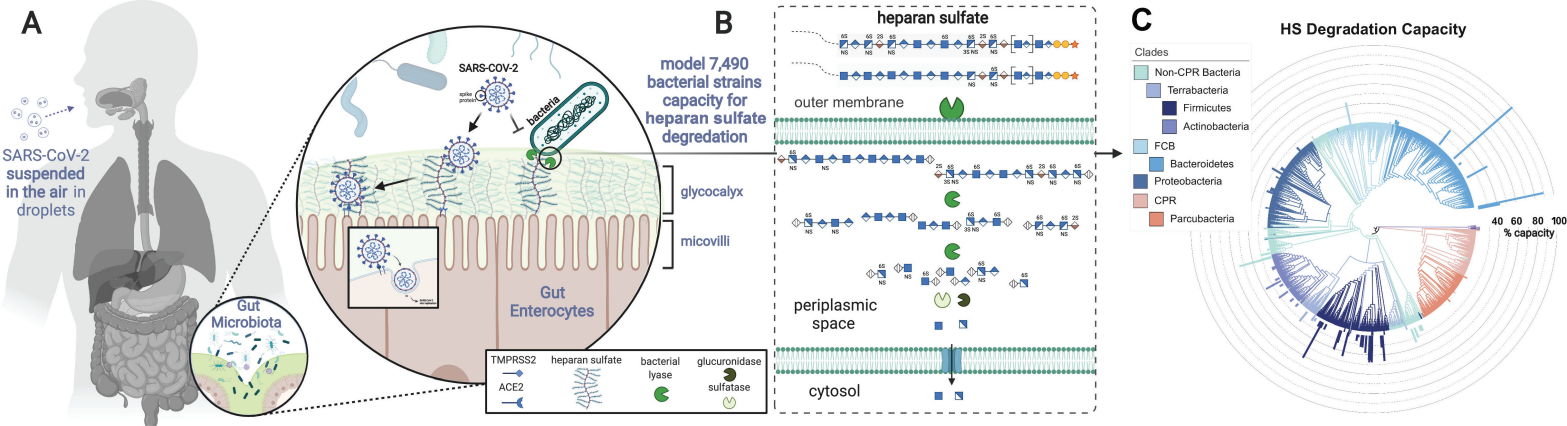
Metabolic task analysis of bacterial species capacity for heparan sulfate (HS) modification across the phylogenetic tree of life. SARS-CoV-2 relies on HS, a major component of the human gut mucosa, for host cell adhesion and entry. Human gut bacteria can alter mucosal surfaces potentially disrupting SARS-CoV-2 cell entry (**A**). Here, we examine the HS degrading genes in 7,490 bacterial strains measured in the FINRISK 2002 data set and quantify the HS catabolic capacity of each strain based on the set of tasks defined in degradation (**B**). Bacterial tree of life colored by superphylum groups and phyla containing predicted HS-modifying species. The bar chart represents the predicted capacity for HS modification of each species colored by phyla (**C**). Created in BioRender. Martino, C. (2025) https://BioRender.com/x09k421.

The glycocalyx component of the mucosa covering the large intestine consists of two layers. The inner layer consists of tightly packed glycosaminoglycans (GAGs), many of which are O-glycosylated, directly anchored to epithelial cells and the outer layer which is a loose assembly of movable GAGs (23). Several host bacteria can produce enzymes that modify specific classes of GAGs, including HS (24) (Fig. 1A). Such glycan-metabolizing genes are often co-localized and co-regulated in polysaccharide utilization loci (PUL) (25). PULs have been annotated in human gut (e.g., *Bacteroides thetaiotaomicron*) (26–28), as well as in fresh water and soil isolates (e.g., *Pedobacter heparinus*) (29). It has been observed that removal of cell-surface HS via heparin lyase (HSase) purified from *P. heparinus* effectively eliminates SARS-CoV-2 virus infection and S protein binding (15). Consequently, we hypothesized that those bacteria that encode the ability to break down mucosa, such as *B. thetaiotaomicron* (26, 30, 31), may modulate entry of SARS-CoV-2 into cells of the GI tract.

## RESULTS

To investigate this hypothesis, we sought to identify host bacteria able to modify host HS, and thereby potentially limiting SARS-CoV-2 viral entry, using a metabolic task-based analysis approach (32) to estimate the catabolic capacity of specific glycan types in each strain across microbial communities (see Materials and Methods). First, we curated a metabolic task describing HS modification and catabolism in the microbiome (Fig. 1B). We then quantified the total counts aligned to each glycosylation-associated gene in each microbe in the FINRISK 2002 data set, a fecal shotgun metagenomic data set spanning over 6,000 samples from participants ranging from 25 to 74 years old with a balanced sex ratio (55% female) (33, 34). Specific inclusion criteria for HS modification genes and HS catabolic measures are described in Materials and Methods. Of the 7,490 species identified in the FINRISK 2002 data set, 463 species of bacteria, which we refer to as HS-modifying bacteria, contained the complete HS catabolic task (completeness = 1) task with high HS catabolic capacity (capacity >90th percentile) (Fig. 1C). For example, three *Bacteroides* species, *B. xylanisolvens*, *B. thetaiotaomicron*, and *B. vulgatus*, were predicted to have extremely high capacity (99th percentile) for HS catabolism consistent with prior reports (26–28).

**Fig 2:**
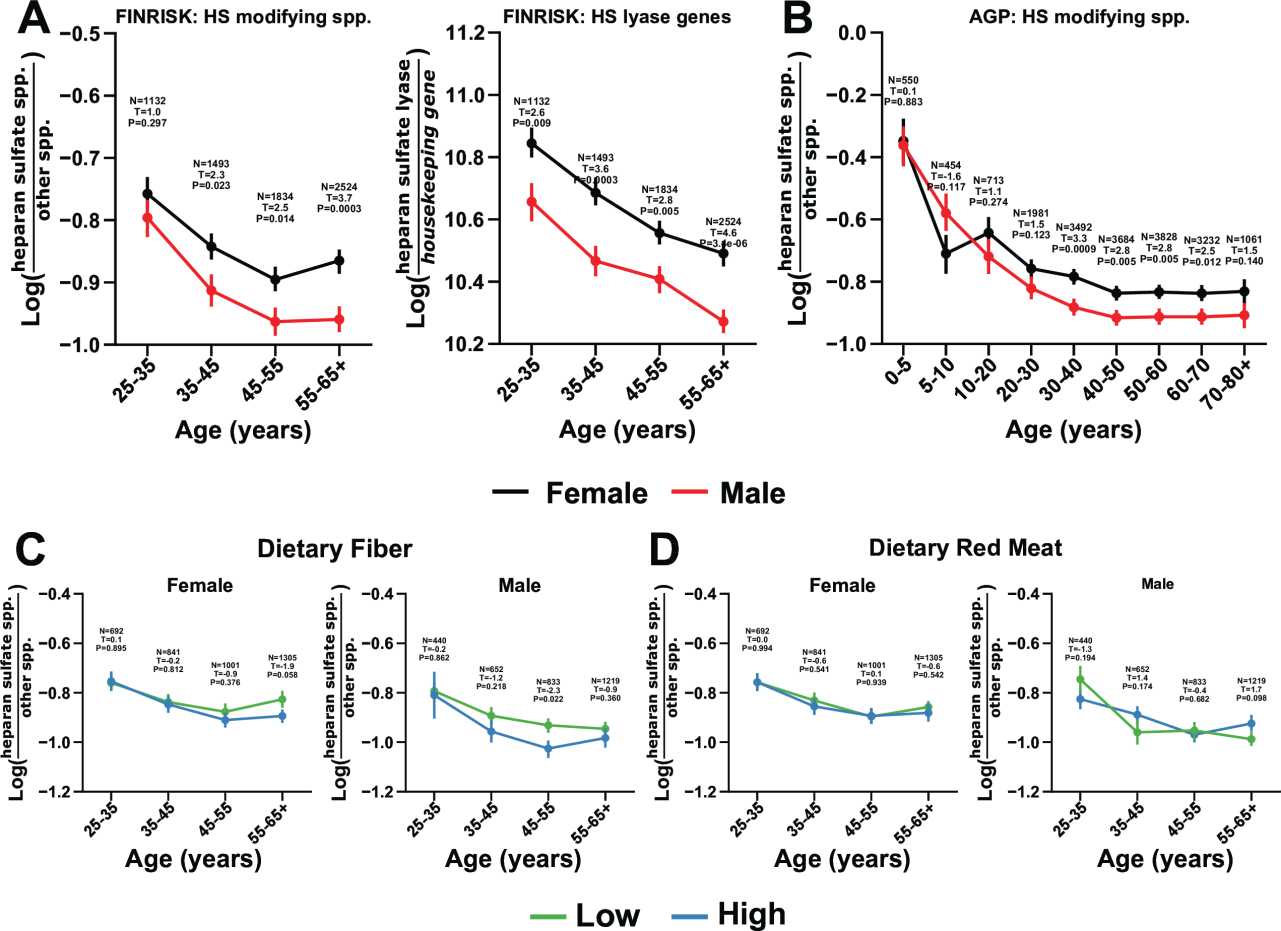
HS-modifying bacteria are inversely enriched according to host age, sex, but not diet. The log-ratio of predicted HS-modifying species relative to those with no predicted capacity for HS degradation (*y*-axis) in the FINRISK 2002 data set compared by host age (*x*-axis). The log-ratio of HS lyase genes relative to a set of housekeeping genes (*y*-axis) in the FINRISK 2002 fecal data compared by host age (*x*-axis) (**A**). The log-ratio of predicted HS-modifying species relative to those with no predicted capacity for HS degradation (*y*-axis) in the AGP fecal data set, compared over host age (*x*-axis) (**B**). Log-ratios are colored by participant sex (women, black; men, red). Log-ratio of predicted HS-modifying species relative to those with no predicted capacity (*y*-axis) in the FINRISK 2002 data set compared by dietary fiber and red meat intake above (high; blue) or below (low; green) the median consumption across ages (*x*-axis) and between sex (panel columns) (C and D). All log-ratio plots across age were annotated by the number of subjects at that time point. Error bars represent the standard error of the mean. Presented *P* values and test statistics are from unpaired two-tailed *t* tests evaluated on each host age group between host sex.

To determine if natural abundances of HS-modifying bacteria are associated with known risk factors and comorbidities of SARS-CoV-2 infection, we compared the relationship of age, sex, diabetes, smoking, body mass index (BMI), cancer, asthma, liver fibrosis or cirrhosis, cardiovascular disease, and autoimmune diseases (35, 36) to the log-ratio of bacteria predicted to modify HS versus all other bacteria (N-strains numerator = 687, denominator = 5,987) in the FINRISK 2002 study. Through an ordinary least squares (OLS) model of HS versus all other bacteria, significant changes by age (*t*-statistic = −2.84, *P* value = 0.005) and sex (*t*-statistic = −5.28, *P* value = 1.3 × 10^−7^) but no other COVID-19 comorbidities were observed (Fig. 2A; Table S1). This finding was validated in an independent data set by matching the Web of Life (WoL) microbial genomes (37) to 16S rRNA gene amplicon sequence variants (ASVs) (see Materials and Methods) from over 20,000 samples in the American Gut Project (AGP), a citizen-science data set with participants ranging in age from 1 to 80 years (20). A similar age- and sex-dependent decrease in HS-modifying bacteria were observed (N-strains numerator = 244, denominator = 1,605) (Fig. 2B). Red meat and diet-derived accessible carbohydrates from dietary fiber are known to generally alter the abundance of mucin glycan degrading bacteria (38, 39). As quantified in the National FINRISK Study 2002 (FINRISK 2002) survey at the time of sample collection, after accounting for age and sex, there was not an overall significant difference in bacteria specifically encoded to degrade HS vs. all other bacteria except in men aged 45–55 by dietary fiber (Fig. 2C and D; Table S1). The alpha diversity across age and sex exhibited previously observed trends (40) (Fig. S1A and B), but HS-modifying bacteria had only a weak negative correlation with alpha diversity (Table S2).

To determine if there was a relationship between the frequency of HS-modifying bacteria and COVID-19 we used a previously published 16S fecal microbiome hospital data set of patients with COVID-19 (41). A subject’s disease severity was determined based on their degree of respiratory support at the time of study participation; moderate disease was deemed for those requiring oxygen without invasive ventilation, and severe disease was for those requiring mechanical ventilation. HS-modifying bacteria were significantly decreased in patients with severe COVID-19 compared to those with moderate COVID-19 (*t*-statistic = −2.3, *P* value  =  0.03) or uninfected healthcare workers (*t-*statistic = −3.9, *P* value  =  0.001) (Fig. S2A). Analysis of a data set of moderate and severe patients with COVID-19 showed that HS-modifying genes relative to housekeeping genes again was significantly decreased in patients with severe COVID-19 (*t*-statistic = 2.9, *P* value  =  0.014) (Fig. S2B). To show that abundance of HS-modifying bacteria is an independent risk factor for COVID-19 infection, we divided the FINRISK participants into those with and without comorbidities, and within each group compared the prior samples of subjects who had and had not later been diagnosed with COVID-19. Among the participants who were infected with SARS-CoV2, only those individuals without comorbidities had significantly diminished ratios of HS-modifying bacteria (Welch’s *t*-statistic = 5.85, *P* value  =  0.027) (Fig. S3A). No significant difference was observed in either HS lyase alone or alpha diversity in those with or without comorbidities (Fig. S3B and C). We then performed shotgun metagenomic sequencing on 416 additional subjects collected after infection through the AGP Microsetta Initiative (20) in collaboration with the California Teachers Study (CTS) and Tulsa 1000 study. This reiterated a significant difference in HS-modifying genes relative to housekeeping genes in those who self-reported having COVID-19 and did not self-report any comorbidities (*t*-statistic = 2.3, *P* value  =  0.0224) (Fig. S2D). Taken together, these results show that those with severe COVID-19 have decreased HS-modifying bacteria compared to those with moderate or no disease before, during, and after infection in an age- and sex-dependent manner.

**Fig 3:**
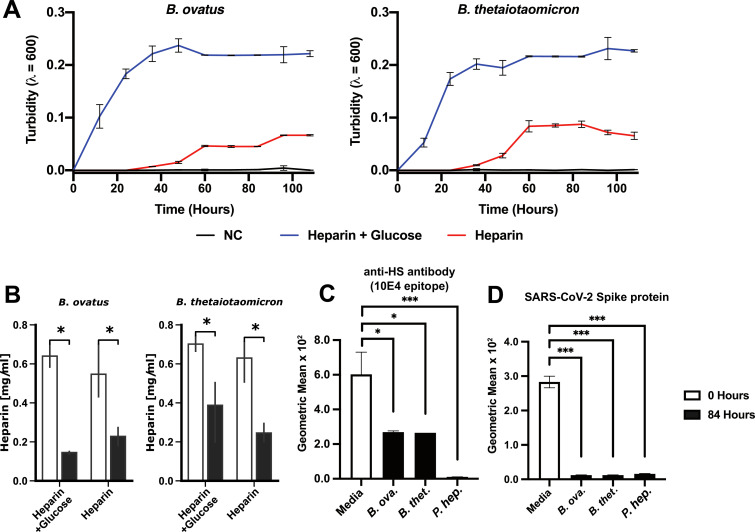
Commensal human gut bacteria block SARS-CoV-2 spike protein binding, through heparan sulfate degradation, in axenic culture supernatant. Growth of *Bacteroides ovatus* and *Bacteroides thetaiotaomicron* (**A**) measured by optical density (*y*-axis) across time from inoculation (*x*-axis) in minimal media (black; negative control NC), minimal media with 22 mM glucose and 1.4 mg/mL heparin (blue), or minimal media with 1.4 mg/mL heparin alone (red). Comparison of heparin concentration (*y*-axis; mg/mL) before inoculation (white; 0 h) and at stationary phase (gray; 84 h) for *B. ovatus* and *B. thetaiotaomicron* (**B**). Geometric mean of flow cytometry data (*y*-axis) of cultured human A549 bronchial epithelial cells stained with the anti-HS antibody 10E4 or incubated with biotinylated SARS-CoV-2 spike protein (**D**). Cells were incubated with culture media (Media), cell-free supernatant of *B. ovatus* (*B. ova*), or *B. thetaiotaomicron* (*B. thet*.) or purified heparin lyase from *Pedobacter heparinus* (*F. hep*.). Presented *P* values are from unpaired *t*-test statistics compared to the untreated control (*P ≤ 0.05, **P ≤ 0.01, ***P ≤ 0.001).

To explore the hypothesis that human gut bacteria modulate HS presentation and therefore SARS-CoV-2 cell entry and infection, *in vitro* experiments were conducted to test the predicted catabolic capacity of these human microbiome species to degrade HS. Axenic cultures of *B. ovatus* and *B. thetaiotaomicron*, highly prevalent human gut bacterial isolates (42) found in 80% of AGP and 99% of FINRISK 2002 participants, were grown on a minimal medium in the presence of heparin (1.4 mg/mL) with and without glucose (22 mM). Both species were able to grow with heparin as the sole carbon source (Fig. 3A). Furthermore, all cultures were verified to catabolize heparin by comparing the concentration of heparin in the medium before and after growth to stationary phase (Fig. 3B). Cell-surface HS on H1299, a lung derived human epithelial cell line, was reduced by 60% upon exposure to cell-free supernatants from mid-log phase cultures of *B. ovatus* or *B. thetaiotaomicron* compared to 100% reduction by purified heparin lyase from *P. heparinus* (*F. hep*. HSase; IBEX pharmaceuticals), as measured by binding of the anti-HS monoclonal antibody 10E4 epitope (Fig. 3C). To determine the effect of bacterial HS modification on SARS-CoV-2 binding, H1299 cells were treated with the supernatant of *Bacteroides* cultures or purified *F. hep*. HSase. These were subsequently incubated with biotinylated trimeric SARS-CoV-2 S protein and assessed for cell-surface binding by flow cytometry. Cells treated with *Bacteroides* culture supernatant exhibited a 20- to 30-fold reduction in SARS-CoV-2 S protein binding compared to untreated H1299 cells (*B. ovatus t*-statistic = 31.25, *P* value  =  7.019 × 10^−5^; *B. thetaiotaomicron t*-statistic = 30.99, *P* value  =  7.023 × 10^−5^), similar to the reduction observed by pre-treatment with purified *F. hep*. HSase (15) (*t*-statistic  =  23.89, *P* value  =  7.59 × 10^−5^) (Fig. 3D).

To identify the human commensal microbial HS-modifying enzymes capable of reducing SARS-CoV-2 S binding, purified GAG-specific sulfatases and HS lyases were tested for their capacity to modulate HS presentation and S protein binding. A total of six human host-resident bacterial exo-active sulfatases from *B. thetaiotaomicron* with different substrate specificities or predicted sulfatases: Δ4,5 hexuronate-2-*O*-sulfatase (BT1596), N-acetylglucosamine-6-sulfatase (BT4656), putative mucin-desulfating sulfatase (BT3177), putative glucosamine N-sulfatase (BT4655), putative iduronate 2-sulfatase (BT0756), and a putative undefined GAG sulfatase (BT1624), were purified after expression in an *Escherichia coli* strain containing a sulfatase maturating system (28) (Fig. S4A). A549 human bronchial epithelial cells were treated with each sulfatase, incubated with S protein, and then assessed for cell-surface binding of S protein by flow cytometry. None of the individual sulfatase treatments significantly decreased S protein binding (Online supplementary file 1) despite the importance of sulfatases for GAG’s degradation by *B. thetaiotaomicron* (28).

**Fig 4:**
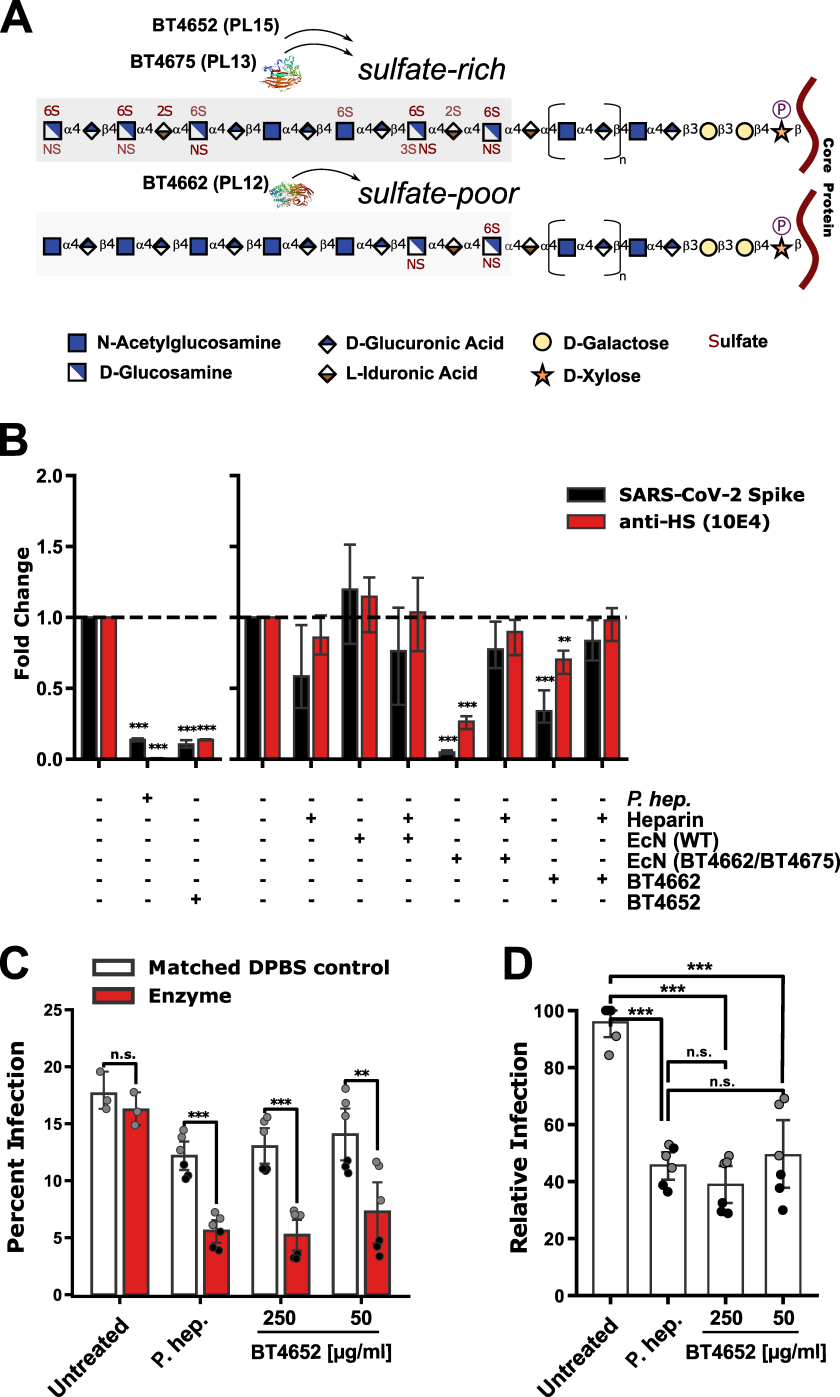
Purified enzymes derived from human commensal gut bacteria block SARS-CoV-2 spike protein binding and viral infection through heparan sulfate modification. Schematic diagram of activity and putative specificity on typical highly and lowly sulfated HS. The bacterial species sourced for enzymes with X-ray crystallographic structures (**A**). Fold change of the geometric mean of flow cytometry data across two experiments relative to untreated control (*y*-axis) of cultured human A549 cells stained with the HS antibody 10E4 (red) or incubated with biotinylated SARS-CoV-2 spike protein (black) compared with and without 100 µg/mL heparin as a competitive substrate between purified *Pedobacter heparinus* (*F. hep*.) heparin lyase (Hsase), purified *B. thetaiotaomicron* heparin Lyase BT4652 (polysaccharide Lyase family 15; PL15), *Escherichia coli* strain Nissle 1917 (EcN) wild-type (WT) culture supernatant, EcN encoding *B. thetaiotaomicron* HS targeting genes BT4662 (polysaccharide Lyase family 12; PL12) and BT4675 (polysaccharide Lyase family 13; PL13) (**B**). SARS-CoV-2 infection of Vero cells performed in the absence and presence of *P. heparinus* Hsase, or with different concentrations of *B. thetaiotaomicron* purified BT4662 heparin lyse. The graph shows the percent infection from a composite of two separate experiments, each performed in triplicate (**C**). The same data but with the experimental data normalized to the mock infection for each respective experiment (**D**). Presented *P* values are from unpaired *t*-test statistics w.r.t. no treatment (*P ≤ 0.05, **P ≤ 0.01, ***P ≤ 0.001).

Next, human commensal bacterial HS lyases were tested for the capacity to reduce SARS-CoV-2 S protein binding. Specifically, three lyases sourced from *B. thetaiotaomicron*: BT4662 (polysaccharide Lyase family 12; PL12) a depolymerizing cell surface lyase that targets sulfate-poor HS, BT4652 (polysaccharide Lyase family 15; PL15), and BT4675 (polysaccharide Lyase family 13; PL13) capable of degrading sulfate-rich HS (Fig. 4A). Enzymes BT4662 and BT4675 were co-expressed in the generally recognised as safe (GRAS) and widely used probiotic *E. coli* strain Nissle 1917 (EcN) with unique purification tags (Fig. S4C). The human cell line A549 was treated with culture supernatant from both the EcN wild type (WT), engineered EcN (BT4662-BT4675), purified lyase BT4652, and a positive control of purified *P. heparinus* HSase (IBEX pharmaceuticals). As demonstrated previously, the positive control, *P. heparinus* HSase eliminated both host-cell HS as detected by 10E4 staining (*t*-statistic = −1,394.6, *P* value = 1.58 × 10^−12^) and binding of recombinant SARS-CoV-2 S protein (*t*-statistic = −95.26, *P* value  =  7.28 × 10^−8^). EcN (WT) culture supernatant produced no significant difference in host cell-surface expression of HS or SARS-CoV-2 S protein binding. On the other hand, EcN (BT4662-BT4675) culture supernatant significantly decreased host-cell surface HS (*t*-statistic = −22.71, *P* value = 2.23 × 10^−5^) and significantly reduced SARS-CoV-2 S binding (*t*-statistic = −59.83, *P* value = 4.67 × 10^−7^). Similarly, purified BT4662 or BT4652 ablated SARS-CoV-2 S binding (BT4662, *t*-statistic = −80.71, *P* value = 1.41 × 10^−7^; BT4652, *t*-statistic = −123.28, *P* value = 6.09 × 10^−13^). Finally, both HS degradation and SARS-CoV-2 S protein binding were prevented by including 100 µg/mL heparin as a competitive substrate in the pre-incubation medium for all *B. thetaiotaomicron* lyases (Fig. 4B; Fig. S5).

To validate the impact of binding on viral infection, we further tested authentic SARS-CoV-2 virus infection using strain USA-WA1/2020. Vero E6 cells were monitored by staining of the cells with antibodies against the SARS-CoV-2 nucleocapsid (N) protein. Cells pretreated for 1 h were infected with virus for 30 min ,followed by the application of methylcellulose overlay to restrict the spread of infection. The impact of infection (MOI of 0.5) was assayed 20 h post-infection (p.i.). Treatment with bacterial heparin lyase in this system has no effect on ACE2 expression (15). Relative infection was calculated by normalizing percent infection values obtained in the absence of any treatment. The positive control treatment of *P. heparinus* HSase significantly reduced percent infection (*t*-statistic = −7.2, *P* value = 2.9 × 10^−5^) and relative infection (*t*-statistic = −12.8, *P* value = 7.69 × 10^−7^) by ~2-fold on average. The treatment of purified BT4652 alone, on average, significantly reduced percent infection (50 µg/mL, *t*-statistic = −3.4, *P* value = 6.66 × 10^−3^; 250 µg/mL, *t*-statistic = −6.2, *P* value  =  1.01 × 10^−4^) and relative infection (50 µg/mL, *t*-statistic = −6.61, *P* value = 2.96 × 10^−4^; 250 µg/mL, *t*-statistic = −12.01, *P* value = 1.44 × 10^−6^) by ~2-fold and ~2.5-fold at a concentration of 50 and 250 µg/ml, respectively (Fig. 4C and D). Both low and high concentrations of purified BT4652 reduced infection to the same extent as *P. heparinus* HSase (Fig. 4C and D). Taken together, these findings demonstrate the potential of commensal human gut bacteria to reduce HS presentation, SARS-CoV-2 S protein binding, and infection in the absence of high quantities of competitive substrate.

## DISCUSSION

In summary, our metabolic task analysis of the human gut microbiome identified a key commensal human intestinal bacteria capable of grooming glycocalyx HS and modulating SARS-CoV-2 infectivity *in vitro*. We observe decreases in predicted HS-modifying bacteria in human gut microbiomes across age and sex in two large survey data sets, in those populations who later contracted SARS-CoV-2, and in severe versus moderate COVID-19 patients in the absence of comorbidities. These data may help to explain age-dependent health differences in the presentation of GI symptoms, infection, and severity of COVID-19. Moreover, the abundance of HS-encoded bacteria is not observed to be consistently enriched in higher fiber diets when accounting for age and sex. Typically, PUL-encoding intestinal bacteria catabolize the outer layer of the mucosa in combination with dietary fiber, leaving the inner layer free of bacteria. In case of decreased dietary fiber, the GI bacteria shift to primarily catabolize host mucosa, including those GAGs found on the inner layer, leaving the mucosa significantly reduced in thickness (31, 39, 43, 44). Our experimental results also demonstrate that the addition of a preferential substrate could reduce the impact of heparin lyase. Therefore, increased fiber diets should be encouraged due to a larger systemic positive impact on COVID-19 severity (45) and overall health (39, 46, 47). However, more work is needed to determine how diet-induced mucosal thickness alteration and HS modification, together impact viral entry and infection across age and gender. Similarly, the microbiome of patients with COVID-19 is also disrupted, with receiving broad-spectrum antibiotics for extended periods of time (41). This further highlights the need for more research to better understand the microbiomes impact, and the impact of the microbiome’s interaction with the glycocalyx, as a risk factor during infection. Although not the focus of this work, similar mechanisms may be active in the respiratory system, which has a strikingly different, but equally important, microbial community and functional repertoire from the gut microbiome (22, 29, 48–50). For example, the human oral microbiome from the AGP data set demonstrated an age-dependent change in HS-modifying bacteria, similar to the gut microbiome samples (Fig. S6). Unfortunately, there are no comparably large oral, nasal, or respiratory microbiome data sets that could be used to explore the contribution of other risk factors. Additionally, there is growing evidence to support the idea of a gut-respiratory axis, where gut microbiota influences the respiratory microbiota/pathology and vice versa (49–51). Altering HS in the gut or in the respiratory tract may thus directly lead to respiratory cell entry and infection blocking of SARS-CoV-2. Further, altering HS in the gut or respiratory tract could play a role in other diseases, including infectious diseases. Therefore, this study opens the possibility of studying similar impacts across various sites of the body to predict, diagnose, and even improve treatment strategies.

These results provide a proof-of-concept for applying metabolic task analysis to quantify the catabolic capacities of microbial communities, particularly in glycomics. Through the use of a synthetic biology approach, we validated our metabolic task analysis and observations in large-scale omics survey data *in vitro*. We observed that the common probiotic EcN can be engineered to effectively block SARS-CoV-2 binding and infection of human cell cultures. Future work will be needed to validate these findings *in vivo*. The systems-level approaches and synthetic biology-driven validations used here can be used to explore the trans-kingdom interplay between resident microbes and host-viral infection.

## MATERIALS AND METHODS

### HS modification capability: completeness and capacity

Many approaches exist to infer or quantify the activities of metabolic gene sets, including topological pathway analyses and mechanistic models (52). However, the use of these methods in the analysis of microbiome omics data can be difficult given the large numbers of microbial species, each with their own set of genes, which require expert curation to define their functions and their collective impact on a species’ metabolic capabilities (53, 54). Accounting for glycan metabolism is particularly important since microbiomes are shaped by their ability to metabolize the host and environmental glycome (55, 56). Metabolic task analysis can rapidly estimate the metabolic capabilities of a cell (32). In this, a metabolic task is defined as the genes associated with the reactions necessary to convert a metabolite into other products. Leveraging this concept in glycan metabolism, the HS modification gene set was treated as a metabolic task, that is, a group of reactions necessary to transition between metabolites (32) used to describe a complete pathway of HS modification. Pathways from the Kyoto Encyclopedia of Genes and Genomes (KEGG, ec00531) (57), in combination with literature annotation of these pathways for specific bacteria (27, 58, 59), were used to identify reactions (enzyme commission [EC] numbers) associated with the modification task (Table S3). Finally, we used CAZy, dbCAN, and CUPP (51, 60–62) to map EC numbers to microbial genes associated with glycan modification (Table S3).

We created two metrics to describe the HS-catabolic capabilities of microbes. For each microbe in the FINRISK2002 data set, we estimated pathway “Completeness,” a binary indication of the presence or absence of all genes in the task, and pathway “Capacity,” a continuous indication of the magnitude of flux that could travel through the task. The abundance of genes associated with glycan catabolism was quantified by the total number of reads (total count) mapped between the HS-modification gene set and FINRISK 2002 sample shotgun data through Bowtie2. A pseudocount was added to each count and log-transformed to stabilize the variance.

HS-catabolic completeness for each organism, *i*, with gene, *g*, is an indication that all reactions in a task are represented in an organism. Specifically, for each reaction (EC) in the HS task, we sum the log-counts aligned to glycosylation-related genes within an organism associated with that EC. If the sum of log counts aligned to an EC exceeds a threshold, *t*, the EC was marked as active in that organism. The threshold, *t*, was set at the fifth percentile of log-counts aligned to glycosylation-associated genes in the FINRISK 2002 database, 4.522 log-counts. If all ECs in the HS task were active in an organism, the HS task is considered complete.


$$O_{i}=\forall_{EC\in Task}t<\sum_{g}^{EC}\sum_{g}^{EC}log\left(read_{g}\right)$$

To quantify the HS-catabolic capacity of each organism, *i*, with genes, *g*, we analyzed the expression of all glycosylation-related genes within the catabolic task. Adapted from metabolic task analysis (32, 63, 64), the catabolic capacity of a microbe is a context-sensitive measure that accommodates rate-limiting or low-abundance enzymes and can therefore accommodate microbial gene transience. Activation of the glycan degrading metabolic task (32), was calculated as the minimum EC activation; the maximum activation of genes performing the EC reaction within an organism. Log of total counts was used to stabilize the variance. The capacity is the minimum EC activation in a pathway where EC activation is the maximum gene activity score for each EC within a microbe. The log of total counts aligned to each gene in the FINRISK 2002 data set was used for the gene activity score:


$$A_{i}=min_{EC\in Task}max_{g\in EC}log\left(reads_{g}\right)$$

### Sequencing data

Patient recruitment, sample collection, storage, and molecular processing are described in detail previously (33, 34). Demultiplexed shallow shotgun metagenomic sequences were quality filtered and adapter trimmed using Atropos (65), and human filtered using Bowtie2 (66). For taxonomic assignment, reads were aligned to the WoL database (37) of 10,575 bacterial and archaeal genomes using Shogun (67) in the Bowtie2 alignment mode. For functional assignment of glycosylation-associated genes, the FINRISK 2002 data were aligned to the HS degradation gene set of glycosylation-associated genes using Bowtie2. Subjects who contracted COVID-19 subsequent to sampling were retrospectively collected in September 2020 (*N* = 21).

The prospective observational CTS includes *N* = 133,477 female participants who have been followed continuously since 1995–1996 (www.calteachersstudy.org). In early 2020, a subset of CTS participants who (i) lived in Los Angeles, Orange, or San Diego counties and (ii) had previously donated biospecimens were invited to participate in the Microsetta Initiative and AGP. All participants provided written informed consent for the data and biospecimens they contributed.

### Abundance and expression of HS-modifying bacteria by log-ratios

Differential abundance or expression of HS-modifying bacteria was determined by the log-ratio of mapped reads for each sample of those bacteria with predicted capacity for HS modification relative to those without predicted capacity. Functional gene abundance or expression was determined with a log-ratio of the total mapped reads to that gene relative to the sum of counts of total mapped reads from a set of housekeeping genes in each sample. The housekeeping gene set is comprised of all bacterial nucleotide sequences for the genes atpD, dnaJ, gyrA, gyrB, infB, pheS, proC, rpoA ,rpoB, and rpoD obtained from RefSeq (68). Significance between groups of log-ratios was determined with an unpaired *t* test through SciPy (69).

### Mapping of WoL to AGP using 16S ASVs

To reconcile the evolutionary relationships among 16S rRNA gene sequencing ASVs and shotgun metagenomic data, we mapped the ASVs to the WoL (37) reference phylogeny of bacterial and archaeal genomes. First, 16S rRNA genes were annotated from each of the 10,575 genomes included in the phylogeny using RNAmmer 1.2 (70), using domain-specific models (bacteria and archaea, respectively). Second, filtered 150 bp length 16S V4 AGP (20), ASVs (*n* = 15,486) were aligned to the WoL 16S rRNA genes using BLASTn 2.7.1+ (71), with an *e*-value threshold of 1e−5 and up to 100 target sequences per query. Top hits with identical bit scores of each query were retained and subjected to the taxonomic classification. At each designated rank, taxonomic assignments of all top hits were recorded. For feature table generation, the hits were counted and normalized by the total number of hits. As an example, assuming one ASV aligned equally well to five reference full-length 16S sequences, and they belong to genus A (two sequences) and genus B (three sequences), then the two genera were counted as 2/5 (A) and 3/5 (B), respectively. Per-query counts were summed across each AGP sample and rounded to integers.

### Cultivation of *Bacteroides* strains

*B. thetaiotaomicron* and *Bacteroides ovatus* were cultured in 50 mL anaerobic brain heart infusion (BHI) medium with an overnight incubation at 37°C in an anaerobic serum bottle. The next day, 1 mL of cells from the BHI culture was washed in anaerobic phosphate-buffered saline (PBS) and were passed into 15 mL of minimal medium containing no carbon sources or electron donors other than glucose (22 mM final) and/or heparin (1 mg/mL final). Growth on the minimal medium was measured by optical density at 600 nm every 12 h. Aliquots of 3 mL of culture were taken before inoculation, immediately after inoculation, at mid-log phase (24 h), and in stationary phase (45 h). Heparin degradation in culture was measured through a Blyscan glycosaminoglycan assay (Biocolor Ltd., Carrickfergus, Northern Ireland) using 100 µL of time 0- and 45-h culture media. All cultivation and HS-modification experiments were conducted in triplicate.

To verify strain taxonomy a 1 mL aliquot of each culture was extracted using PowerFecal DNA Isolation Kit (MoBio cat. 12830). Extracted DNA was quantified via Qubit dsDNA HS Assay (Thermo Fisher Scientific), and 5 ng of input DNA was used in a 1:10 miniaturized Kapa HyperPlus protocol (72). The pooled library was sequenced as a paired-end 150-cycle run on an Illumina NovaSeq at the UCSD IGM Genomics Center. The resulting sequences were adapter trimmed using Trimmomatic v0.39 (73) and human read filtered with Bowtie2 (74). Paired-end reads were merged using Flash v1.2.11 (75). Each axenic sample was assembled through SPAdes (76) and verified through average nucleotide identity (77) of greater than 99% between the assembled genome and the putative type-strain genome obtained from NCBI (68).

### SARS-CoV-2 spike protein production

Recombinant SARS-CoV-2 spike protein-encoding residues 1–1,138 (Wuhan-Hu-1; GenBank: MN908947.3) with proline substitutions at amino acids positions 986 and 987 and a “GSAS” substitution at the furin cleavage site (amino acid positions 682–682), was produced in ExpiCHO cells by transfection of 6 × 10^6^ cells/mL at 37°C with 0.8 g/mL of plasmid DNA using the ExpiCHO expression system transfection kit in ExpiCHO Expression Medium (ThermoFisher). After 1 day, the cells were refed, then incubated at 32°C for 11 days. The conditioned medium was mixed with cOmplete EDTA-free Protease Inhibitor (Roche). The recombinant protein was purified by chromatography on a Ni2+Sepharose 6 Fast Flow column (1 mL, GE LifeSciences). Samples were loaded in ExpiCHO Expression Medium supplemented with 30 mM imidazole, washed in a 20 mM Tris-HCl buffer (pH 7.4) containing 30 mM imidazole and 0.5 M NaCl. The recombinant protein was eluted with buffer containing 0.5 M NaCl and 0.3 M imidazole. The protein was further purified by size exclusion chromatography (HiLoad 16/60 Superdex 200, prep grade, GE LifeSciences) in 25 mM HEPES buffer (pH 7.5) containing 0.3 M NaCl.

### Biotinylation

For binding studies, recombinant SARS-CoV-2 spike protein was conjugated with EZ-Link Sulfo-NHS-Biotin (1:3 molar ratio; Thermo Fisher) in Dulbecco’s PBS at room temperature for 30 min. Glycine (0.1 M) was added to quench the reaction, and the buffer was exchanged for PBS using a Zeba spin column (Thermo Fisher).

### SARS-CoV-2 spike binding experiment

NCI-H1299 or A549 cells from the American Type Culture Collection were grown in RPMI medium containing 10% FBS and 100 U/mL penicillin and 100 µg/mL streptomycin sulfate under an atmosphere of 5% CO_2_ and 95% air. The cells at 50–80% confluence were lifted in 10 mM EDTA in PBS (Gibco) and washed in PBS containing 0.5% BSA. The cells were seeded into a 96-well plate at 10^5^ cells per well. The cells were then treated with a mix of 2.5 mU/mL *P. heparinus* Hsase II and 5 mU/mL *P. heparinus* Hsase III (IBEX Pharmaceuticals) in PBS containing 0.5% BSA (100 µL), 100 µL *B. ovatus*, and *thetaiotaomicron* or *E. coli* strain Nissle 1917 (ECN) minimal media culture cell-free supernatant supplemented with 10% BSA, or purified sulfatase or lyase enzymes at the given concentration for 30 min at 37°C. The cells were washed two times in PBS containing 0.5% BSA. The cells were then stained with 25 µg/mL biotinylated S protein (S1/S2) or 1:1,000 Anti-HS (Clone F58-10E4) (Fisher Scientific, NC1183789) in PBS containing 0.5% BSA, for 30 min at 4°C. The cells were washed two times and then stained with Streptavidin-Cy5 (Thermo Fisher) at 1:1,000 (S protein) or Anti-IgM-Alexa488 at 1:1,000 (Anti-HS) in PBS containing 0.5% BSA, for 30 min at 4°C. The cells were washed two times and then analyzed using a FACSCanto instrument (BD bioscience). Data analysis was performed using FlowJo, and statistical analyses were conducted in Prism 8.

### Recombinant sulfatase production

Genes coding for carbohydrate sulfatases *BT0756*, *BT1596*, *BT1624*, *BT3177*, *BT4655*, and *BT4656* from *B. thetaiotaomicron* were cloned into a pET28b or a pRSF plasmid with an *N*-terminal His_6_ TAG. The sulfatase-maturating enzyme (anSME) was systematically co-expressed with the different genes to ensure optimal maturation of the sulfatases (42, 78) except for BT4655, which is not predicted as a formylglycine-dependent sulfatase (42). For *BT0756*, *BT1596*, and *BT4656*, the serine residue, target of the post-translational modification catalyzed by anSME, was mutated into a cysteine residue in order to improve conversion into C_a_formylglycine, as previously reported (28). Plasmids were transformed into *E. coli* BL21(DE3) and the recombinant strains were grown in Luria-Bertani (LB) medium containing the suitable antibiotic (i.e., 100 µg/mL ampicillin or 50 µg/mL kanamycin, for pET28b or pRSF plasmids, respectively) at 37°C with an agitation speed of 170 rpm until OD_600_ reached 0.7. Protein expression was induced by adding 200 µM isopropyl β-d-thiogalactopyranoside and temperature decreased to 20°C for 18 h. Bacterial pellets were harvested by centrifugation (5,500 rpm, 10 min at 4°C) and suspended in buffer A: Tris 50 mM, KCl 100 mM, MgCl_2_ 10 mM, pH 8, supplemented with 1% vol/vol Triton X-100 and 2-mercaptoethanol. The cells were disrupted by sonication and supernatant clarified by ultracentrifugation (45,000 rpm, 1 h at 4°C). The supernatant was loaded onto a Ni-NTA column previously equilibrated with buffer A. The column was washed with 10 column volumes of buffer A and sulfatases were eluted with buffer A containing 300 mM imidazole. Imidazole was removed using a PD-10 desalting column equilibrated in buffer A. The sulfatase-containing fractions were concentrated with an Amicon concentrator (cutoff of 10  kDa, Millipore), and purity was assayed by SDS–PAGE and mass spectrometry analysis.

### Recombinant lyase BT4652 production

The *BT4652* lyase gene (synthesized by *GeneCust*) was ligated into a pET- His_6_-MBP-^(TEV)^ vector to produce BT4652 as a fusion protein with an His_6_-tagged MBP and a TEV cleavage sequence. *E. coli* BL21 (DE3) was transformed with the plasmid construct (pET- His_6_-MBP-^(TEV)^-BT4652) and overexpression was performed in LB medium containing 50 µg/mL kanamycin. Bacterial pellets were harvested by centrifugation and suspended in buffer B: Tris 50 mM, NaCl 300 mM, pH 8, supplemented with 1% vol/vol Triton X-100 and 2-mercaptoethanol. The cells were disrupted by sonication and supernatant clarified by ultracentrifugation (45,000 rpm, 1 h at 4°C). The supernatant was loaded onto an amylose resin column previously equilibrated with buffer B. The column was washed with 10 column volumes of buffer B and MBP-BT4652 protein eluted with the same buffer containing 10 mM maltose. Cleavage of the His_6_-MBP tag was performed using the His_6_-TEV protease overnight at 4°C. TEV protease and MBP protein were removed by purification on Ni-NTA column and BT4652 was further purified by size-exclusion chromatography using an ÅkTA system and a Superdex S200 column equilibrated with buffer B. Fractions of BT4652 lyase were analyzed by SDS–PAGE and those containing pure BT4652 were pooled and concentrated (Amicon concentrator, cutoff of 10 kDa, Millipore).

### Lyase expression in *E. coli* Nissle 1917 (EcN)

Lyases BT4675 and BT4662 were amplified from the genome of *B. thetaiotaomicron*, and inserted into pRSF-Duet using Golden Gate Assembly. The resulting plasmid was transformed into *E. coli* Nissle 1917 via electroporation. For lyase expression, a starter culture of *E. coli* Nissle 1917 containing the lyase plasmid was grown overnight in LB media containing 50 µg/mL of kanamycin, and then used to inoculate 500 mL of the same media. The cells were grown until they reached optical density 0.1–0.2, induced with 1 mM IPTG, and incubated overnight at room temperature. The cells were pelleted by centrifugation at 5,000 × *g* for 5 min, and the supernatant was removed for further experiments. The recombinant protein was purified by flowing over a Ni2+Sepharose 6 Fast Flow column (1 mL, GE Life Sciences), and eluted with 0.3 M imidazole, 0.5 M NaCl, and 25 mM HEPES pH 7.5. Protein was concentrated and buffer exchanged using a PES Pierce Protein concentrator (Thermo).

### Virus

All work with SARS-CoV-2 was conducted in Biosafety Level-3 conditions at the University of California San Diego following the guidelines approved by the Institutional Biosafety Committee. SARS-CoV-2 isolate WA1 (USA-WA1/2020, BEI NR-52281) was passaged through Caco2 cells and then expanded on TMPRSS2-VeroE6 cells. Supernatants were purified and stored at −80°C and titers were determined by fluorescent focus assay on TMPRSS2-VeroE6 cells. Virus stock was verified using deep sequencing analysis.

### Lyase assay

VeroE6 cells in 96-well plates were washed with DPBS and pretreated 1 h with *B. thet.* Purified BT4662 heparin lyase, *F. hep.* HSase, or matched volumes of vehicle (DPBS) in 50 µL of DMEM. The cells were then infected with authentic SARS-CoV-2 at a MOI of 0.5 for 30 min in the presence of enzyme or DPBS control. Virus was removed, and the cells were washed two times with 100 µL PBS and overlaid with methylcellulose (1% methylcellulose in MEM + 2% FBS, 1× Pen/Strep, 2 mM l-glutamine, and 1× non-essential amino acids). After 20 h of incubation, the cells were fixed for 30 min in 4% formaldehyde and stained with anti-nucleocapsid primary antibody (GeneTex, gtx135357) and anti-rabbit AlexaFluor 594 secondary with SytoxGreen nuclear counterstain. Plates were imaged on an Incucyte S3 imager. The total cell number and percent of cells infected were measured using the Incucyte onboard software tools.

## Data Availability

All American Gut Project sequence data and de-identified participant responses can be found in EBI under project PRJEB11419 and Qiita (https://qiita.ucsd.edu/) study ID 10317. The COVID-19 patient data are available through EBI under accession ERP124721 associated feature tables are publicly available in Qiita under study ID 13092. The FINRISK data that support the findings of this study are available from the THL Biobank based on a written application and following relevant Finnish legislation. Details of the application process are described on the website of the Biobank: https://thl.fi/en/web/thl-biobank/for-researchers. All of the data associated with this publication and in the California Teachers Study are available for research use. The California Teachers Study welcomes all such inquiries and encourages individuals to visit https://www.calteachersstudy.org/for-researchers. The source code for the analyses can be found at https://doi.org/10.5281/zenodo.3973506. The following reagent was deposited by the Centers for Disease Control and Prevention and obtained through BEI Resources, NIAID, NIH: SARS-Related Coronavirus 2, Isolate USA-WA1/2020, NR-52281.

## AFTER EPUB

*[This article was published on 25 February 2025 with an error in Michael Inouye's surname. The byline was corrected in the current version, posted on 20 March 2025.]*

## SUPPLEMENTAL MATERIAL

Supplemental Material mbio.04015-24-s0001.pdf Supplemental figures and tables.

ASM does not own the copyrights to Supplemental Material that may be linked to, or accessed through, an article. The authors have granted ASM a non-exclusive, world-wide license to publish the Supplemental Material files. Please contact the corresponding author directly for reuse.
