# Supplementary material for: SARS-CoV-2 infectivity can be modulated through bacterial grooming of the glycocalyx
Source: mBio. 2025 Feb 25;16(4):e04015-24. doi: 10.1128/mbio.04015-24 (PMC11980591; doi:10.1128/mbio.04015-24)
Supplement: Supplemental Material — Supplemental figures and tables. [file mbio.04015-24-s0001.pdf]

Supplementary Materials for

SARS-CoV-2 infectivity is modulated through bacterial grooming of the glycocalyx

Cameron Martino[1,2,3,†], Benjamin P. Kellman[1,2,†], Daniel R. Sandoval[4,†], Thomas Mandel Clausen[4,5,†], Robert Cooper[6], Alhosna Benjdia[7], Feryel Soualmia[7,8], Alex E. Clark[9], Aaron F. Garretson[9], Clarisse A. Marotz[1], Se Jin Song[3], Stephen Wandro[3], Livia S. Zaramela[1,10], Rodolfo A. Salido[1,3,6], Qiyun Zhu[1,11], Erick Armingol[1,2], Yoshiki Vázquez-Baeza[3,12], Daniel McDonald[1], James T. Sorrentino[1,2], Bryn Taylor[13], Pedro Belda-Ferre[1], Promi Das[1,14], Farhana Ali[1], Chenguang Liang[1,6,15], Yujie Zhang[6,16], Luca Schifanella[17,18], Alice Covizzi[19], Alessia Lai[19], Agostino Riva[19], Christopher Basting[17], Courtney Ann Broedlow[17], Aki S. Havulinna[20,21], Pekka Jousilahti[20], Mehrbod Estaki[1], Tomasz Kosciolek[1,22], Rayus Kuplicki[23], Teresa A. Victor[23], Martin P. Paulus[23], Kristen E. Savage[24], Jennifer L. Benbow[24,25], Emma S. Spielfogel[24], Cheryl A. M. Anderson[26], Maria Elena Martinez[26], James V. Lacey Jr[24], Shi Huang[1,3,27], Niina Haiminen[28], Laxmi Parida[28], Ho-Cheol Kim[29], Jack A. Gilbert[1,3,14], Daniel A. Sweeney[30], Sarah M. Allard[1,14], Austin D. Swafford[3,31], Susan Cheng[32,33], Michael Inoyue[34,35,36], Teemu Niiranen[20,37], Mohit Jain[38], Veikko Salomaa[20], Karsten Zengler[1,3,6], Nichole R. Klatt[17], Jeff Hasty[6,39], Olivier Berteau[7], Aaron F. Carlin[9], Jeffrey D. Esko[4,40,‡], Nathan E. Lewis[1,3,6,12,41,42,‡], Rob Knight[1,3,6,43,‡,*]

† These authors contributed equally to this work

‡ These authors contributed equally to this work

* Corresponding author: robknight@ucsd.edu

This PDF file includes:

Figs. S1 to S6
Tables S1 to S3

Supplemental Figure titles and legends

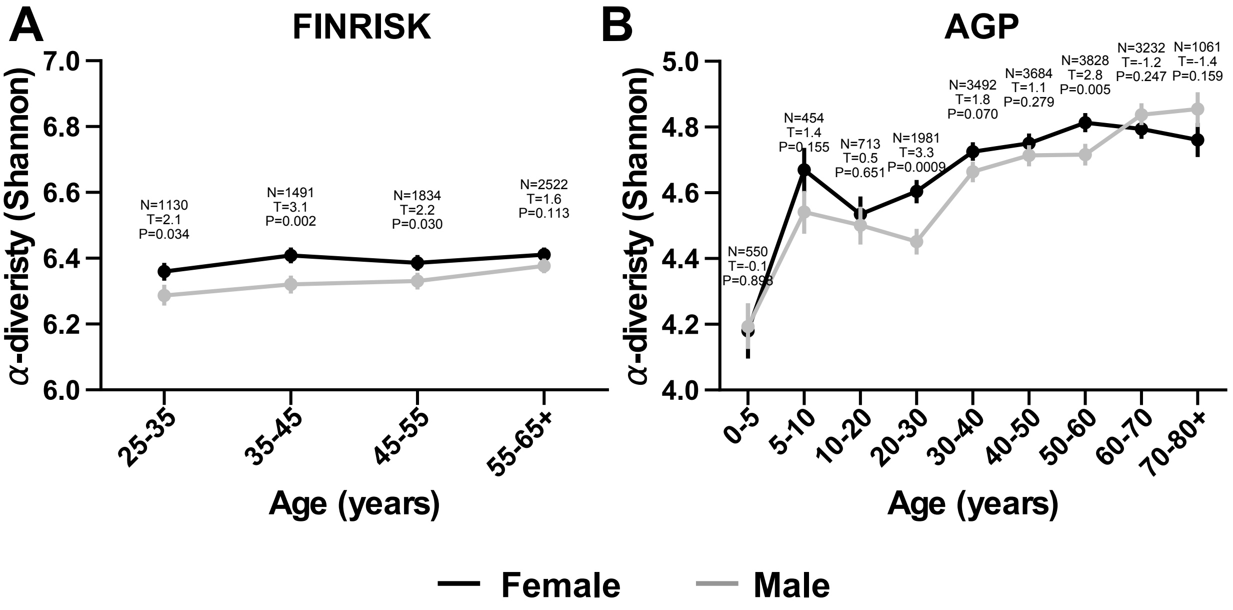

Supplemental Figure 1. **Shannon alpha diversity (y-axes) changes by age (x-axes) and colored by host sex being women (black) and men (gray) in FINRISK (A) and AGP (B) datasets.**

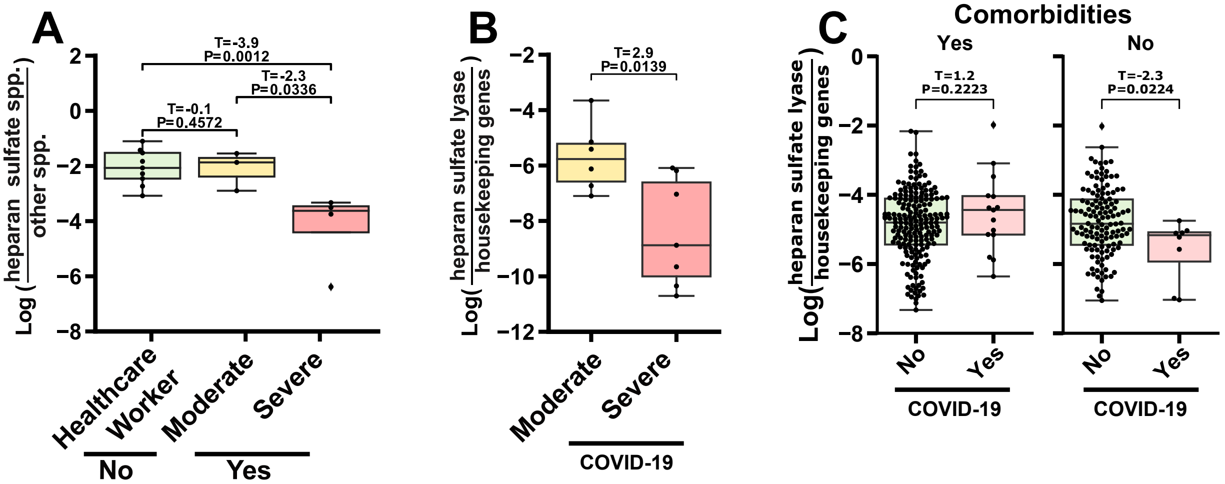

Supplemental Figure 2. HS-modifying bacteria are inversely enriched by COVID-19 disease severity. The log-ratio of predicted HS-modifying species relative to those with no predicted capacity in a cohort of healthcare workers and COVID-19 patients fecal microbiomes split by disease severity (**A**). The log-ratio of HS lyase genes relative to a set of housekeeping genes (y-axis) in a second cohort of COVID-19 patients by disease severity (**B**). The log-ratio of HS lyase genes relative to a set of housekeeping genes (y-axis) in AGP survey data by COVID-19 outcomes (x-axes) split by existing comorbidities (**C**). All log-ratio plots across age annotated by the number of subjects at that time point. Error bars represent the standard error of the mean. Presented p-values and test statistics are from unpaired two-tailed t-test evaluated on each host age group between host sex

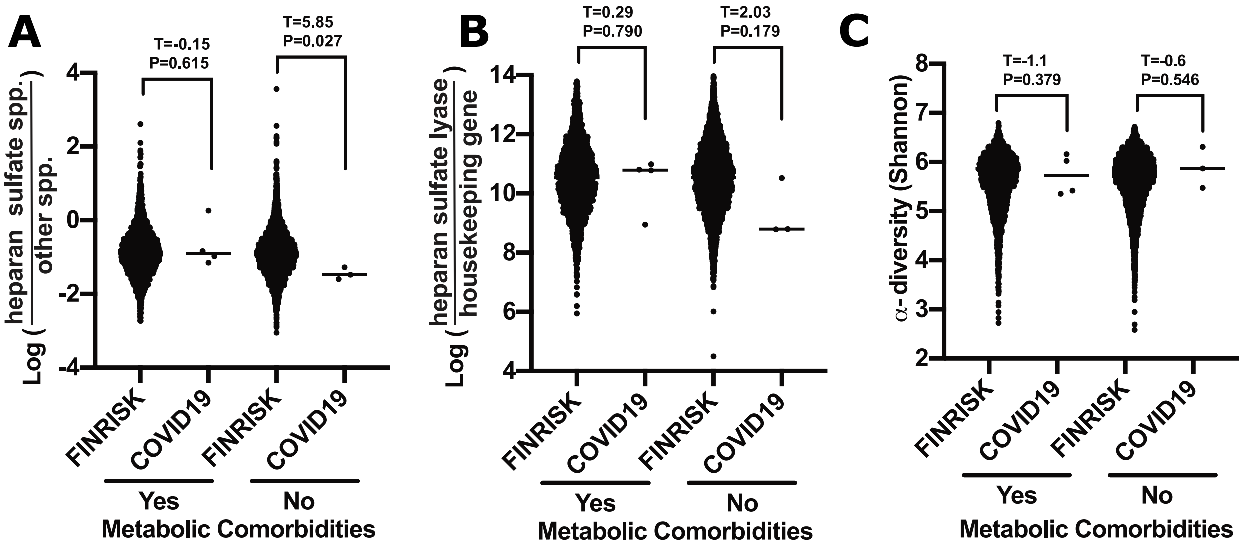

Supplemental Figure 3. HS-modifying bacteria depleated in FINRISK 2002 subjects prior to SARS-CoV-2 infection. Log-ratio of predicted HS-modifying species relative to those with no predicted capacity (**A**), HS lyase genes relative to a set of housekeeping genes (**B**), and Shannon alpha-diversity (**C**) in FINRISK 2002 dataset by subsequent COVID-19 outcomes split

by existing comorbidities. Presented p-values and test statistics are from unpaired two-tailed t-test.

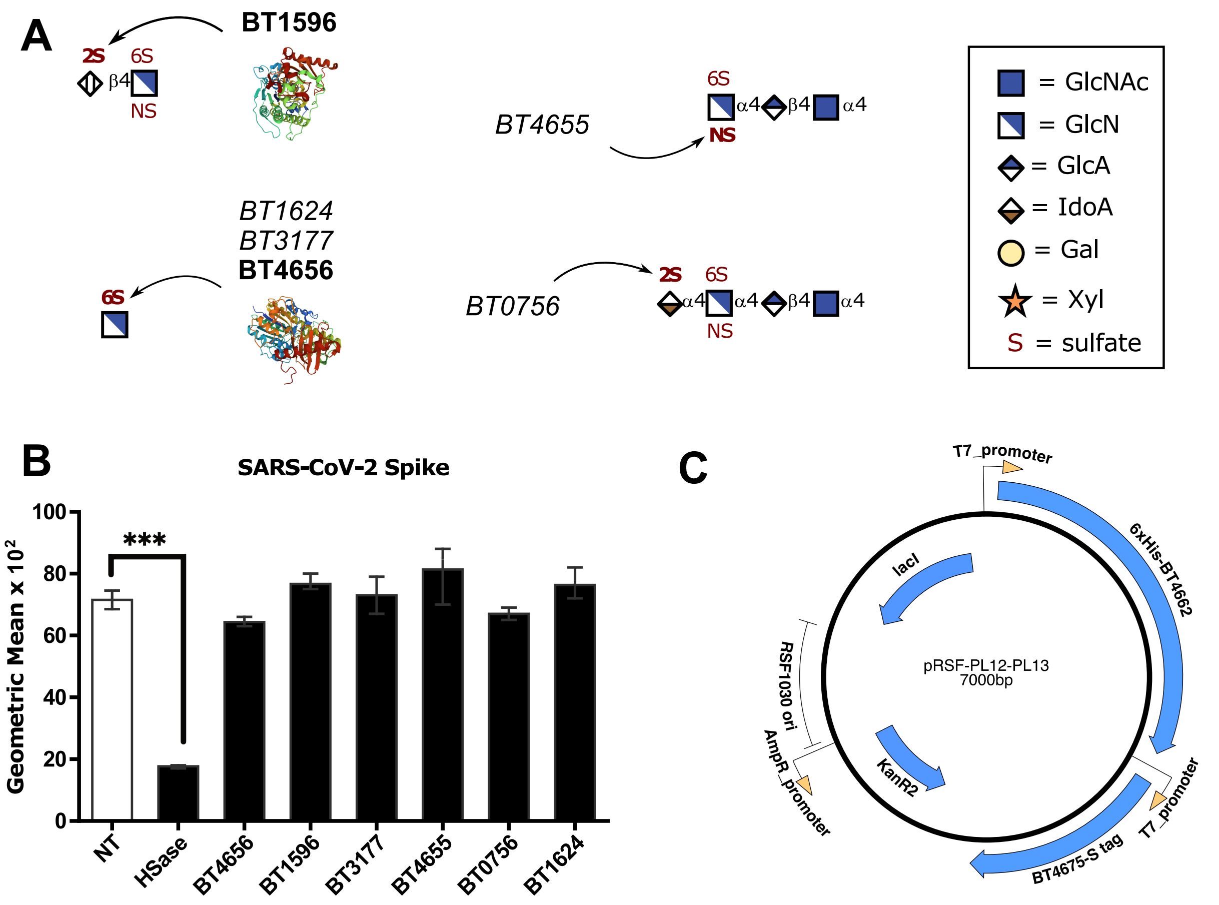

Supplemental Figure 4. Commensal human gut sulfatases do not block SARS-CoV-2 spike protein binding alone. Schematic diagram of activity and specificity of sulfatases (**A**). Geometric mean of FACS count data (y-axis) of cultured human cells incubated with biotinylated SARS-CoV-2 spike protein after no treatment (NT), treatment with HSase from *Pedobacter heparinus* (*P. hep.*), or sulfatases (BT4656, BT1596, BT3177, BT4655, BT0756, BT1624) (**B**). Plasmid diagram for endo-lyases BT4662 and BT4675 co-expressed in *Escherichia coli* strain Nissle **1917** (EcN) (**C**).

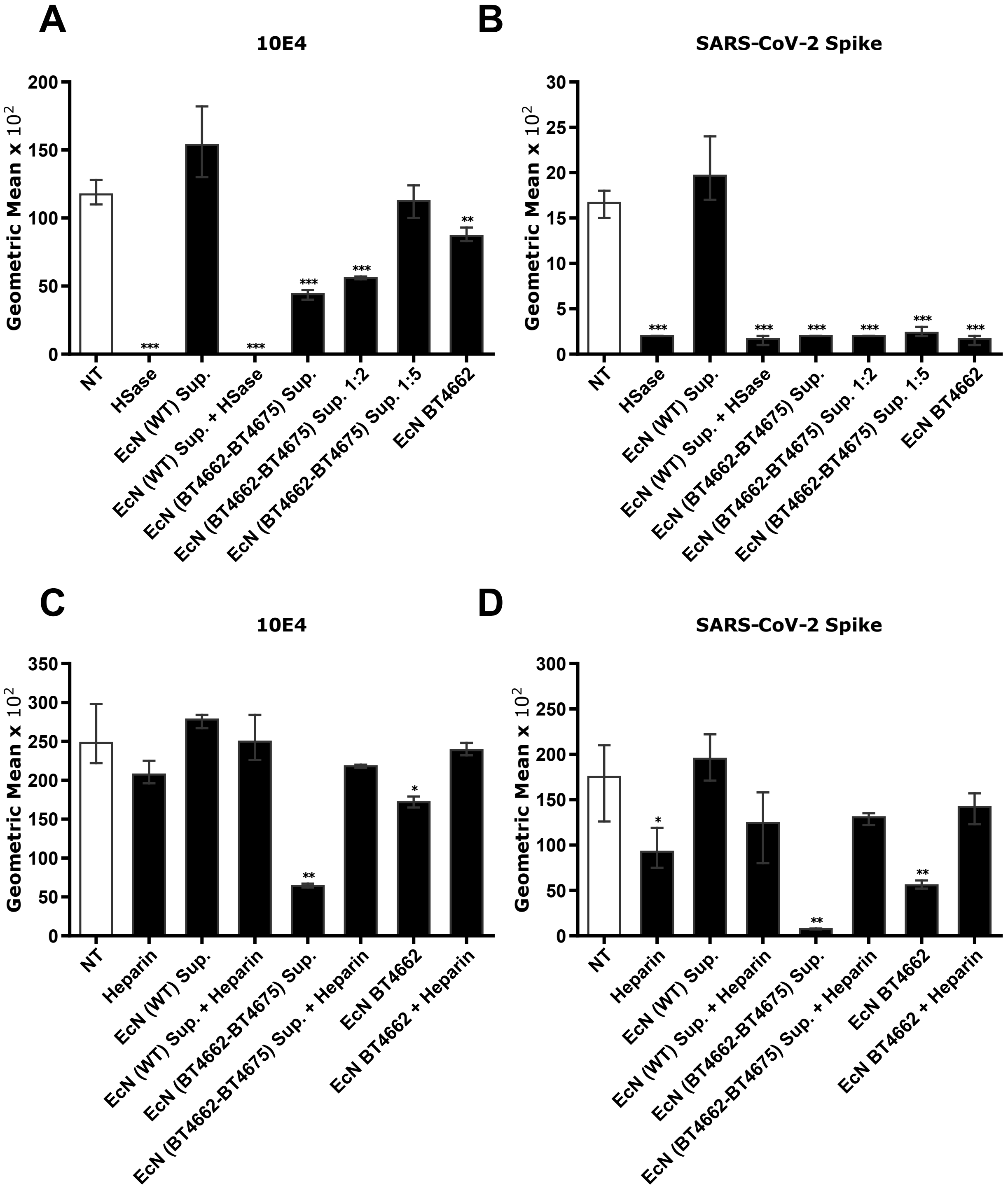

Supplemental Figure 5. **Geometric mean of flow cytometry data of EcN expressed HS lyase data**. Geometric mean of fow cytometry data (y-axis) of cultured human A549 cells stained with the HS antibody 10E4 (**A and C**) or incubated with biotinylated SARS-CoV-2 spike protein (B

and **D**), with no treatment (NT) or treated with purified HSase from *Pedobacter heparinus* (*P. hep.*), supernatant from EcN wild type (WT) or EcN (BT4662-BT4675), or purified BT4662 with and without heparin competition during the enzymatic digestion. Presented p-values are from unpaired t-test statistics w.r.t. NT ($p \leq 0.05$ [*], $p \leq 0.01$ [**], $p \leq 0.001$ [***]).

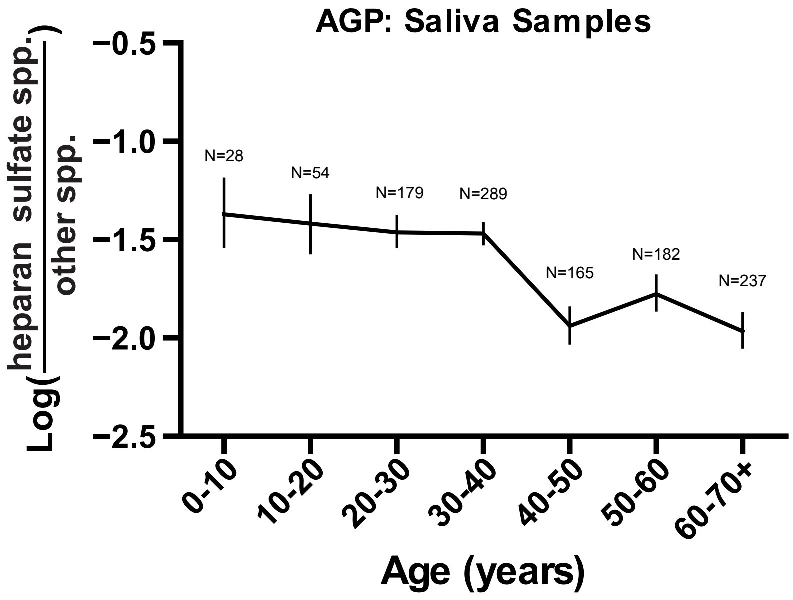

Supplemental Figure 6. The log-ratio of predicted HS-modifying species relative to those with no predicted capacity for (y-axes) AGP saliva samples across age (x-axis).

Supplemental Table titles and legends

Supplemental Table 1. COVID-19 risk factors in relation to the log-ratio of predicted HS-modifying species relative to those with no predicted capacity assessed by OLS-regression in the FINRISK 2002 dataset.

	**Coef.**	**Std.Err.**	**t**	**P>|t|**	**[0.025**	**0.975]**
**Age**	-0.0107	0.0038	-2.8488	0.0044	-0.0181	-0.0033
**Sex**	-0.0802	0.0152	-5.2847	1.30E-07	-0.1099	-0.0504
**Diabetes**	0.0824	0.0823	1.0013	0.3167	-0.0789	0.2438
**Ex-Smoker**	-0.1732	0.0864	-2.0054	0.045	-0.3425	-0.0039
**BMI**	-0.003	0.007	-0.4327	0.6653	-0.0168	0.0107
**Asthma**	0.0246	0.0917	0.2679	0.7888	-0.1552	0.2044
**Cancer**	-0.2692	0.1567	-1.7176	0.0859	-0.5764	0.038
**Cardiovascular Disease**	0.0615	0.1891	0.3253	0.745	-0.3091	0.4321
**Liver Fibrosis or Cirrhosis**	-1.9724	1.1363	-1.7358	0.0826	-4.1998	0.2551
**Autoimmune Disease**	0.2728	0.1829	1.4913	0.1359	-0.0858	0.6314

Supplemental Table 2. Pearson correlation shows a weak correlation between alpha diversity (Shannon) and heparan sulfate modifying log-ratio split by host sex.

	**FINRISK**		**AGP**	
	**Men**	**Women**	**Men**	**Women**
**R**	-0.364	-0.473	-0.0005	-0.157
**p-value**	6.83E-102	5.88E-221	0.964	1.91E-57

Supplemental Table 3. Table of CAZy ID associated with heparan sulfate modification tasks for each bacterial species along with the capacity and completeness for a given species.

**species**	**CAZy ID**	**capacity**	**completeness**
Teredinibacter turnerae	GH79	2.455699954	1

Brachybacterium faecium	GH79	2.322733424	1
Novosphingobium aromaticivorans	GH79	0.766564697	0
Solibacter usitatus	GH79	3.694382176	1
Saccharophagus degradans	GH79	2.105502422	0
Bradyrhizobium diazoefficiens	GH79	3.166834227	1
Burkholderia pseudomallei	GH79	3.93219767	1
Acidobacterium capsulatum	GH79	2.325925309	1
Burkholderia mallei	GH79	3.727548914	1
Catenulispora acidiphila	GH79	2.886534124	1
Streptococcus uberis	GH79	4.209153639	1
Burkholderia pseudomallei	GH79	3.93219767	1
Solibacter usitatus	GH79	3.694382176	1
Burkholderia pseudomallei	GH79	3.93219767	1
Burkholderia pseudomallei	GH79	3.93219767	1
Catenulispora acidiphila	GH79	2.886534124	1
Sediminispirochaeta smaragdinae	GH79	4.100615895	1
Marinomonas sp.	GH79	3.075451459	1
Caulobacter segnis	GH2	2.163370478	1
Chitinophaga pinensis	GH2	3.132181861	1
Solibacter usitatus	GH2	3.694382176	1
Ochrobactrum anthropi	GH2	3.03545983	1
Erwinia sp.	GH2	2.919717144	1
Bifidobacterium bifidum	GH2	5.013833965	1
Pedobacter heparinus	GH2	3.537943992	1
Escherichia coli	GH2	4.951896641	1
Thermotoga naphthophila	GH2	2.301883351	1
Escherichia coli	GH2	4.951896641	1
Enterobacter agglomerans	GH2	1.327178123	0
Shigella boydii	GH2	4.467400665	1
Opitutus terrae	GH2	2.849000161	1
Roseburia intestinalis	GH2	5.494855325	1
Escherichia coli	GH2	4.951896641	1
Streptomyces scabiei	GH2	2.583957419	1
Bacteroides fragilis	GH2	5.330440553	1
Geobacter bemidjiensis	GH2	2.993336071	1
Butyrivibrio proteoclasticus	GH2	4.213375923	1
Prevotella ruminicola	GH2	4.464744795	1
Bacteroides fragilis	GH2	5.330440553	1
Escherichia coli	GH2	4.951896641	1
Escherichia coli	GH2	4.951896641	1

Nostoc punctiforme	GH2	2.376682258	1
Bacteroides fragilis	GH2	5.330440553	1
Deinococcus geothermalis	GH2	2.528732653	1
Thermobaculum terrenum	GH2	2.7917651	1
Shigella dysenteriae	GH2	4.389565899	1
Ruminiclostridium cellulolyticum	GH2	4.261509628	1
Escherichia coli	GH2	4.951896641	1
Nocardiopsis dassonvillei	GH2	2.731716088	1
Shigella flexneri	GH2	4.384218202	1
Roseburia intestinalis	GH2	5.494855325	1
Escherichia coli	GH2	4.951896641	1
Bifidobacterium bifidum	GH2	5.013833965	1
Streptococcus mitis	GH1	4.432157949	1
Lactobacillus plantarum	GH2	3.483288678	1
Flavobacterium johnsoniae	GH2	3.525155341	1
Parabacteroides distasonis	GH2	5.5003263	1
Paenibacillus sp.	GH2	3.716301192	1
Faecalibacterium prausnitzii	GH2	5.703104557	1
Escherichia coli	GH2	4.951896641	1
Lactobacillus helveticus	GH2	4.191208361	1
Pseudothermotoga lettingae	GH2	2.003060928	1
Shewanella amazonensis	GH2	2.21211605	1
Geobacillus sp.	GH2	3.64830473	1
Streptococcus pneumoniae	GH2	3.898009613	1
Bifidobacterium bifidum	GH2	5.013833965	1
Yersinia pestis	GH2	3.433167805	1
Escherichia coli	GH2	4.951896641	1
Listeria monocytogenes	GH1	2.652162063	1
Geobacter sp.	GH2	3.054419337	1
Dyadobacter fermentans	GH2	3.417983563	1
Kribbella flavida	GH2	2.574757512	1
Escherichia coli	GH2	4.951896641	1
Bacteroides thetaiotaomicron	GH2	5.668359112	1
Solibacter usitatus	GH2	3.694382176	1
Shigella dysenteriae	GH2	4.389565899	1
Capnocytophaga ochracea	GH2	3.168311961	1
Paraburkholderia phytofirmans	GH2	3.21698638	1
Frankia sp.	GH2	2.890273357	1
Sinorhizobium medicae	GH2	2.840198453	1
Escherichia coli	GH2	4.951896641	1

Cronobacter sakazakii	GH2	3.663917102	1
Xylanimonas cellulosilytica	GH2	3.169152732	1
Gramella forsetii	GH2	3.311848451	1
Faecalibacterium prausnitzii	GH2	5.703104557	1
Streptococcus thermophilus	GH2	3.238181275	1
Bacteroides xylanisolvens	GH2	5.755848669	1
Pantoea ananatis	GH2	3.19668615	1
Brevundimonas subvibrioides	GH2	2.525486274	1
Parabacteroides distasonis	GH2	5.5003263	1
Bifidobacterium longum	GH2	5.049759625	1
Ruminococcus gnavus	GH2	5.39652351	1
Escherichia coli	GH2	4.951896641	1
Pediococcus pentosaceus	GH1	3.700450677	1
Shigella flexneri	GH1	4.384218202	1
Coraliomargarita akajimensis	GH2	3.018355261	1
Roseburia intestinalis	GH2	5.494855325	1
Escherichia coli	GH2	4.951896641	1
Prevotella ruminicola	GH2	4.464744795	1
Escherichia coli	GH2	4.951896641	1
Bacteroides thetaiotaomicron	GH2	5.668359112	1
Clostridium saccharolyticum	GH2	5.056490176	1
Geobacillus sp.	GH2	3.64830473	1
Anaerostipes hadrus	GH1	5.394053782	1
Clostridium acetobutylicum	GH1	1.970128234	0
Paenibacillus polymyxa	GH1	3.727193955	1
Escherichia coli	GH1	4.951896641	1
Streptococcus pyogenes	GH1	4.645909728	1
Streptococcus pyogenes	GH1	4.645909728	1
Azospirillum sp.	GH2	2.549653156	1
Bacteroides xylanisolvens	GH2	5.755848669	1
Flavobacterium johnsoniae	GH2	3.525155341	1
Lachnoclostridium phytofermentans	GH2	4.440620629	1
Clostridioides difficile	GH1	5.039965306	1
Leuconostoc citreum	GH1	3.622436533	1
Thermoanaerobacter pseudethanolicus	GH2	3.370655894	1
Thermotoga neapolitana	GH2	2.194896294	1
Paenibacillus polymyxa	GH2	3.727193955	1
Paenarthrobacter aurescens	GH2	2.654575837	1
Dickeya dadantii	GH1	3.173053214	1
Bacillus subtilis	GH1	3.020649231	1

Xanthomonas campestris	GH2	2.836321535	1
Stackebrandtia nassauensis	GH30	2.705407885	1
Roseburia intestinalis	GH2	5.494855325	1
Spirochaeta thermophila	GH2	3.412755316	1
Escherichia coli	GH2	4.951896641	1
Paenibacillus polymyxa	GH2	3.727193955	1
Bacteroides fragilis	GH2	5.330440553	1
Clostridium cellulovorans	GH2	4.295967255	1
Citrobacter rodentium	GH2	4.007244277	1
Bacteroides vulgatus	GH2	5.981194327	1
Capnocytophaga ochracea	GH2	3.168311961	1
Lactobacillus crispatus	GH2	3.911834731	1
Shewanella sediminis	GH2	1.925319232	0
Geobacillus thermodenitrificans	GH1	3.274686288	1
Xanthomonas campestris	GH2	2.836321535	1
Pectobacterium carotovorum	GH1	3.146442611	1
Escherichia coli	GH2	4.951896641	1
Bacteroides xylanisolvens	GH2	5.755848669	1
Opitutus terrae	GH2	2.849000161	1
Escherichia coli	GH2	4.951896641	1
Marinomonas sp.	GH79	3.075451459	1
Streptococcus pneumoniae	GH1	3.898009613	1
Lactobacillus johnsonii	GH1	3.821988045	1
Clostridium saccharolyticum	GH1	5.056490176	1
Escherichia coli	GH1	4.951896641	1
Magnetospirillum magneticum	GH1	2.983954556	1
Clostridium beijerinckii	GH1	4.172192327	1
Dinoroseobacter shibae	GH1	2.433766048	1
Pedobacter heparinus	GH2	3.537943992	1
Streptococcus pneumoniae	GH1	3.898009613	1
Listeria innocua	GH1	2.642937634	1
Streptococcus agalactiae	GH1	2.443350463	1
Streptomyces coelicolor	GH1	1.758481979	1
Thermobispora bispora	GH1	2.603002819	1
Salinispora tropica	GH1	2.512074083	1
[Eubacterium] rectale	GH2	5.645131414	1
Pedobacter heparinus	GH2	3.537943992	1
Bacteroides fragilis	GH2	5.330440553	1
Akkermansia muciniphila	GH2	5.317459779	1
Actinomyces naeslundii	GH1	1.975379698	0

Escherichia coli	GH2	4.951896641	1
Segniliparus rotundus	GH2	2.392276228	1
Exiguobacterium sp.	GH1	3.604041401	1
Butyrivibrio fibrisolvens	GH1	4.079675342	1
Rhizobium leguminosarum	GH2	2.765664497	1
Kribbella flavida	GH2	2.574757512	1
Bacillus velezensis	GH30	3.007591831	1
Streptococcus pneumoniae	GH2	3.898009613	1
Agrobacterium radiobacter	GH2	2.773077398	1
Enterococcus faecalis	GH2	4.493392116	1
Xanthomonas axonopodis	GH2	1.902261106	1
Dickeya paradisiaca	GH1	3.018719322	1
Citrobacter freundii	GH2	2.755642327	1
Streptococcus pyogenes	GH1	4.645909728	1
Xanthomonas campestris	GH30	2.836321535	1
Streptococcus thermophilus	GH2	3.238181275	1
Bacteroides xylanisolvens	GH2	5.755848669	1
Escherichia coli	GH2	4.951896641	1
Prevotella melaninogenica	GH2	4.645622238	1
Aliivibrio fischeri	GH2	2.148976013	1
Roseburia intestinalis	GH2	5.494855325	1
Dictyoglomus thermophilum	GH2	2.597989089	1
Bacteroides vulgatus	GH2	5.981194327	1
Klebsiella pneumoniae	GH2	3.527499461	1
Bifidobacterium bifidum	GH2	5.013833965	1
Clostridium perfringens	GH2	4.092439715	1
Rhizobium leguminosarum	GH2	2.765664497	1
Cupriavidus metallidurans	GH2	2.960344803	1
Bacteroides thetaiotaomicron	GH2	5.668359112	1
Klebsiella pneumoniae	GH2	3.527499461	1
Vibrio vulnificus	GH2	1.625869887	0
Bacteroides xylanisolvens	GH2	5.755848669	1
Bacteroides xylanisolvens	GH2	5.755848669	1
Burkholderia ambifaria	GH2	2.822519768	1
Streptococcus suis	GH2	4.75869111	1
Bacteroides vulgatus	GH2	5.981194327	1
Bacteroides fragilis	GH2	5.330440553	1
Shigella flexneri	GH2	4.384218202	1
Bacteroides fragilis	GH2	5.330440553	1
Opitutus terrae	GH2	2.849000161	1

Sebaldella termitidis	GH1	2.899978786	1
Escherichia coli	GH2	4.951896641	1
Spirochaeta thermophila	GH30	3.412755316	1
Shigella flexneri	GH1	4.384218202	1
Paenibacillus sp.	GH30	3.716301192	1
Zunongwangia profunda	GH30	3.493179318	1
Bacteroides fragilis	GH2	5.330440553	1
Streptococcus mitis	GH2	4.432157949	1
Chitinophaga pinensis	GH2	3.132181861	1
Opitutus terrae	GH2	2.849000161	1
Streptococcus thermophilus	GH2	3.238181275	1
Ruegeria sp.	GH1	2.923411866	1
Lactococcus lactis	GH1	4.535318878	1
Streptomyces bingchenggensis	GH2	2.546261927	1
Frankia sp.	GH2	2.890273357	1
Kineococcus radiotolerans	GH2	3.03645742	1
Agrobacterium radiobacter	GH2	2.773077398	1
Bacteroides fragilis	GH2	5.330440553	1
Catenulispora acidiphila	GH2	2.886534124	1
Escherichia coli	GH2	4.951896641	1
Frankia sp.	GH1	2.890273357	1
Nitrosospira multiformis	GH2	3.126506459	1
Flavobacterium johnsoniae	GH2	3.525155341	1
Agrobacterium fabrum	GH2	2.626901456	1
Thermoanaerobacter pseudethanolicus	GH2	3.370655894	1
Bacteroides thetaiotaomicron	GH2	5.668359112	1
Shigella boydii	GH2	4.467400665	1
Paenibacillus sp.	GH2	3.716301192	1
Lactococcus lactis	GH1	4.535318878	1
Lactobacillus johnsonii	GH1	3.821988045	1
Opitutus terrae	GH2	2.849000161	1
Bacteroides xylanisolvens	GH2	5.755848669	1
Thermoanaerobacter italicus	GH2	3.176195199	1
Maribacter sp.	GH2	3.442416285	1
Escherichia coli	GH2	4.951896641	1
Paraburkholderia phymatum	GH1	0.762869044	0
Streptomyces bingchenggensis	GH1	2.546261927	1
Citrobacter rodentium	GH1	4.007244277	1
Flavobacterium johnsoniae	GH1	3.525155341	1
Burkholderia mallei	GH2	3.727548914	1

Shigella boydii	GH2	4.467400665	1
Escherichia coli	GH2	4.951896641	1
Bacteroides thetaiotaomicron	GH2	5.668359112	1
Caulobacter sp.	GH30	2.573978441	1
Bacteroides thetaiotaomicron	GH2	5.668359112	1
Erwinia amylovora	GH2	2.90156558	1
Clostridium saccharolyticum	GH1	5.056490176	1
Zunongwangia profunda	GH2	3.493179318	1
Pseudoalteromonas atlantica	GH2	2.341949533	1
Bacteroides vulgatus	GH30	5.981194327	1
Xanthomonas campestris	GH30	2.836321535	1
Chitinophaga pinensis	GH30	3.132181861	1
Bacteroides vulgatus	GH2	5.981194327	1
Geobacillus kaustophilus	GH1	3.176799249	1
Burkholderia sp.	GH1	3.185120973	1
Caulobacter sp.	GH2	2.573978441	1
Bacteroides vulgatus	GH2	5.981194327	1
Bifidobacterium bifidum	GH1	5.013833965	1
Shewanella pealeana	GH2	2.21293979	1
Vibrio cholerae	GH2	2.401983537	1
Stackebrandtia nassauensis	GH30	2.705407885	1
Streptococcus pneumoniae	GH1	3.898009613	1
Leuconostoc kimchii	GH1	3.578591417	1
Lactobacillus paracasei	GH1	4.039963241	1
Herpetosiphon aurantiacus	GH1	2.467219737	1
Xylanimonas cellulosilytica	GH1	3.169152732	1
Streptococcus dysgalactiae	GH1	3.643525026	1
Bacillus megaterium	GH1	3.09272887	1
Escherichia coli	GH1	4.951896641	1
Actinobacillus succinogenes	GH1	2.684338084	1
Spiroplasma citri	GH1	1.093054293	1
Erwinia amylovora	GH1	2.90156558	1
Erwinia sp.	GH1	2.919717144	1
Bacteroides thetaiotaomicron	GH2	5.668359112	1
Bacteroides vulgatus	GH2	5.981194327	1
Enterobacter lignolyticus	GH2	3.968988727	1
Aeromonas hydrophila	GH1	3.103122052	1
Streptococcus pneumoniae	GH1	3.898009613	1
Bacteroides xylanisolvens	GH2	5.755848669	1
Cytophaga hutchinsonii	GH1	3.438614745	1

Streptomyces ambofaciens	GH2	2.51396916	1
Paenibacillus sp.	GH2	3.716301192	1
Akkermansia muciniphila	GH2	5.317459779	1
Paludibacter propionicigenes	GH2	3.910160743	1
Clostridium acetobutylicum	GH30	1.970128234	0
Acidothermus cellulolyticus	GH1	2.744507416	1
Bacteroides vulgatus	GH2	5.981194327	1
Streptococcus pneumoniae	GH1	3.898009613	1
Escherichia coli	GH1	4.951896641	1
[Eubacterium] rectale	GH2	5.645131414	1
Kribbella flavida	GH2	2.574757512	1
Anaerostipes hadrus	GH1	5.394053782	1
Streptococcus pneumoniae	GH1	3.898009613	1
Bacteroides thetaiotaomicron	GH2	5.668359112	1
Bacteroides thetaiotaomicron	GH2	5.668359112	1
Actinosynnema mirum	GH2	2.698718791	1
Roseburia intestinalis	GH1	5.494855325	1
Bifidobacterium longum	GH30	5.049759625	1
Pseudescherichia vulneris	GH2	1.369488818	0
Paenibacillus sp.	GH30	3.716301192	1
Stigmatella aurantiaca	GH2	2.897849609	1
Anoxybacillus flavithermus	GH1	3.291368113	1
Prevotella melaninogenica	GH2	4.645622238	1
Bradyrhizobium diazoefficiens	GH79	3.166834227	1
Burkholderia mallei	GH79	3.727548914	1
Saccharophagus degradans	GH79	2.105502422	0
Catenulispora acidiphila	GH79	2.886534124	1
Solibacter usitatus	GH79	3.694382176	1
Acidobacterium capsulatum	GH79	2.325925309	1
Escherichia coli	GH2	4.951896641	1
Escherichia coli	GH2	4.951896641	1
Paludibacter propionicigenes	GH2	3.910160743	1
Escherichia coli	GH2	4.951896641	1
Agathobacter rectalis	GH2	5.641970263	1
Flavobacterium johnsoniae	GH2	3.525155341	1
Zunongwangia profunda	GH2	3.493179318	1
Maribacter sp.	GH2	3.442416285	1
Leadbetterella byssophila	GH2	3.698360007	1
Serratia proteamaculans	GH2	3.231309256	1
Oenococcus oeni	GH2	3.610261575	1

Brachyspira pilosicoli	GH2	3.888206756	1
Pseudescherichia vulneris	GH2	1.369488818	0
Cutibacterium acnes	GH2	3.525541687	1
Paenibacillus sp.	GH2	3.716301192	1
Dyadobacter fermentans	GH2	3.417983563	1
Lactobacillus sakei	GH2	3.906365165	1
Xanthomonas campestris	GH30	2.836321535	1
Spirochaeta thermophila	GH2	3.412755316	1
Streptococcus pneumoniae	GH1	3.898009613	1
Agrobacterium vitis	GH2	2.861500523	1
Shigella flexneri	GH2	4.384218202	1
Streptosporangium roseum	GH1	2.74523594	1
Staphylococcus aureus	GH1	3.253816682	1
Rhizobium etli	GH1	2.801391781	1
Oenococcus oeni	GH1	3.610261575	1
Clostridioides difficile	GH1	5.039965306	1
Bacillus pseudofirmus	GH1	3.078819641	1
Escherichia coli	GH2	4.951896641	1
Saccharopolyspora erythraea	GH30	2.829954331	1
Bacteroides fragilis	GH2	5.330440553	1
Lactobacillus gasseri	GH1	2.459107723	1
Klebsiella pneumoniae	GH1	3.527499461	1
Haliangium ochraceum	GH1	2.906275029	1
Clostridium botulinum	GH1	4.053974807	1
Yersinia enterocolitica	GH1	3.206616339	1
Streptomyces griseus	GH1	2.688132045	1
Photorhabdus laumondii	GH1	2.248422138	1
Bradyrhizobium sp.	GH1	2.919717144	1
Bacillus pumilus	GH1	3.248670682	1
Chitinophaga pinensis	GH2	3.132181861	1
Bacillus halodurans	GH2	3.382316202	1
Caulobacter sp.	GH1	2.573978441	1
Staphylococcus saprophyticus	GH1	3.069986965	1
Erwinia billingiae	GH1	3.247037685	1
Lactococcus garvieae	GH1	2.40846932	1
Lactobacillus amylovorus	GH1	4.651593108	1
uncultured bacterium	GH2	4.006358463	1
Lactobacillus salivarius	GH2	4.268234604	1
Acidobacterium capsulatum	GH2	2.325925309	1
Streptococcus pyogenes	GH2	4.645909728	1

Paenibacillus polymyxa	GH1	3.727193955	1
Shewanella baltica	GH1	2.273258695	1
Thermoanaerobacterium thermosaccharolyticum	GH1	3.608969791	1
Thermomonospora curvata	GH1	2.806528372	1
Rhizobium etli	GH2	2.801391781	1
Mobiluncus curtisii	GH2	4.209484221	1
Pectobacterium carotovorum	GH2	3.146442611	1
Zunongwangia profunda	GH30	3.493179318	1
Opitutus terrae	GH2	2.849000161	1
Sorangium cellulosum	GH1	2.502583787	1
Sanguibacter keddieii	GH1	2.855701308	1
Escherichia coli	GH2	4.951896641	1
Paenibacillus polymyxa	GH1	3.727193955	1
Shigella boydii	GH2	4.467400665	1
Bacteroides xylanisolvens	GH2	5.755848669	1
Escherichia coli	GH2	4.951896641	1
Exiguobacterium sp.	GH1	3.604041401	1
Flavobacterium johnsoniae	GH30	3.525155341	1
Escherichia coli	GH2	4.951896641	1
Lactococcus garvieae	GH1	2.40846932	1
Yersinia enterocolitica	GH1	3.206616339	1
Dickeya chrysanthemi	GH2	2.985934376	1
Staphylococcus carnosus	GH2	3.461188466	1
Bacteroides xylanisolvens	GH2	5.755848669	1
Arcanobacterium haemolyticum	GH2	3.045776263	1
Prevotella ruminicola	GH2	4.464744795	1
Bacteroides fragilis	GH2	5.330440553	1
Kribbella flavida	GH2	2.574757512	1
Bifidobacterium animalis	GH2	4.326031942	1
Salmonella typhimurium	GH1	3.278675607	1
Streptococcus suis	GH1	4.75869111	1
Clavibacter michiganensis	GH2	3.019422612	1
Pseudothermotoga lettingae	GH2	2.003060928	1
Lactococcus lactis	GH2	4.535318878	1
Bacteroides thetaiotaomicron	GH2	5.668359112	1
Escherichia coli	GH2	4.951896641	1
Streptomyces scabiei	GH2	2.583957419	1
Bacteroides fragilis	GH2	5.330440553	1
Streptococcus thermophilus	GH2	3.238181275	1
Bacteroides fragilis	GH2	5.330440553	1

Lactobacillus crispatus	GH2	3.911834731	1
Shigella boydii	GH2	4.467400665	1
Edwardsiella ictaluri	GH2	3.122548676	1
Burkholderia vietnamiensis	GH2	2.715571066	1
Paraburkholderia xenovorans	GH1	3.175892682	1
Thermoanaerobacter mathranii	GH2	3.212248516	1
Paenibacillus sp.	GH2	3.716301192	1
Frankia inefficax	GH2	2.921941994	1
Ruegeria sp.	GH2	2.923411866	1
Actinobacillus succinogenes	GH2	2.684338084	1
Caldicellulosiruptor obsidiansis	GH2	3.232298508	1
Burkholderia lata	GH2	2.633788296	1
Neisseria lactamica	GH2	3.176069844	1
Lactobacillus delbrueckii	GH2	4.330538982	1
Leuconostoc mesenteroides	GH2	4.403938925	1
Bacillus megaterium	GH1	3.09272887	1
Blautia obeum	GH1	5.41829818	1
Kineococcus radiotolerans	GH1	3.03645742	1
Caldicellulosiruptor kronotskyensis	GH2	3.162669177	1
Streptococcus suis	GH1	4.75869111	1
Streptomyces griseus	GH1	2.688132045	1
Streptosporangium roseum	CBM57	2.74523594	1
Spiroplasma citri	GH1	1.093054293	1
Starkeya novella	GH1	2.487372647	1
Bacillus subtilis	GH1	3.020649231	1
Caldicellulosiruptor kristjanssonii	GH1	3.125858087	1
Nakamurella multipartita	GH1	2.617574677	1
Cronobacter sakazakii	GH1	3.663917102	1
Gloeothece verrucosa	GH1	2.385281292	1
Enterococcus faecalis	GH1	4.493392116	1
Streptomyces griseus	GH1	2.688132045	1
Staphylococcus epidermidis	GH1	3.151773558	1
Streptococcus pyogenes	GH1	4.645909728	1
Pantoea ananatis	GH1	3.19668615	1
Escherichia coli	GH1	4.951896641	1
Escherichia coli	GH1	4.951896641	1
Micromonospora aurantiaca	GH1	2.656747513	1
Escherichia coli	GH2	4.951896641	1
Streptosporangium roseum	GH2	2.74523594	1
Stigmatella aurantiaca	GH2	2.897849609	1

Geobacillus sp.	GH2	3.64830473	1
Bacteroides thetaiotaomicron	GH2	5.668359112	1
Pectobacterium atrosepticum	GH2	2.977539914	1
Salinispora tropica	GH1	2.512074083	1
Zunongwangia profunda	GH2	3.493179318	1
Rhizobium leguminosarum	GH2	2.765664497	1
Shewanella loihica	GH2	2.116527093	1
Shigella boydii	GH2	4.467400665	1
Streptomyces ambofaciens	GH2	2.51396916	1
Leuconostoc kimchii	GH2	3.578591417	1
Escherichia coli	GH2	4.951896641	1
Escherichia coli	GH2	4.951896641	1
Flavobacterium johnsoniae	GH2	3.525155341	1
Lachnoclostridium phytofermentans	GH2	4.440620629	1
Staphylococcus aureus	GH1	3.253816682	1
Streptococcus mutans	GH1	4.026249304	1
Mycoplasma leachii	GH1	3.326351905	1
Dickeya zeae	GH1	3.05858332	1
Clostridium acetobutylicum	GH1	1.970128234	0
Streptococcus equi	GH2	3.491210821	1
Deinococcus geothermalis	GH2	2.528732653	1
Butyrivibrio proteoclasticus	GH2	4.213375923	1
Clavibacter michiganensis	GH2	3.019422612	1
Erwinia tasmaniensis	GH2	2.89666889	1
Lactobacillus acidophilus	GH1	4.391043616	1
Mycoplasma mycoides	GH1	3.442192348	1
Vibrio tasmaniensis	GH1	1.139914283	0
Streptococcus pyogenes	GH1	4.645909728	1
Eubacterium eligens	GH1	5.232516296	1
Streptococcus thermophilus	GH2	3.238181275	1
Serratia proteamaculans	GH1	3.231309256	1
Klebsiella pneumoniae	GH1	3.527499461	1
Shigella flexneri	GH2	4.384218202	1
Escherichia coli	GH2	4.951896641	1
Caldicellulosiruptor owensensis	GH2	3.105077595	1
Clavibacter michiganensis	GH2	3.019422612	1
Escherichia coli	GH2	4.951896641	1
Paenibacillus polymyxa	GH1	3.727193955	1
Clostridium saccharolyticum	GH1	5.056490176	1
Lactococcus garvieae	GH1	2.40846932	1

Leuconostoc kimchii	GH1	3.578591417	1
Escherichia coli	GH2	4.951896641	1
Bacillus selenitireducens	GH2	3.381057312	1
Bifidobacterium adolescentis	GH2	5.563837622	1
Bacteroides fragilis	GH2	5.330440553	1
Novosphingobium aromaticivorans	GH2	0.766564697	0
Erwinia billingiae	GH1	3.247037685	1
Bifidobacterium dentium	GH2	4.766725591	1
Clavibacter michiganensis	GH2	3.019422612	1
Escherichia coli	GH1	4.951896641	1
Enterobacter cloacae	GH2	2.178925626	1
Escherichia coli	GH1	4.951896641	1
Opitutus terrae	GH1	2.849000161	1
Bacteroides fragilis	GH2	5.330440553	1
Caldicellulosiruptor kronotskyensis	GH2	3.162669177	1
Lactobacillus amylovorus	GH2	4.651593108	1
Bifidobacterium longum	GH2	5.049759625	1
Bacteroides fragilis	GH2	5.330440553	1
Bacteroides thetaiotaomicron	GH2	5.668359112	1
Maribacter sp.	CBM57	3.442416285	1
Methylorubrum extorquens	CBM57	2.890499779	1
Zunongwangia profunda	GH2	3.493179318	1
Butyrivibrio proteoclasticus	GH2	4.213375923	1
Paenibacillus sp.	GH2	3.716301192	1
Alistipes shahii	GH2	5.507004832	1
Escherichia coli	GH2	4.951896641	1
Bacteroides vulgatus	GH2	5.981194327	1
Escherichia coli	GH2	4.951896641	1
Paenibacillus sp.	GH2	3.716301192	1
Clostridium perfringens	GH2	4.092439715	1
Caldicellulosiruptor hydrothermalis	GH2	3.083906066	1
Streptomyces scabiei	GH2	2.583957419	1
Parabacteroides distasonis	GH2	5.5003263	1
Halanaerobium hydrogeniformans	GH2	3.442305735	1
Escherichia coli	GH2	4.951896641	1
Lactobacillus delbrueckii	GH1	4.330538982	1
Bacillus mycoides	GH1	3.134882408	1
Escherichia fergusonii	GH1	4.301024444	1
Tolumonas auensis	GH2	3.248437991	1
Bacteroides vulgatus	GH2	5.981194327	1

Desulfovibrio magneticus	GH2	3.229710695	1
Listeria monocytogenes	GH1	2.652162063	1
Pediococcus pentosaceus	GH1	3.700450677	1
Streptococcus pyogenes	GH1	4.645909728	1
Kitasatospora setae	GH1	2.897849609	1
Rhodococcus jostii	GH1	2.743611499	1
Lactobacillus plantarum	GH1	3.483288678	1
Thermoanaerobacter mathranii	GH1	3.212248516	1
Dickeya paradisiaca	GH1	3.018719322	1
Bacteroides vulgatus	GH2	5.981194327	1
Paenibacillus sp.	GH2	3.716301192	1
Prevotella ruminicola	GH2	4.464744795	1
Porphyromonas gingivalis	GH2	4.203215583	1
[Ruminococcus] torques	GH2	5.495459591	1
Streptococcus pyogenes	GH2	4.645909728	1
Bacteroides thetaiotaomicron	GH2	5.668359112	1
Saccharopolyspora erythraea	GH2	2.829954331	1
Roseiflexus castenholzii	GH2	1.70877002	1
Streptomyces scabiei	GH2	2.583957419	1
Thermotoga petrophila	GH1	1.906618403	0
Streptococcus pneumoniae	GH1	3.898009613	1
Listeria welshimeri	GH1	3.164987093	1
Roseburia intestinalis	GH2	5.494855325	1
Bacteroides vulgatus	GH2	5.981194327	1
Actinosynnema mirum	GH2	2.698718791	1
Listeria welshimeri	GH1	3.164987093	1
Streptomyces scabiei	GH1	2.583957419	1
Gloeobacter violaceus	GH1	2.991793542	1
Streptococcus dysgalactiae	GH1	3.643525026	1
Thermobifida fusca	GH1	2.489421356	1
Nocardiopsis dassonvillei	GH2	2.731716088	1
Lactobacillus acidophilus	GH2	4.391043616	1
Agathobacter rectalis	GH2	5.641970263	1
Actinosynnema mirum	GH2	2.698718791	1
Paenibacillus sp.	GH2	3.716301192	1
Streptococcus pneumoniae	GH2	3.898009613	1
Bacteroides fragilis	GH2	5.330440553	1
Enterobacter lignolyticus	GH2	3.968988727	1
Bifidobacterium breve	GH1	4.551013712	1
Serratia proteamaculans	GH1	3.231309256	1

Listeria welshimeri	GH1	3.164987093	1
Streptococcus pneumoniae	GH1	3.898009613	1
Koribacter versatilis	GH2	2.871309697	1
Streptococcus pneumoniae	GH2	3.898009613	1
Vibrio campbellii	GH1	2.003060928	1
Flavobacterium johnsoniae	GH2	3.525155341	1
Propionibacterium freudenreichii	GH2	3.746868029	1
Klebsiella pneumoniae	GH2	3.527499461	1
Dickeya paradisiaca	GH2	3.018719322	1
Cyanothece sp.	GH2	2.72078854	1
Streptococcus pyogenes	GH2	4.645909728	1
Parabacteroides distasonis	GH2	5.5003263	1
Bacteroides fragilis	GH2	5.330440553	1
Deinococcus deserti	GH2	2.963585295	1
Bacteroides xylanisolvens	GH2	5.755848669	1
Roseiflexus castenholzii	GH2	1.70877002	1
Solibacter usitatus	GH2	3.694382176	1
Bacteroides fragilis	GH2	5.330440553	1
Teredinibacter turnerae	GH30	2.455699954	1
Methylorubrum extorquens	CBM57	2.890499779	1
Agrobacterium vitis	GH1	2.861500523	1
Streptococcus dysgalactiae	GH2	3.643525026	1
Prevotella melaninogenica	GH2	4.645622238	1
Azospirillum sp.	GH1	2.549653156	1
Thermobispora bispora	GH1	2.603002819	1
Clavibacter michiganensis	GH1	3.019422612	1
Staphylococcus aureus	GH1	3.253816682	1
Bacillus clausii	GH1	3.122214447	1
Shigella flexneri	GH1	4.384218202	1
Lactobacillus sakei	GH1	3.906365165	1
Escherichia coli	GH1	4.951896641	1
Clostridium saccharolyticum	GH2	5.056490176	1
Bacteroides thetaiotaomicron	GH2	5.668359112	1
Lactobacillus acidophilus	GH1	4.391043616	1
Pectobacterium carotovorum	GH1	3.146442611	1
Escherichia coli	GH1	4.951896641	1
Paenibacillus polymyxa	GH1	3.727193955	1
Streptococcus pyogenes	GH1	4.645909728	1
Dictyoglomus thermophilum	GH2	2.597989089	1
Geobacillus sp.	GH2	3.64830473	1

Streptomyces bingchenggensis	GH2	2.546261927	1
Escherichia coli	GH2	4.951896641	1
Lactococcus lactis	GH1	4.535318878	1
Streptomyces bingchenggensis	GH1	2.546261927	1
Listeria innocua	GH1	2.642937634	1
Clostridium saccharolyticum	GH1	5.056490176	1
Caulobacter vibrioides	GH1	1.916277954	1
Escherichia coli	GH2	4.951896641	1
Bacteroides vulgatus	GH2	5.981194327	1
Streptosporangium roseum	GH1	2.74523594	1
Haemophilus somnus	GH2	3.17727404	1
Flavobacterium johnsoniae	GH2	3.525155341	1
Deinococcus deserti	GH2	2.963585295	1
Oenococcus oeni	GH1	3.610261575	1
Bacteroides xylanisolvens	GH2	5.755848669	1
Escherichia coli	GH2	4.951896641	1
Geobacillus sp.	GH2	3.64830473	1
Eubacterium eligens	GH2	5.232516296	1
Dyadobacter fermentans	GH30	3.417983563	1
Photorhabdus laumondii	GH30	2.248422138	1
Halanaerobium praevalens	GH2	3.109148225	1
Koribacter versatilis	GH30	2.871309697	1
Enterococcus faecalis	GH1	4.493392116	1
Clostridium saccharolyticum	GH1	5.056490176	1
Escherichia coli	GH1	4.951896641	1
Sebaldella termitidis	GH1	2.899978786	1
Caldicellulosiruptor owensensis	GH1	3.105077595	1
Frankia alni	GH30	2.650947694	1
Rhodoferax ferrireducens	GH30	2.976100809	1
Bacteroides thetaiotaomicron	GH30	5.668359112	1
Paludibacter propionicigenes	GH30	3.910160743	1
Escherichia coli	GH2	4.951896641	1
Escherichia coli	GH2	4.951896641	1
Streptomyces sp.	GH2	0.351839024	0
Salmonella paratyphi	GH1	3.414481535	1
Clostridioides difficile	GH1	5.039965306	1
Butyrivibrio proteoclasticus	GH30	4.213375923	1
Bifidobacterium breve	GH30	4.551013712	1
Zunongwangia profunda	GH30	3.493179318	1
Agathobacter rectalis	GH1	5.641970263	1

Methylacidiphilum infernorum	GH1	1.894736198	0
Clostridium beijerinckii	GH1	4.172192327	1
Photorhabdus laumondii	GH1	2.248422138	1
Parabacteroides distasonis	GH30	5.5003263	1
Sanguibacter keddieii	GH30	2.855701308	1
Paludibacter propionicigenes	GH30	3.910160743	1
Bacillus pumilus	GH30	3.248670682	1
Chitinophaga pinensis	GH30	3.132181861	1
Spirosoma linguale	GH30	3.535049207	1
Escherichia coli	GH2	4.951896641	1
Zunongwangia profunda	GH2	3.493179318	1
Roseiflexus sp.	GH2	2.092405838	1
Rhodothermus marinus	GH2	2.502529311	1
Erwinia billingiae	GH2	3.247037685	1
Solibacter usitatus	CBM57	3.694382176	1
Coraliomargarita akajimensis	CBM57	3.018355261	1
Escherichia coli	GH2	4.951896641	1
Streptomyces scabiei	GH2	2.583957419	1
Dictyoglomus turgidum	GH2	2.698198103	1
Frankia sp.	GH2	2.890273357	1
Stigmatella aurantiaca	GH30	2.897849609	1
Novosphingobium aromaticivorans	GH79	0.766564697	0
Catenulispora acidiphila	GH79	2.886534124	1
Brachybacterium faecium	GH79	2.322733424	1
Burkholderia pseudomallei	GH79	3.93219767	1
Sediminispirochaeta smaragdinae	GH79	4.100615895	1
Solibacter usitatus	GH79	3.694382176	1
Teredinibacter turnerae	GH79	2.455699954	1
Opitutus terrae	GH2	2.849000161	1
Methylovorus glucosetrophus	GH2	3.197224485	1
Pedobacter heparinus	GH2	3.537943992	1
Zunongwangia profunda	GH2	3.493179318	1
Escherichia coli	GH2	4.951896641	1
Burkholderia pseudomallei	GH79	3.93219767	1
Lachnoclostridium phytofermentans	GH2	4.440620629	1
Shigella boydii	GH2	4.467400665	1
Anaerococcus prevotii	GH2	3.578456843	1
Escherichia coli	GH2	4.951896641	1
Acaryochloris marina	GH1	2.383863923	1
Agrobacterium fabrum	GH2	2.626901456	1

Frankia sp.	GH2	2.890273357	1
Caldicellulosiruptor saccharolyticus	GH2	3.082502309	1
Ruminococcus champanellensis	GH2	5.00629656	1
Streptomyces scabiei	GH1	2.583957419	1
Xanthomonas campestris	GH2	2.836321535	1
Staphylococcus aureus	GH1	3.253816682	1
Thermobispora bispora	GH1	2.603002819	1
Nakamurella multipartita	GH1	2.617574677	1
Cronobacter sakazakii	GH2	3.663917102	1
Streptococcus uberis	GH2	4.209153639	1
Bifidobacterium longum	GH2	5.049759625	1
Sanguibacter keddieii	GH2	2.855701308	1
Streptococcus pyogenes	GH1	4.645909728	1
Actinosynnema mirum	GH1	2.698718791	1
Bradyrhizobium diazoefficiens	GH1	3.166834227	1
Butyrivibrio proteoclasticus	GH2	4.213375923	1
Brachybacterium faecium	GH2	2.322733424	1
Bacteroides fragilis	GH2	5.330440553	1
Dictyoglomus turgidum	GH2	2.698198103	1
Escherichia coli	GH2	4.951896641	1
Bacillus licheniformis	GH1	2.921693481	1
Streptococcus thermophilus	GH2	3.238181275	1
Sorangium cellulosum	GH2	2.502583787	1
Lactobacillus brevis	GH1	3.475230992	1
Escherichia fergusonii	GH1	4.301024444	1
Aeromonas salmonicida	GH1	2.970119225	1
Streptomyces scabiei	GH1	2.583957419	1
Escherichia coli	GH2	4.951896641	1
Frankia sp.	GH2	2.890273357	1
Rhizobium leguminosarum	GH2	2.765664497	1
Roseburia intestinalis	GH2	5.494855325	1
Geobacillus sp.	GH2	3.64830473	1
Spirosoma linguale	GH1	3.535049207	1
Clostridioides difficile	GH1	5.039965306	1
Shigella dysenteriae	GH2	4.389565899	1
Ruminiclostridium cellulolyticum	GH2	4.261509628	1
Bifidobacterium longum	GH2	5.049759625	1
Flavobacterium johnsoniae	GH2	3.525155341	1
Solibacter usitatus	GH2	3.694382176	1
Chloroflexus aggregans	GH1	1.316259304	1

Thermotoga naphthophila	GH1	2.301883351	1
Rhodobacter sphaeroides	GH1	2.423885576	1
Lactococcus lactis	GH1	4.535318878	1
Acidobacterium capsulatum	GH2	2.325925309	1
Citrobacter koseri	GH2	4.495223425	1
Gramella forsetii	GH2	3.311848451	1
Streptomyces fungicidicus	GH2	2.44014973	1
Escherichia coli	GH2	4.951896641	1
Streptomyces griseus	GH1	2.688132045	1
Streptococcus pyogenes	GH1	4.645909728	1
Geobacter bemidjiensis	GH1	2.993336071	1
Streptomyces scabiei	GH2	2.583957419	1
Rhodopseudomonas palustris	GH1	2.986919212	1
Agrobacterium fabrum	GH1	2.626901456	1
Bacteroides thetaiotaomicron	GH2	5.668359112	1
Escherichia coli	GH2	4.951896641	1
Bifidobacterium dentium	GH1	4.766725591	1
Streptococcus pyogenes	GH1	4.645909728	1
Shigella flexneri	GH1	4.384218202	1
Streptococcus pyogenes	GH1	4.645909728	1
Lactobacillus sakei	GH1	3.906365165	1
Stackebrandtia nassauensis	GH1	2.705407885	1
Sebaldella termitidis	GH1	2.899978786	1
Ruminiclostridium cellulolyticum	GH30	4.261509628	1
Bacillus licheniformis	GH1	2.921693481	1
Caldicellulosiruptor bescii	GH1	3.125737391	1
Nakamurella multipartita	GH1	2.617574677	1
Tolumonas auensis	GH1	3.248437991	1
Kitasatospora setae	GH1	2.897849609	1
Streptococcus uberis	GH1	4.209153639	1
Chloroflexus aurantiacus	GH1	1.343763416	1
Lachnoclostridium phytofermentans	GH1	4.440620629	1
Bacteroides xylanisolvens	GH30	5.755848669	1
Conexibacter woesei	GH30	2.992179949	1
Catenulispora acidiphila	GH30	2.886534124	1
Haliangium ochraceum	GH30	2.906275029	1
Sanguibacter keddieii	GH30	2.855701308	1
Butyrivibrio proteoclasticus	GH2	4.213375923	1
Saccharophagus degradans	GH2	2.105502422	0
Escherichia coli	GH2	4.951896641	1

Acholeplasma laidlawii	GH30	3.521063112	1
Bacteroides thetaiotaomicron	GH30	5.668359112	1
Lachnoclostridium phytofermentans	GH30	4.440620629	1
Bifidobacterium animalis	GH30	4.326031942	1
Leptotrichia buccalis	GH1	3.234674622	1
Exiguobacterium sp.	GH1	3.604041401	1
Shewanella amazonensis	GH1	2.21211605	1
Faecalibacterium prausnitzii	GH1	5.703104557	1
Streptococcus pneumoniae	GH1	3.898009613	1
Streptomyces coelicolor	GH2	1.758481979	1
Lactobacillus acidophilus	GH2	4.391043616	1
Fibrobacter succinogenes	GH2	3.403591123	1
Salmonella gallinarum	GH1	3.384673024	1
Thermoanaerobacter pseudethanolicus	GH1	3.370655894	1
Salmonella agona	GH1	3.588748032	1
Tolumonas auensis	GH1	3.248437991	1
Streptococcus pneumoniae	GH1	3.898009613	1
Methylorubrum extorquens	CBM57	2.890499779	1
Bacteroides xylanisolvens	CBM57	5.755848669	1
Leadbetterella byssophila	CBM57	3.698360007	1
Micromonospora aurantiaca	CBM57	2.656747513	1
Burkholderia pseudomallei	GH79	3.93219767	1
Clostridium beijerinckii	GH2	4.172192327	1
Vibrio cholerae	GH2	2.401983537	1
Burkholderia sp.	GH2	3.185120973	1
Agrobacterium radiobacter	GH2	2.773077398	1
Kineococcus radiotolerans	GH2	3.03645742	1
Bacteroides thetaiotaomicron	GH2	5.668359112	1
Shigella sp.	GH2	1.565835599	1
Frankia inefficax	GH2	2.921941994	1
Sanguibacter keddieii	GH2	2.855701308	1
Geobacillus sp.	GH2	3.64830473	1
Streptococcus sanguinis	GH1	4.260660059	1
Clostridium botulinum	GH1	4.053974807	1
Streptomyces coelicolor	GH2	1.758481979	1
[Ruminococcus] torques	GH2	5.495459591	1
Bacteroides fragilis	GH2	5.330440553	1
Dyadobacter fermentans	GH2	3.417983563	1
Brevundimonas subvibrioides	GH2	2.525486274	1
Gramella forsetii	GH2	3.311848451	1

Xanthomonas axonopodis	GH2	1.902261106	1
Xanthomonas axonopodis	GH2	1.902261106	1
Nocardioides sp.	GH1	2.528732653	1
Clostridioides difficile	GH1	5.039965306	1
Bacillus subtilis	GH1	3.020649231	1
Burkholderia ambifaria	GH1	2.822519768	1
Escherichia coli	GH2	4.951896641	1
Butyrivibrio fibrisolvens	GH2	4.079675342	1
Vibrio vulnificus	GH2	1.625869887	0
Pectobacterium carotovorum	GH1	3.146442611	1
Dickeya dadantii	GH1	3.173053214	1
Thermotoga petrophila	GH2	1.906618403	0
Clostridium acetobutylicum	GH1	1.970128234	0
Frankia sp.	GH1	2.890273357	1
Bacteroides fragilis	GH2	5.330440553	1
Spirosoma linguale	GH2	3.535049207	1
Listeria welshimeri	GH1	3.164987093	1
Streptococcus pyogenes	GH1	4.645909728	1
Micromonospora aurantiaca	GH1	2.656747513	1
Meiothermus ruber	GH1	2.375226576	1
Streptococcus dysgalactiae	GH2	3.643525026	1
Streptococcus thermophilus	GH2	3.238181275	1
Bifidobacterium bifidum	GH2	5.013833965	1
Shigella boydii	GH2	4.467400665	1
Escherichia coli	GH2	4.951896641	1
Bacillus velezensis	GH1	3.007591831	1
Escherichia coli	GH1	4.951896641	1
Rhodopseudomonas palustris	GH1	2.986919212	1
Dyadobacter fermentans	GH1	3.417983563	1
Hahella chejuensis	GH1	2.204844609	0
Escherichia coli	GH2	4.951896641	1
Geobacillus sp.	GH2	3.64830473	1
Rhizobium leguminosarum	GH2	2.765664497	1
Bacteroides thetaiotaomicron	GH2	5.668359112	1
Coraliomargarita akajimensis	GH2	3.018355261	1
Clostridium saccharolyticum	GH2	5.056490176	1
Cupriavidus necator	GH2	2.920028476	1
Streptococcus thermophilus	GH2	3.238181275	1
Lactobacillus plantarum	GH2	3.483288678	1
Escherichia coli	GH2	4.951896641	1

Novosphingobium aromaticivorans	GH2	0.766564697	0
Escherichia coli	GH2	4.951896641	1
Faecalibacterium prausnitzii	GH2	5.703104557	1
Thermotoga naphthophila	GH2	2.301883351	1
Escherichia coli	GH2	4.951896641	1
Xanthomonas campestris	GH2	2.836321535	1
Klebsiella pneumoniae	GH2	3.527499461	1
Lactobacillus delbrueckii	GH2	4.330538982	1
Faecalibacterium prausnitzii	GH2	5.703104557	1
Streptococcus equi	GH2	3.491210821	1
Burkholderia pseudomallei	GH2	3.93219767	1
Cupriavidus pinatubonensis	GH2	3.164662741	1
Escherichia coli	GH2	4.951896641	1
Caldicellulosiruptor saccharolyticus	GH2	3.082502309	1
Caldanaerobacter subterraneus	GH1	3.090958877	1
Rhodobacter sphaeroides	GH1	2.423885576	1
Bacteroides thetaiotaomicron	GH2	5.668359112	1
Clostridium beijerinckii	GH1	4.172192327	1
Shewanella baltica	GH1	2.273258695	1
Streptomyces scabiei	GH1	2.583957419	1
Spirochaeta thermophila	GH1	3.412755316	1
Clostridium botulinum	GH1	4.053974807	1
Bradyrhizobium diazoefficiens	GH1	3.166834227	1
Lactococcus lactis	GH1	4.535318878	1
Enterobacter sp.	GH1	4.156645836	1
Bacillus subtilis	GH30	3.020649231	1
Lachnoclostridium phytofermentans	GH30	4.440620629	1
Catenulispora acidiphila	GH30	2.886534124	1
Saccharophagus degradans	GH30	2.105502422	0
Ruminococcus champanellensis	GH30	5.00629656	1
Edwardsiella tarda	GH1	3.149009708	1
Sanguibacter keddieii	GH1	2.855701308	1
Salinispora tropica	GH1	2.512074083	1
Klebsiella pneumoniae	GH1	3.527499461	1
Nakamurella multipartita	GH1	2.617574677	1
Enterobacter sp.	GH1	4.156645836	1
Sanguibacter keddieii	GH1	2.855701308	1
Bacteroides fragilis	GH2	5.330440553	1
Bacillus subtilis	GH1	3.020649231	1
Dickeya dadantii	GH1	3.173053214	1

Rhizobium meliloti	GH1	2.887519452	1
Bacteroides fragilis	GH2	5.330440553	1
Jonesia denitrificans	GH1	2.93633819	1
Shewanella denitrificans	GH1	2.060153867	0
Enterobacter sp.	GH1	4.156645836	1
Streptococcus dysgalactiae	GH1	3.643525026	1
Salinispora arenicola	GH1	2.461696715	1
Lactobacillus rhamnosus	GH1	0.908508323	0
Sphingopyxis alaskensis	GH2	2.501442593	1
Lactobacillus crispatus	GH1	3.911834731	1
Curvibacter putative	GH1	1.139914283	0
Salmonella choleraesuis	GH1	3.399683172	1
Lactococcus lactis	GH1	4.535318878	1
Enterobacter sp.	GH1	4.156645836	1
Clavibacter michiganensis	GH1	3.019422612	1
Escherichia coli	GH1	4.951896641	1
Meiothermus ruber	GH1	2.375226576	1
Lactobacillus plantarum	GH1	3.483288678	1
Pectobacterium carotovorum	GH1	3.146442611	1
Arthrobacter sp.	GH1	2.788922725	1
Herpetosiphon aurantiacus	GH1	2.467219737	1
Paenibacillus polymyxa	GH1	3.727193955	1
Rhizobium etli	GH2	2.801391781	1
Beutenbergia cavernae	GH2	2.742712219	1
Kocuria rhizophila	GH2	2.829455706	1
Yersinia pestis	GH2	3.433167805	1
Listeria welshimeri	GH1	3.164987093	1
Streptococcus suis	GH1	4.75869111	1
Lactococcus lactis	GH1	4.535318878	1
Chloroherpeton thalassium	GH2	1.730469282	0
Roseburia intestinalis	GH2	5.494855325	1
Chitinophaga pinensis	GH2	3.132181861	1
Escherichia coli	GH2	4.951896641	1
Roseburia intestinalis	GH2	5.494855325	1
Ruminococcus champanellensis	GH1	5.00629656	1
Listeria welshimeri	GH1	3.164987093	1
Rhodobacter sphaeroides	GH1	2.423885576	1
Clostridium beijerinckii	GH1	4.172192327	1
Lactococcus lactis	GH1	4.535318878	1
Streptococcus pneumoniae	GH1	3.898009613	1

Streptococcus suis	GH1	4.75869111	1
Tolumonas auensis	GH1	3.248437991	1
Pseudarthrobacter chlorophenolicus	GH1	2.741360004	1
Mesoplasma florum	GH1	3.526749672	1
Gloeobacter violaceus	GH1	2.991793542	1
Sorangium cellulosum	GH1	2.502583787	1
Desulfotalea psychrophila	GH1	3.198273043	1
Rhodoferax ferrireducens	GH1	2.976100809	1
Lactobacillus paracasei	GH1	4.039963241	1
Klebsiella pneumoniae	GH1	3.527499461	1
Streptomyces scabiei	GH2	2.583957419	1
Streptococcus pneumoniae	GH1	3.898009613	1
Colwellia psychrerythraea	GH1	2.109088489	1
Erwinia amylovora	GH1	2.90156558	1
Clostridium botulinum	GH1	4.053974807	1
Escherichia coli	GH1	4.951896641	1
Clostridium beijerinckii	GH1	4.172192327	1
Caldicellulosiruptor obsidiansis	GH2	3.232298508	1
Thermoanaerobacter mathranii	GH2	3.212248516	1
Stigmatella aurantiaca	GH2	2.897849609	1
Bacteroides vulgatus	GH2	5.981194327	1
Kribbella flavida	GH1	2.574757512	1
Escherichia coli	GH2	4.951896641	1
Kribbella flavida	GH1	2.574757512	1
Cellulomonas flavigena	GH1	2.903929771	1
Clostridium longisporum	GH1	1.040144071	0
Actinosynnema mirum	GH2	2.698718791	1
Escherichia coli	GH2	4.951896641	1
Escherichia coli	GH2	4.951896641	1
Shigella boydii	GH2	4.467400665	1
Streptomyces griseus	GH2	2.688132045	1
Bacteroides fragilis	GH2	5.330440553	1
Atopobium parvulum	GH2	3.852930421	1
Leadbetterella byssophila	GH2	3.698360007	1
Bifidobacterium longum	GH2	5.049759625	1
Bacteroides xylanisolvens	GH2	5.755848669	1
Lactobacillus helveticus	GH2	4.191208361	1
Shigella flexneri	GH2	4.384218202	1
Klebsiella pneumoniae	GH1	3.527499461	1
Escherichia coli	GH1	4.951896641	1

Jannaschia sp.	GH1	2.46058051	1
Lactococcus garvieae	GH1	2.40846932	1
Clostridioides difficile	GH1	5.039965306	1
Dickeya chrysanthemi	GH1	2.985934376	1
Saccharophagus degradans	GH2	2.105502422	0
Chloroflexus aurantiacus	GH1	1.343763416	1
Lactococcus lactis	GH1	4.535318878	1
Enterobacter lignolyticus	GH1	3.968988727	1
Streptococcus uberis	GH1	4.209153639	1
Serratia proteamaculans	GH1	3.231309256	1
Dictyoglomus thermophilum	GH1	2.597989089	1
Clostridium botulinum	GH1	4.053974807	1
Bifidobacterium bifidum	GH2	5.013833965	1
Shigella boydii	GH1	4.467400665	1
Escherichia coli	GH1	4.951896641	1
Leuconostoc mesenteroides	GH1	4.403938925	1
Escherichia coli	GH1	4.951896641	1
Lactobacillus acidophilus	GH1	4.391043616	1
Frankia inefficax	GH1	2.921941994	1
Lactobacillus plantarum	GH1	3.483288678	1
Streptococcus pneumoniae	GH1	3.898009613	1
Listeria monocytogenes	GH1	2.652162063	1
Zunongwangia profunda	GH1	3.493179318	1
Enterobacter sp.	GH1	4.156645836	1
Streptomyces ambofaciens	GH1	2.51396916	1
Clostridium acetobutylicum	GH1	1.970128234	0
Stigmatella aurantiaca	GH1	2.897849609	1
Thermomicrobium roseum	GH1	2.028694511	0
Bacillus halodurans	GH1	3.382316202	1
Aliivibrio fischeri	GH1	2.148976013	1
Clostridioides difficile	GH1	5.039965306	1
Phenylobacterium zucineum	GH1	2.898116603	1
Maribacter sp.	GH2	3.442416285	1
Xanthomonas campestris	GH2	2.836321535	1
Lactobacillus crispatus	GH1	3.911834731	1
Cronobacter sakazakii	GH1	3.663917102	1
Magnetospirillum magneticum	GH1	2.983954556	1
Exiguobacterium sp.	GH1	3.604041401	1
Paenibacillus polymyxa	GH1	3.727193955	1
Atopobium parvulum	GH1	3.852930421	1

Yersinia enterocolitica	GH1	3.206616339	1
Mycoplasma fermentans	GH1	2.655176326	1
Bacillus licheniformis	GH1	2.921693481	1
Kribbella flavida	GH1	2.574757512	1
Sebaldella termitidis	GH1	2.899978786	1
Vibrio campbellii	GH1	2.003060928	1
Shigella boydii	GH2	4.467400665	1
Bacteroides ovatus	GH2	2.5496461	1
[Eubacterium] siraeum	GH2	5.238059667	1
Pedobacter heparinus	GH2	3.537943992	1
Streptomyces bingchenggensis	GH2	2.546261927	1
Shigella sonnei	GH2	4.50932433	1
Thermotoga neapolitana	GH2	2.194896294	1
Streptococcus thermophilus	GH2	3.238181275	1
Escherichia coli	GH2	4.951896641	1
Rhizobium leguminosarum	GH2	2.765664497	1
Thermotoga petrophila	GH2	1.906618403	0
Bacteroides fragilis	GH2	5.330440553	1
Bifidobacterium bifidum	GH2	5.013833965	1
Lactobacillus paracasei	GH1	4.039963241	1
Sorangium cellulosum	GH1	2.502583787	1
Frankia sp.	GH2	2.890273357	1
Bifidobacterium dentium	GH2	4.766725591	1
Saccharophagus degradans	GH2	2.105502422	0
Paenibacillus sp.	GH2	3.716301192	1
Bacteroides thetaiotaomicron	GH2	5.668359112	1
Treponema denticola	GH2	4.109719061	1
Clostridium saccharolyticum	GH2	5.056490176	1
Rhizobium etli	GH2	2.801391781	1
[Eubacterium] siraeum	GH2	5.238059667	1
Bacteroides fragilis	GH2	5.330440553	1
Escherichia coli	GH2	4.951896641	1
Zunongwangia profunda	GH2	3.493179318	1
Escherichia coli	GH1	4.951896641	1
Clostridium acetobutylicum	GH1	1.970128234	0
Streptococcus pneumoniae	GH1	3.898009613	1
Citrobacter rodentium	GH1	4.007244277	1
Escherichia coli	GH2	4.951896641	1
Prevotella ruminicola	GH2	4.464744795	1
Streptococcus pneumoniae	GH1	3.898009613	1

Shigella sonnei	GH1	4.50932433	1
Bacillus velezensis	GH1	3.007591831	1
Lactobacillus gasseri	GH1	2.459107723	1
Shewanella violacea	GH2	2.214505056	1
Rhizobium leguminosarum	GH2	2.765664497	1
Escherichia coli	GH2	4.951896641	1
Clavibacter michiganensis	GH2	3.019422612	1
Rhizobium meliloti	GH2	2.887519452	1
Escherichia coli	GH2	4.951896641	1
Rothia mucilaginosa	GH1	4.342204271	1
Thermoanaerobacter pseudethanolicus	GH1	3.370655894	1
Geobacillus thermodenitrificans	GH2	3.274686288	1
Yersinia pseudotuberculosis	GH2	3.271820733	1
Streptococcus equi	GH1	3.491210821	1
Vibrio antiquarius	GH1	1.642331507	1
Nakamurella multipartita	GH1	2.617574677	1
Lactobacillus amylovorus	GH1	4.651593108	1
Chitinophaga pinensis	GH2	3.132181861	1
Erwinia pyrifoliae	GH1	2.917976808	1
Klebsiella oxytoca	GH1	2.715911685	1
Citrobacter koseri	GH1	4.495223425	1
Clostridium cellulovorans	GH1	4.295967255	1
Escherichia coli	GH2	4.951896641	1
Shigella flexneri	GH1	4.384218202	1
Caldicellulosiruptor kristjanssonii	GH2	3.125858087	1
Paenibacillus polymyxa	GH2	3.727193955	1
Escherichia coli	GH2	4.951896641	1
Aeromonas hydrophila	GH2	3.103122052	1
Pseudothermotoga lettingae	GH2	2.003060928	1
Actinosynnema mirum	GH1	2.698718791	1
Staphylococcus aureus	GH1	3.253816682	1
Pectobacterium carotovorum	GH1	3.146442611	1
Alicyclobacillus acidocaldarius	GH1	3.380511029	1
Thermotoga neapolitana	GH1	2.194896294	1
Shewanella baltica	GH1	2.273258695	1
Clavibacter michiganensis	GH1	3.019422612	1
Caldicellulosiruptor obsidiansis	GH2	3.232298508	1
Rhizobium leguminosarum	GH2	2.765664497	1
Streptococcus pneumoniae	GH1	3.898009613	1
Mycolicibacterium smegmatis	GH1	2.248422138	1

Streptococcus pneumoniae	GH1	3.898009613	1
Dictyoglomus turgidum	GH1	2.698198103	1
Clostridium botulinum	GH1	4.053974807	1
Bacillus subtilis	GH1	3.020649231	1
Leuconostoc mesenteroides	GH1	4.403938925	1
Deinococcus geothermalis	GH1	2.528732653	1
Rhizobium etli	GH2	2.801391781	1
Lactococcus lactis	GH1	4.535318878	1
Corynebacterium kroppenstedtii	GH1	2.748506441	1
Bacillus pumilus	GH1	3.248670682	1
Pectobacterium carotovorum	GH1	3.146442611	1
Vibrio tasmaniensis	GH1	1.139914283	0
[Ruminococcus] torques	GH1	5.495459591	1
Aliivibrio salmonicida	GH1	2.324818688	1
Zunongwangia profunda	GH30	3.493179318	1
Clostridioides difficile	GH1	5.039965306	1
Mycolicibacterium smegmatis	GH1	2.248422138	1
Roseobacter denitrificans	GH1	2.542833577	1
Klebsiella pneumoniae	GH1	3.527499461	1
Escherichia coli	GH1	4.951896641	1
Streptococcus pyogenes	GH1	4.645909728	1
Cronobacter turicensis	GH1	1.75620155	0
Caldicellulosiruptor hydrothermalis	GH30	3.083906066	1
Salmonella agona	GH30	3.588748032	1
Acidimicrobium ferrooxidans	GH30	2.557947421	1
Streptosporangium roseum	GH2	2.74523594	1
Faecalibacterium prausnitzii	GH2	5.703104557	1
Lactobacillus crispatus	GH1	3.911834731	1
Clostridioides difficile	GH1	5.039965306	1
Cronobacter sakazakii	GH1	3.663917102	1
Sorangium cellulosum	GH1	2.502583787	1
Clostridium beijerinckii	GH1	4.172192327	1
uncultured bacterium	GH1	4.006358463	1
Streptococcus pneumoniae	GH1	3.898009613	1
Kitasatospora setae	GH1	2.897849609	1
Escherichia coli	GH2	4.951896641	1
Shewanella piezotolerans	GH2	2.071245846	1
Thermosipho africanus	GH2	2.118718832	0
Bacteroides fragilis	GH2	5.330440553	1
Faecalibacterium prausnitzii	GH2	5.703104557	1

Shigella boydii	GH2	4.467400665	1
Citrobacter freundii	GH2	2.755642327	1
Lactococcus lactis	GH2	4.535318878	1
Paenibacillus polymyxa	GH1	3.727193955	1
Streptomyces ambofaciens	GH1	2.51396916	1
Enterococcus faecalis	GH1	4.493392116	1
Streptomyces scabiei	GH2	2.583957419	1
Blautia obeum	GH2	5.41829818	1
Shewanella halifaxensis	GH2	1.93344511	0
Escherichia coli	GH2	4.951896641	1
Bacteroides fragilis	GH2	5.330440553	1
Eubacterium eligens	GH2	5.232516296	1
Lactobacillus plantarum	GH2	3.483288678	1
Streptococcus thermophilus	GH2	3.238181275	1
Shigella sonnei	GH2	4.50932433	1
Escherichia coli	GH2	4.951896641	1
Bacteroides xylanisolvens	CBM57	5.755848669	1
Methylorubrum extorquens	CBM57	2.890499779	1
Bacteroides xylanisolvens	CBM57	5.755848669	1
Methylorubrum populi	CBM57	2.938409167	1
Zunongwangia profunda	GH2	3.493179318	1
Spirosoma linguale	GH2	3.535049207	1
Streptomyces bingchenggensis	GH2	2.546261927	1
Parabacteroides distasonis	GH2	5.5003263	1
Streptococcus uberis	GH79	4.209153639	1
Streptococcus pyogenes	GH1	4.645909728	1
Streptococcus uberis	GH1	4.209153639	1
Bacillus velezensis	GH1	3.007591831	1
Lactobacillus johnsonii	GH1	3.821988045	1
Rhodopseudomonas palustris	GH1	2.986919212	1
Prevotella ruminicola	GH2	4.464744795	1
Paenibacillus sp.	GH2	3.716301192	1
Burkholderia lata	GH2	2.633788296	1
Thermotoga neapolitana	GH2	2.194896294	1
Bacteroides thetaiotaomicron	GH2	5.668359112	1
Rhodococcus jostii	GH2	2.743611499	1
Klebsiella pneumoniae	GH2	3.527499461	1
Streptococcus thermophilus	GH2	3.238181275	1
Halothermothrix orenii	GH2	3.329596854	1
Porphyromonas gingivalis	GH2	4.203215583	1

Streptococcus pneumoniae	GH2	3.898009613	1
Prevotella melaninogenica	GH2	4.645622238	1
Shigella boydii	GH2	4.467400665	1
Catenulispora acidiphila	GH2	2.886534124	1
Rhizobium leguminosarum	GH2	2.765664497	1
Thermotoga naphthophila	GH2	2.301883351	1
Porphyromonas gingivalis	GH2	4.203215583	1
Rhizobium etli	GH2	2.801391781	1
Bacteroides fragilis	GH2	5.330440553	1
Citrobacter koseri	GH2	4.495223425	1
Faecalibacterium prausnitzii	GH2	5.703104557	1
Parabacteroides distasonis	GH2	5.5003263	1
Pseudoalteromonas atlantica	GH2	2.341949533	1
Listeria monocytogenes	GH1	2.652162063	1
Mesoplasma florum	GH1	3.526749672	1
Ruminococcus sp.	GH1	5.524674293	1
Lactococcus lactis	GH1	4.535318878	1
Escherichia coli	GH2	4.951896641	1
Prevotella ruminicola	GH2	4.464744795	1
Parabacteroides distasonis	GH2	5.5003263	1
Vibrio antiquarius	GH2	1.642331507	1
Streptococcus pneumoniae	GH1	3.898009613	1
Listeria monocytogenes	GH1	2.652162063	1
Staphylococcus haemolyticus	GH1	3.184925706	1
Burkholderia cenocepacia	GH2	2.663298853	1
Listeria welshimeri	GH1	3.164987093	1
Oceanobacillus iheyensis	GH1	3.188323218	1
Escherichia coli	GH2	4.951896641	1
Alistipes shahii	GH2	5.507004832	1
Bifidobacterium dentium	GH2	4.766725591	1
Coprococcus catus	GH2	5.204157232	1
Xylella fastidiosa	GH2	2.313735638	1
Beutenbergia cavernae	GH2	2.742712219	1
Escherichia coli	GH2	4.951896641	1
Escherichia coli	GH2	4.951896641	1
Clostridium perfringens	GH2	4.092439715	1
Dickeya paradisiaca	GH1	3.018719322	1
Streptosporangium roseum	GH1	2.74523594	1
Streptococcus pneumoniae	GH1	3.898009613	1
Micromonospora aurantiaca	GH1	2.656747513	1

Clostridium botulinum	GH30	4.053974807	1
Flavobacterium johnsoniae	GH30	3.525155341	1
Chitinophaga pinensis	GH30	3.132181861	1
Streptomyces bingchenggensis	GH30	2.546261927	1
Flavobacterium johnsoniae	GH30	3.525155341	1
Streptomyces ambofaciens	GH30	2.51396916	1
Bifidobacterium animalis	GH30	4.326031942	1
Bacteroides vulgatus	GH30	5.981194327	1
Hungateiclostridium thermocellum	GH30	4.161244363	1
Butyrivibrio proteoclasticus	GH30	4.213375923	1
Streptomyces bingchenggensis	GH30	2.546261927	1
Streptomyces scabiei	GH30	2.583957419	1
Solibacter usitatus	GH30	3.694382176	1
Pseudarthrobacter chlorophenolicus	GH30	2.741360004	1
Flavobacterium johnsoniae	GH30	3.525155341	1
Cutibacterium acnes	GH1	3.525541687	1
Geobacillus thermodenitrificans	GH1	3.274686288	1
Lactobacillus crispatus	GH1	3.911834731	1
Bacteroides fragilis	GH30	5.330440553	1
Exiguobacterium sibiricum	GH1	3.677138081	1
Pseudarthrobacter chlorophenolicus	GH2	2.741360004	1
Listeria monocytogenes	GH1	2.652162063	1
Xanthomonas campestris	GH30	2.836321535	1
Bacillus subtilis	GH30	3.020649231	1
Bacteroides vulgatus	GH30	5.981194327	1
Dickeya zeae	GH30	3.05858332	1
Catenulispora acidiphila	GH30	2.886534124	1
Parabacteroides distasonis	GH30	5.5003263	1
Cutibacterium acnes	GH2	3.525541687	1
Escherichia coli	GH2	4.951896641	1
Escherichia coli	GH2	4.951896641	1
Solibacter usitatus	GH2	3.694382176	1
Escherichia coli	GH2	4.951896641	1
Capnocytophaga ochracea	GH2	3.168311961	1
Enterobacter cloacae	GH2	2.178925626	1
Pantoea vagans	GH2	3.252520094	1
Escherichia coli	GH2	4.951896641	1
Bacteroides thetaiotaomicron	GH2	5.668359112	1
Escherichia coli	GH2	4.951896641	1
Kineococcus radiotolerans	GH2	3.03645742	1

Herpetosiphon aurantiacus	GH2	2.467219737	1
Hungateiclostridium thermocellum	GH1	4.161244363	1
Roseiflexus sp.	GH1	2.092405838	1
Exiguobacterium sp.	GH1	3.604041401	1
Rhizobium leguminosarum	GH2	2.765664497	1
Pseudoalteromonas atlantica	GH2	2.341949533	1
Capnocytophaga ochracea	GH2	3.168311961	1
Bacteroides fragilis	GH2	5.330440553	1
Paenarthrobacter aurescens	GH1	2.654575837	1
Jonesia denitrificans	GH1	2.93633819	1
Bifidobacterium animalis	GH2	4.326031942	1
Chitinophaga pinensis	GH2	3.132181861	1
Escherichia coli	GH2	4.951896641	1
Halanaerobium praevalens	GH2	3.109148225	1
Bacteroides vulgatus	GH2	5.981194327	1
Pseudoalteromonas atlantica	GH2	2.341949533	1
Mesoplasma florum	GH1	3.526749672	1
Streptococcus mutans	GH1	4.026249304	1
Streptomyces scabiei	GH1	2.583957419	1
Rhodococcus erythropolis	GH1	4.017441033	1
Pediococcus pentosaceus	GH1	3.700450677	1
Deinococcus geothermalis	CBM57	2.528732653	1
Lactobacillus paracasei	GH1	4.039963241	1
Paenibacillus polymyxa	GH1	3.727193955	1
Nocardioides sp.	GH1	2.528732653	1
Serratia proteamaculans	GH1	3.231309256	1
Fusobacterium mortiferum	GH1	2.652162063	1
Chromobacterium violaceum	GH1	3.658937709	1
Streptococcus suis	GH1	4.75869111	1
Halothermothrix orenii	GH1	3.329596854	1
Sanguibacter keddieii	GH1	2.855701308	1
Escherichia fergusonii	GH1	4.301024444	1
Lactobacillus sakei	GH1	3.906365165	1
Sinorhizobium fredii	GH1	2.853225066	1
Kitasatospora setae	GH1	2.897849609	1
Listeria welshimeri	GH1	3.164987093	1
Lactobacillus casei	GH1	4.054150519	1
Lactobacillus johnsonii	GH1	3.821988045	1
Lactobacillus amylovorus	GH1	4.651593108	1
Dickeya dadantii	GH2	3.173053214	1

Chitinophaga pinensis	GH2	3.132181861	1
Rhizobium etli	GH2	2.801391781	1
Bacteroides xylanisolvens	GH2	5.755848669	1
Escherichia coli	GH2	4.951896641	1
Koribacter versatilis	GH2	2.871309697	1
Shigella sonnei	GH2	4.50932433	1
Bacteroides fragilis	GH2	5.330440553	1
Saccharophagus degradans	GH2	2.105502422	0
Clavibacter michiganensis	GH2	3.019422612	1
Herpetosiphon aurantiacus	GH2	2.467219737	1
Agrobacterium radiobacter	GH1	2.773077398	1
Caulobacter segnis	GH2	2.163370478	1
Syntrophobacter fumaroxidans	GH2	3.09585751	1
Parabacteroides distasonis	GH2	5.5003263	1
Bacillus licheniformis	GH1	2.921693481	1
Geobacillus kaustophilus	GH1	3.176799249	1
Bacteroides vulgatus	GH2	5.981194327	1
Chitinophaga pinensis	GH2	3.132181861	1
Cutibacterium acnes	GH2	3.525541687	1
Saccharophagus degradans	GH2	2.105502422	0
Streptococcus pyogenes	GH2	4.645909728	1
Bacteroides xylanisolvens	GH2	5.755848669	1
Escherichia coli	GH2	4.951896641	1
Escherichia coli	GH1	4.951896641	1
Shewanella violacea	GH1	2.214505056	1
Stigmatella aurantiaca	GH1	2.897849609	1
Clostridium perfringens	GH1	4.092439715	1
Klebsiella pneumoniae	GH1	3.527499461	1
Shigella dysenteriae	GH2	4.389565899	1
Shigella sonnei	GH2	4.50932433	1
Bacillus clausii	GH1	3.122214447	1
Streptococcus pneumoniae	GH1	3.898009613	1
Lactobacillus plantarum	GH1	3.483288678	1
Micromonospora aurantiaca	GH1	2.656747513	1
Lactobacillus plantarum	GH1	3.483288678	1
Methylococcus capsulatus	GH1	2.920459839	1
Clostridium beijerinckii	GH1	4.172192327	1
Sodalis glossinidius	GH1	3.639349008	1
Streptococcus dysgalactiae	GH1	3.643525026	1
Rhodoferax ferrireducens	GH1	2.976100809	1

Ruminiclostridium cellulolyticum	GH1	4.261509628	1
Streptococcus pneumoniae	GH1	3.898009613	1
Hungateiclostridium thermocellum	GH1	4.161244363	1
Spiroplasma citri	GH1	1.093054293	1
Lactococcus lactis	GH1	4.535318878	1
Mesoplasma florum	GH1	3.526749672	1
Mesoplasma florum	GH1	3.526749672	1
Lactobacillus johnsonii	GH1	3.821988045	1
Lactobacillus paracasei	GH1	4.039963241	1
Salmonella agona	GH1	3.588748032	1
Clostridium saccharolyticum	GH1	5.056490176	1
Lactobacillus crispatus	GH1	3.911834731	1
Clostridium botulinum	GH1	4.053974807	1
Klebsiella pneumoniae	GH1	3.527499461	1
Lactobacillus johnsonii	GH1	3.821988045	1
Klebsiella pneumoniae	GH1	3.527499461	1
Thermodesulfovibrio yellowstonii	GH1	2.863608688	1
Streptococcus pneumoniae	GH1	3.898009613	1
Saccharophagus degradans	GH30	2.105502422	0
Salmonella typhimurium	GH30	3.278675607	1
Dyadobacter fermentans	GH30	3.417983563	1
Catenulispora acidiphila	GH30	2.886534124	1
Paludibacter propionicigenes	GH30	3.910160743	1
Cytophaga hutchinsonii	GH30	3.438614745	1
Clostridium botulinum	GH1	4.053974807	1
Escherichia coli	GH2	4.951896641	1
Thermotoga petrophila	GH2	1.906618403	0
Escherichia fergusonii	GH2	4.301024444	1
Caldicellulosiruptor owensensis	GH2	3.105077595	1
Paenibacillus sp.	GH30	3.716301192	1
Tolumonas auensis	GH1	3.248437991	1
Enterococcus faecium	GH2	2.801761627	1
Streptococcus suis	GH2	4.75869111	1
Alistipes shahii	GH2	5.507004832	1
Escherichia coli	GH2	4.951896641	1
Bacteroides vulgatus	GH2	5.981194327	1
Thermotoga neapolitana	GH2	2.194896294	1
Bacteroides thetaiotaomicron	GH2	5.668359112	1
Pantoea ananatis	GH1	3.19668615	1
Escherichia coli	GH1	4.951896641	1

Vibrio vulnificus	GH1	1.625869887	0
Lactobacillus crispatus	GH1	3.911834731	1
Zunongwangia profunda	GH2	3.493179318	1
Caldicellulosiruptor hydrothermalis	GH2	3.083906066	1
Spirosoma linguale	GH2	3.535049207	1
Escherichia coli	GH2	4.951896641	1
Thermotoga maritima	GH2	2.481956201	1
Ruminococcus sp.	GH2	5.524674293	1
Fibrobacter succinogenes	GH2	3.403591123	1
Escherichia coli	GH2	4.951896641	1
Bacteroides vulgatus	GH2	5.981194327	1
Faecalibacterium prausnitzii	GH2	5.703104557	1
Burkholderia pseudomallei	GH2	3.93219767	1
Shigella boydii	GH2	4.467400665	1
Escherichia coli	GH2	4.951896641	1
Beutenbergia cavernae	CBM57	2.742712219	1
Rhodothermus marinus	GH2	2.502529311	1
Gramella forsetii	GH2	3.311848451	1
Flavobacterium johnsoniae	GH2	3.525155341	1
Rhodococcus opacus	GH2	2.691346867	1
Bacteroides xylanisolvens	GH2	5.755848669	1
Thermoanaerobacter italicus	GH2	3.176195199	1
Bacillus pumilus	GH1	3.248670682	1
Novosphingobium aromaticivorans	GH1	0.766564697	0
Streptococcus pneumoniae	GH1	3.898009613	1
Phenylobacterium zucineum	GH1	2.898116603	1
Lactobacillus plantarum	GH1	3.483288678	1
Cronobacter sakazakii	GH2	3.663917102	1
Parabacteroides distasonis	GH2	5.5003263	1
Shigella dysenteriae	GH2	4.389565899	1
Bacteroides xylanisolvens	GH2	5.755848669	1
Halanaerobium hydrogeniformans	GH2	3.442305735	1
Vibrio campbellii	GH2	2.003060928	1
Escherichia coli	GH2	4.951896641	1
Brachybacterium faecium	GH2	2.322733424	1
Aliivibrio fischeri	GH2	2.148976013	1
Tolumonas auensis	GH2	3.248437991	1
Listeria welshimeri	GH1	3.164987093	1
Capnocytophaga ochracea	GH2	3.168311961	1
Paenibacillus sp.	GH2	3.716301192	1

Streptomyces bingchenggensis	GH2	2.546261927	1
Bacillus velezensis	GH1	3.007591831	1
Olsenella uli	GH1	4.211506708	1
Bifidobacterium animalis	GH1	4.326031942	1
Cronobacter sakazakii	GH1	3.663917102	1
Escherichia fergusonii	GH1	4.301024444	1
Lactococcus lactis	GH1	4.535318878	1
Roseburia intestinalis	GH2	5.494855325	1
Bacillus halodurans	GH1	3.382316202	1
Escherichia coli	GH1	4.951896641	1
Salmonella gallinarum	GH1	3.384673024	1
Pantoea ananatis	GH1	3.19668615	1
Streptococcus equi	GH1	3.491210821	1
Kosmotoga olearia	GH1	2.483235038	1
Streptococcus suis	GH1	4.75869111	1
Corynebacterium kroppenstedtii	GH1	2.748506441	1
Thermosipho africanus	GH1	2.118718832	0
Rhodobacter sphaeroides	GH1	2.423885576	1
Escherichia coli	GH2	4.951896641	1
Xanthomonas axonopodis	GH2	1.902261106	1
Halothermothrix orenii	GH2	3.329596854	1
Roseburia intestinalis	GH2	5.494855325	1
Caldicellulosiruptor bescii	GH2	3.125737391	1
Enterobacter sp.	GH2	4.156645836	1
Shigella flexneri	GH2	4.384218202	1
Salmonella choleraesuis	GH1	3.399683172	1
Enterobacter sp.	GH1	4.156645836	1
Escherichia coli	GH2	4.951896641	1
Enterococcus faecium	GH2	2.801761627	1
Bifidobacterium breve	GH2	4.551013712	1
Bacteroides thetaiotaomicron	GH2	5.668359112	1
Thermotoga maritima	GH2	2.481956201	1
Escherichia coli	GH1	4.951896641	1
Streptococcus suis	GH1	4.75869111	1
Lactococcus lactis	GH1	4.535318878	1
Paenibacillus polymyxa	GH2	3.727193955	1
Blautia obeum	GH2	5.41829818	1
Sebaldella termitidis	GH1	2.899978786	1
Streptococcus sanguinis	GH1	4.260660059	1
Geobacillus thermodenitrificans	GH1	3.274686288	1

Lactobacillus plantarum	GH1	3.483288678	1
Lactococcus lactis	GH1	4.535318878	1
Streptobacillus moniliformis	GH1	3.496122915	1
Coprococcus sp.	GH1	5.416845915	1
Treponema denticola	GH1	4.109719061	1
Bradyrhizobium sp.	GH1	2.919717144	1
Conexibacter woesei	GH1	2.992179949	1
Agrobacterium vitis	GH1	2.861500523	1
Tolumonas auensis	GH1	3.248437991	1
Halanaerobium hydrogeniformans	GH1	3.442305735	1
Catenulispora acidiphila	GH1	2.886534124	1
Klebsiella pneumoniae	GH1	3.527499461	1
Caldicellulosiruptor kronotskyensis	GH1	3.162669177	1
Mesoplasma florum	GH1	3.526749672	1
Psychromonas ingrahamii	GH1	2.828943938	1
Streptococcus pneumoniae	GH1	3.898009613	1
Escherichia coli	GH1	4.951896641	1
Roseiflexus castenholzii	GH2	1.70877002	1
Gardnerella vaginalis	GH2	3.828494186	1
Escherichia coli	GH2	4.951896641	1
Escherichia coli	GH2	4.951896641	1
Clostridium botulinum	GH30	4.053974807	1
Paenibacillus polymyxa	GH30	3.727193955	1
Cellvibrio japonicus	GH30	2.097488885	0
Pedobacter heparinus	GH30	3.537943992	1
Burkholderia pseudomallei	GH2	3.93219767	1
Escherichia coli	GH2	4.951896641	1
Citrobacter freundii	GH2	2.755642327	1
Pedobacter heparinus	GH2	3.537943992	1
Ruminococcus gnavus	GH2	5.39652351	1
Caldicellulosiruptor saccharolyticus	GH2	3.082502309	1
Escherichia coli	GH2	4.951896641	1
Shigella flexneri	GH2	4.384218202	1
Butyrivibrio proteoclasticus	GH2	4.213375923	1
Caldicellulosiruptor saccharolyticus	GH2	3.082502309	1
Paenibacillus sp.	GH2	3.716301192	1
Gordonia bronchialis	GH2	2.602291518	1
Xylella fastidiosa	GH2	2.313735638	1
Kineococcus radiotolerans	CBM57	3.03645742	1
Solibacter usitatus	CBM57	3.694382176	1

Acidobacterium capsulatum	CBM57	2.325925309	1
Sorangium cellulosum	CBM57	2.502583787	1
Micromonospora aurantiaca	CBM57	2.656747513	1
Lactococcus garvieae	GH1	2.40846932	1
Petrotoga mobilis	GH2	2.060153867	1
Dictyoglomus thermophilum	GH2	2.597989089	1
Faecalibacterium prausnitzii	GH2	5.703104557	1
Bacillus megaterium	GH2	3.09272887	1
Yersinia enterocolitica	GH2	3.206616339	1
Streptomyces coelicolor	GH2	1.758481979	1
Shigella flexneri	GH2	4.384218202	1
Thermotoga maritima	GH2	2.481956201	1
Frankia alni	GH30	2.650947694	1
Paenibacillus polymyxa	GH30	3.727193955	1
Streptomyces bingchenggensis	GH30	2.546261927	1
Bacteroides xylanisolvens	GH30	5.755848669	1
Caulobacter vibrioides	GH30	1.916277954	1
Xanthomonas axonopodis	GH30	1.902261106	1
Roseburia intestinalis	GH2	5.494855325	1
butyrate-producing bacterium	GH2	5.116587758	1
Lactobacillus gasseri	GH2	2.459107723	1
Escherichia coli	GH2	4.951896641	1
Lachnoclostridium phytofermentans	GH2	4.440620629	1
Paenibacillus polymyxa	GH2	3.727193955	1
Flavobacterium johnsoniae	GH2	3.525155341	1
Coraliomargarita akajimensis	GH2	3.018355261	1
Frankia inefficax	GH2	2.921941994	1
Ruminococcus sp.	GH2	5.524674293	1
Clostridioides difficile	GH1	5.039965306	1
Thermobifida fusca	GH1	2.489421356	1
Klebsiella pneumoniae	GH1	3.527499461	1
Haliangium ochraceum	GH1	2.906275029	1
Escherichia coli	GH1	4.951896641	1
Escherichia coli	GH1	4.951896641	1
Nostoc punctiforme	GH1	2.376682258	1
Azospirillum sp.	GH1	2.549653156	1
Ochrobactrum anthropi	GH1	3.03545983	1
Lactobacillus amylovorus	GH1	4.651593108	1
Streptomyces ambofaciens	GH1	2.51396916	1
Faecalibacterium prausnitzii	GH1	5.703104557	1

Enterococcus faecalis	GH1	4.493392116	1
Yersinia enterocolitica	GH1	3.206616339	1
Escherichia coli	GH1	4.951896641	1
Geobacter sp.	GH1	3.054419337	1
Nocardiopsis dassonvillei	GH1	2.731716088	1
Bifidobacterium adolescentis	GH1	5.563837622	1
Streptococcus uberis	GH1	4.209153639	1
Caldanaerobacter subterraneus	GH1	3.090958877	1
[Clostridium] cf.	GH1	5.281472964	1
Streptococcus pneumoniae	GH1	3.898009613	1
Serratia proteamaculans	GH1	3.231309256	1
Lactococcus lactis	GH1	4.535318878	1
Escherichia coli	GH1	4.951896641	1
Shigella boydii	GH1	4.467400665	1
Streptococcus uberis	GH1	4.209153639	1
Roseiflexus castenholzii	GH1	1.70877002	1
Bifidobacterium bifidum	GH1	5.013833965	1
Beutenbergia cavernae	GH2	2.742712219	1
Clostridium scindens	GH2	4.984203302	1
Cellvibrio japonicus	GH2	2.097488885	0
Streptomyces lasaliensis	GH2	2.493786818	1
Zunongwangia profunda	GH2	3.493179318	1
Caldicellulosiruptor hydrothermalis	GH1	3.083906066	1
Lactobacillus johnsonii	GH1	3.821988045	1
Streptomyces bingchenggensis	GH1	2.546261927	1
Sphingopyxis alaskensis	GH1	2.501442593	1
Spirosoma linguale	GH2	3.535049207	1
Pedobacter heparinus	GH2	3.537943992	1
Streptococcus pneumoniae	GH2	3.898009613	1
Escherichia coli	GH2	4.951896641	1
Geodermatophilus obscurus	GH2	2.636926973	1
Listeria monocytogenes	GH1	2.652162063	1
Clostridioides difficile	GH1	5.039965306	1
Clostridioides difficile	GH1	5.039965306	1
Chitinophaga pinensis	GH1	3.132181861	1
Azospirillum sp.	GH1	2.549653156	1
Bacillus subtilis	GH1	3.020649231	1
Escherichia coli	GH1	4.951896641	1
Escherichia coli	GH1	4.951896641	1
Escherichia coli	GH2	4.951896641	1

Catenulispora acidiphila	GH2	2.886534124	1
Bifidobacterium animalis	GH2	4.326031942	1
Lactobacillus brevis	GH2	3.475230992	1
Butyrivibrio fibrisolvens	GH2	4.079675342	1
Clostridium cellulovorans	GH2	4.295967255	1
Pedobacter heparinus	GH2	3.537943992	1
Escherichia coli	GH2	4.951896641	1
Chitinophaga pinensis	GH2	3.132181861	1
Aeromonas hydrophila	GH2	3.103122052	1
Tsukamurella paurometabola	GH2	2.358358956	1
Halothermothrix orenii	GH2	3.329596854	1
Rhizobium leguminosarum	GH2	2.765664497	1
Butyrivibrio proteoclasticus	GH2	4.213375923	1
Nostoc punctiforme	GH1	2.376682258	1
Bacillus subtilis	GH1	3.020649231	1
Faecalibacterium prausnitzii	GH2	5.703104557	1
Shigella boydii	GH2	4.467400665	1
Rhizobium leguminosarum	GH2	2.765664497	1
Caldicellulosiruptor kronotskyensis	GH2	3.162669177	1
Streptococcus thermophilus	GH2	3.238181275	1
Ruminococcus sp.	GH2	5.524674293	1
Dickeya zeae	GH1	3.05858332	1
Escherichia coli	GH2	4.951896641	1
Paraburkholderia xenovorans	GH2	3.175892682	1
Escherichia coli	GH2	4.951896641	1
Bacteroides thetaiotaomicron	GH2	5.668359112	1
Paenibacillus sp.	GH30	3.716301192	1
Alistipes shahii	GH2	5.507004832	1
Alistipes shahii	GH2	5.507004832	1
Bacteroides thetaiotaomicron	GH2	5.668359112	1
Methylobacterium radiotolerans	GH2	2.853564852	1
Butyrivibrio proteoclasticus	GH2	4.213375923	1
Streptococcus salivarius	GH2	4.820450348	1
Stigmatella aurantiaca	GH2	2.897849609	1
Bifidobacterium longum	GH2	5.049759625	1
Shewanella baltica	GH2	2.273258695	1
Bacteroides xylanisolvens	GH2	5.755848669	1
[Eubacterium] rectale	GH2	5.645131414	1
Thermotoga maritima	GH2	2.481956201	1
Vibrio vulnificus	GH2	1.625869887	0

Clostridium cellulovorans	GH2	4.295967255	1
Escherichia coli	GH2	4.951896641	1
Dickeya zeae	GH2	3.05858332	1
Geobacillus sp.	GH2	3.64830473	1
Teredinibacter turnerae	CBM57	2.455699954	1
Zunongwangia profunda	CBM57	3.493179318	1
Coraliomargarita akajimensis	CBM57	3.018355261	1
Gramella forsetii	CBM57	3.311848451	1
Frankia inefficax	CBM57	2.921941994	1
Methylorubrum extorquens	CBM57	2.890499779	1
Acidobacterium capsulatum	CBM57	2.325925309	1
Beutenbergia cavernae	GH2	2.742712219	1
Escherichia coli	GH2	4.951896641	1
Pedobacter heparinus	CBM57	3.537943992	1
Shewanella woodyi	CBM57	2.158127368	1
Maribacter sp.	CBM57	3.442416285	1
Bacteroides vulgatus	CBM57	5.981194327	1
Maribacter sp.	CBM57	3.442416285	1
Vibrio cholerae	GH1	2.401983537	1
Bifidobacterium adolescentis	GH30	5.563837622	1
Geobacillus sp.	GH30	3.64830473	1
Bacteroides vulgatus	GH30	5.981194327	1
Sanguibacter keddieii	GH2	2.855701308	1
Bacteroides fragilis	GH2	5.330440553	1
Beutenbergia cavernae	GH2	2.742712219	1
Propionibacterium freudenreichii	GH2	3.746868029	1
Faecalibacterium prausnitzii	GH2	5.703104557	1
Streptococcus thermophilus	GH2	3.238181275	1
Bacteroides thetaiotaomicron	GH2	5.668359112	1
Kribbella flavida	GH2	2.574757512	1
Bacteroides xylanisolvens	GH2	5.755848669	1
Shigella sonnei	GH2	4.50932433	1
Erwinia pyrifoliae	GH2	2.917976808	1
Bacteroides thetaiotaomicron	GH2	5.668359112	1
Gardnerella vaginalis	GH2	3.828494186	1
Streptomyces griseus	GH2	2.688132045	1
Agrobacterium vitis	GH2	2.861500523	1
Klebsiella pneumoniae	GH2	3.527499461	1
Rhizobium leguminosarum	GH1	2.765664497	1
Pantoea ananatis	GH1	3.19668615	1

Bradyrhizobium sp.	GH1	2.919717144	1
Caulobacter segnis	GH1	2.163370478	1
Bifidobacterium animalis	GH1	4.326031942	1
Lactococcus lactis	GH1	4.535318878	1
Clostridium beijerinckii	GH1	4.172192327	1
Gordonia bronchialis	GH1	2.602291518	1
Pseudothermotoga lettingae	GH1	2.003060928	1
Bacillus halodurans	GH1	3.382316202	1
Bacillus subtilis	GH1	3.020649231	1
Shewanella woodyi	GH1	2.158127368	1
Clostridium beijerinckii	GH1	4.172192327	1
Anaerococcus prevotii	GH1	3.578456843	1
Pectobacterium carotovorum	GH1	3.146442611	1
Paenibacillus polymyxa	GH1	3.727193955	1
Ruminiclostridium cellulolyticum	GH2	4.261509628	1
Escherichia coli	GH2	4.951896641	1
Bacteroides fragilis	GH2	5.330440553	1
Escherichia coli	GH2	4.951896641	1
Yersinia pseudotuberculosis	GH2	3.271820733	1
Roseiflexus sp.	GH2	2.092405838	1
Mesoplasma florum	GH1	3.526749672	1
Streptococcus pneumoniae	GH1	3.898009613	1
Rhizobium leguminosarum	GH1	2.765664497	1
Streptomyces coelicolor	GH1	1.758481979	1
Raoultella planticola	GH2	0.863253122	0
Shigella boydii	GH2	4.467400665	1
Bacteroides fragilis	GH2	5.330440553	1
Pediococcus pentosaceus	GH1	3.700450677	1
Halothermothrix orenii	GH2	3.329596854	1
Rhizobium etli	GH2	2.801391781	1
Bifidobacterium dentium	GH2	4.766725591	1
Sinorhizobium fredii	GH2	2.853225066	1
Xanthomonas campestris	GH2	2.836321535	1
Escherichia coli	GH1	4.951896641	1
Enterococcus faecalis	GH1	4.493392116	1
Streptococcus pneumoniae	GH1	3.898009613	1
Clostridioides difficile	GH1	5.039965306	1
Shigella flexneri	GH2	4.384218202	1
Geodermatophilus obscurus	GH30	2.636926973	1
Fibrobacter succinogenes	GH30	3.403591123	1

Ruminiclostridium cellulolyticum	GH2	4.261509628	1
Shigella flexneri	GH2	4.384218202	1
Caldicellulosiruptor owensensis	GH2	3.105077595	1
Lachnoclostridium phytofermentans	GH2	4.440620629	1
Chitinophaga pinensis	GH30	3.132181861	1
Catenulispora acidiphila	GH30	2.886534124	1
Paenibacillus sp.	GH30	3.716301192	1
Thermoanaerobacter mathranii	GH2	3.212248516	1
Teredinibacter turnerae	GH2	2.455699954	1
Escherichia coli	GH2	4.951896641	1
Parabacteroides distasonis	GH2	5.5003263	1
Escherichia coli	GH2	4.951896641	1
Psychromonas ingrahamii	GH2	2.828943938	1
Thermotoga maritima	GH2	2.481956201	1
Lactococcus lactis	GH2	4.535318878	1
Escherichia coli	GH2	4.951896641	1
Streptococcus thermophilus	GH2	3.238181275	1
Chitinophaga pinensis	GH2	3.132181861	1
Caldicellulosiruptor bescii	GH2	3.125737391	1
Frankia sp.	GH2	2.890273357	1
Bacteroides fragilis	GH2	5.330440553	1
Lactobacillus acidophilus	GH2	4.391043616	1
Escherichia coli	GH2	4.951896641	1
Pectobacterium carotovorum	GH1	3.146442611	1
Streptococcus mitis	GH1	4.432157949	1
Faecalibacterium prausnitzii	GH2	5.703104557	1
Paludibacter propionicigenes	GH2	3.910160743	1
Caldicellulosiruptor bescii	GH2	3.125737391	1
Chitinophaga pinensis	GH2	3.132181861	1
Shigella boydii	GH2	4.467400665	1
Escherichia coli	GH2	4.951896641	1
Bacteroides thetaiotaomicron	GH2	5.668359112	1
Escherichia coli	GH2	4.951896641	1
Clavibacter michiganensis	GH2	3.019422612	1
Sediminispirochaeta smaragdinae	GH2	4.100615895	1
Slackia heliotrinireducens	GH2	4.068621032	1
Solibacter usitatus	CBM57	3.694382176	1
Pedobacter heparinus	CBM57	3.537943992	1
Solibacter usitatus	CBM57	3.694382176	1
Saccharophagus degradans	CBM57	2.105502422	0

Opitutus terrae	CBM57	2.849000161	1
Enterobacter sp.	GH1	4.156645836	1
Lactobacillus acidophilus	GH1	4.391043616	1
Streptococcus equi	GH1	3.491210821	1
Escherichia coli	GH1	4.951896641	1
Caldicellulosiruptor hydrothermalis	GH2	3.083906066	1
Klebsiella pneumoniae	GH2	3.527499461	1
Stenotrophomonas maltophilia	GH2	2.815564918	1
Bacteroides fragilis	GH2	5.330440553	1
Faecalibacterium prausnitzii	GH2	5.703104557	1
Xanthomonas campestris	GH2	2.836321535	1
Opitutus terrae	GH2	2.849000161	1
Bacteroides thetaiotaomicron	CBM57	5.668359112	1
Teredinibacter turnerae	CBM57	2.455699954	1
Leuconostoc mesenteroides	GH1	4.403938925	1
Paenibacillus polymyxa	GH1	3.727193955	1
Corynebacterium urealyticum	GH1	3.15668063	1
Lactococcus lactis	GH1	4.535318878	1
Clostridioides difficile	GH1	5.039965306	1
Edwardsiella tarda	GH1	3.149009708	1
Spirosoma linguale	CBM57	3.535049207	1
Streptosporangium roseum	CBM57	2.74523594	1
Flavobacterium johnsoniae	CBM57	3.525155341	1
Robiginitalea biformata	CBM57	3.293346465	1
uncultured bacterium	GH1	4.006358463	1
Ruminococcus sp.	GH1	5.524674293	1
Coraliomargarita akajimensis	GH2	3.018355261	1
Klebsiella pneumoniae	GH2	3.527499461	1
Escherichia coli	GH2	4.951896641	1
Solibacter usitatus	GH2	3.694382176	1
Opitutus terrae	GH2	2.849000161	1
Burkholderia pseudomallei	GH79	3.93219767	1
Myxococcus xanthus	GH1	3.04510265	1
Streptococcus sanguinis	GH1	4.260660059	1
Thermoanaerobacter pseudethanolicus	GH1	3.370655894	1
Enterobacter lignolyticus	GH1	3.968988727	1
Saccharophagus degradans	GH1	2.105502422	0
Dickeya chrysanthemi	GH1	2.985934376	1
Pseudomonas aeruginosa	GH1	3.269274895	1
Lactobacillus paracasei	GH1	4.039963241	1

Caldicellulosiruptor hydrothermalis	GH1	3.083906066	1
Clostridioides difficile	GH1	5.039965306	1
Streptococcus equi	GH1	3.491210821	1
Aliivibrio fischeri	GH1	2.148976013	1
Paenarthrobacter aurescens	GH1	2.654575837	1
Mycolicibacterium gilvum	GH1	2.436189229	1
Escherichia coli	GH1	4.951896641	1
Saccharopolyspora erythraea	GH1	2.829954331	1
Streptomyces scabiei	GH30	2.583957419	1
Bifidobacterium breve	GH30	4.551013712	1
Paenibacillus polymyxa	GH30	3.727193955	1
Dictyoglomus turgidum	GH2	2.698198103	1
Zunongwangia profunda	GH30	3.493179318	1
Thermoanaerobacter mathranii	GH1	3.212248516	1
Streptosporangium roseum	GH1	2.74523594	1
Brachyspira murdochii	GH1	2.974668271	1
Clavibacter michiganensis	GH1	3.019422612	1
Streptosporangium roseum	GH1	2.74523594	1
Mesoplasma florum	GH1	3.526749672	1
Bacillus subtilis	GH1	3.020649231	1
Thermoanaerobacter mathranii	GH1	3.212248516	1
Lactococcus lactis	GH1	4.535318878	1
Deinococcus geothermalis	GH1	2.528732653	1
Bacillus pumilus	GH1	3.248670682	1
Paludibacter propionicigenes	GH2	3.910160743	1
Serratia sp.	GH2	0.54672922	0
Escherichia coli	GH2	4.951896641	1
Kyrpidia tusciae	GH2	2.876857391	1
Sebaldella termitidis	GH2	2.899978786	1
Lactobacillus crispatus	GH1	3.911834731	1
Lactobacillus plantarum	GH1	3.483288678	1
Dickeya paradisiaca	GH1	3.018719322	1
Pediococcus pentosaceus	GH1	3.700450677	1
Bacillus cereus	GH1	3.080867958	1
Stackebrandtia nassauensis	GH1	2.705407885	1
Rhodococcus erythropolis	GH1	4.017441033	1
Streptococcus equi	GH1	3.491210821	1
Mannheimia succiniciproducens	GH2	2.754523878	1
Pedobacter heparinus	GH2	3.537943992	1
Streptococcus thermophilus	GH2	3.238181275	1

Clostridium cellulovorans	GH2	4.295967255	1
Escherichia coli	GH2	4.951896641	1
Caldicellulosiruptor obsidiansis	GH30	3.232298508	1
Streptomyces coelicolor	GH30	1.758481979	1
Hungateiclostridium thermocellum	GH30	4.161244363	1
Coprococcus sp.	GH2	5.416845915	1
Bifidobacterium adolescentis	GH2	5.563837622	1
Shigella boydii	GH2	4.467400665	1
Bacteroides fragilis	GH2	5.330440553	1
Escherichia coli	GH2	4.951896641	1
Chitinophaga pinensis	GH2	3.132181861	1
Alistipes shahii	GH2	5.507004832	1
Serratia proteamaculans	GH2	3.231309256	1
Eggerthella lenta	GH2	4.840276105	1
Actinobacillus pleuropneumoniae	GH2	2.854550453	1
Stenotrophomonas maltophilia	GH2	2.815564918	1
Geobacillus sp.	GH2	3.64830473	1
Butyrivibrio fibrisolvens	GH2	4.079675342	1
Streptomyces ambofaciens	GH2	2.51396916	1
Bacteroides fragilis	GH2	5.330440553	1
Enterobacter lignolyticus	GH2	3.968988727	1
[Eubacterium] siraeum	GH2	5.238059667	1
Bifidobacterium longum	GH2	5.049759625	1
Bifidobacterium bifidum	GH2	5.013833965	1
Streptococcus thermophilus	GH2	3.238181275	1
Saccharopolyspora erythraea	GH2	2.829954331	1
Koribacter versatilis	GH2	2.871309697	1
Flavobacterium johnsoniae	GH2	3.525155341	1
Koribacter versatilis	GH2	2.871309697	1
Shigella flexneri	GH2	4.384218202	1
Petrotoga mobilis	GH1	2.060153867	1
Lactobacillus paracasei	GH2	4.039963241	1
Frankia sp.	GH1	2.890273357	1
Marinomonas sp.	GH2	3.075451459	1
[Eubacterium] rectale	GH2	5.645131414	1
Roseburia intestinalis	GH2	5.494855325	1
Bacillus megaterium	GH2	3.09272887	1
Cellvibrio japonicus	GH30	2.097488885	0
Bifidobacterium dentium	GH30	4.766725591	1
Leuconostoc citreum	GH1	3.622436533	1

Butyrivibrio fibrisolvens	GH1	4.079675342	1
Enterococcus faecium	GH1	2.801761627	1
Anaerococcus prevotii	GH1	3.578456843	1
Paenibacillus polymyxa	GH1	3.727193955	1
Sinorhizobium medicae	GH1	2.840198453	1
Pectobacterium carotovorum	GH1	3.146442611	1
Pedobacter heparinus	GH1	3.537943992	1
Enterococcus faecalis	GH1	4.493392116	1
Bacillus subtilis	GH1	3.020649231	1
Dickeya paradisiaca	GH1	3.018719322	1
[Eubacterium] rectale	GH1	5.645131414	1
Klebsiella pneumoniae	GH1	3.527499461	1
Paenibacillus polymyxa	GH1	3.727193955	1
Dickeya dadantii	GH1	3.173053214	1
Streptococcus dysgalactiae	GH1	3.643525026	1
Staphylococcus aureus	GH1	3.253816682	1
Klebsiella aerogenes	GH1	1.984853794	0
Streptococcus pyogenes	GH1	4.645909728	1
Lactobacillus crispatus	GH1	3.911834731	1
Streptococcus uberis	GH1	4.209153639	1
Shigella dysenteriae	GH1	4.389565899	1
Thermoanaerobacter sp.	GH1	3.471840657	1
Mycolicibacterium vanbaalenii	GH1	2.528732653	1
Lactobacillus crispatus	GH1	3.911834731	1
Clostridium cellulovorans	GH1	4.295967255	1
Corynebacterium jeikeium	GH1	3.210561975	1
Escherichia coli	GH1	4.951896641	1
Staphylococcus aureus	GH1	3.253816682	1
Citrobacter koseri	GH1	4.495223425	1
Streptococcus pyogenes	GH1	4.645909728	1
Streptococcus sanguinis	GH1	4.260660059	1
Rhodopseudomonas palustris	GH1	2.986919212	1
Escherichia coli	GH1	4.951896641	1
Thermotoga maritima	GH2	2.481956201	1
Streptococcus thermophilus	GH2	3.238181275	1
Histophilus somni	GH2	2.990607381	1
Spirosoma linguale	GH2	3.535049207	1
Streptococcus mitis	GH2	4.432157949	1
Escherichia coli	GH1	4.951896641	1
Lactobacillus plantarum	GH1	3.483288678	1

Phenylobacterium zucineum	GH1	2.898116603	1
Bradyrhizobium diazoefficiens	GH1	3.166834227	1
Enterobacter sp.	GH1	4.156645836	1
Klebsiella pneumoniae	GH1	3.527499461	1
Streptococcus pyogenes	GH1	4.645909728	1
Paludibacter propionicigenes	GH2	3.910160743	1
Bifidobacterium dentium	GH2	4.766725591	1
Salmonella arizonae	GH1	3.769206766	1
Escherichia coli	GH1	4.951896641	1
Escherichia coli	GH1	4.951896641	1
Escherichia coli	GH1	4.951896641	1
Clostridium beijerinckii	GH1	4.172192327	1
Halothermothrix orenii	GH1	3.329596854	1
Faecalibacterium prausnitzii	GH1	5.703104557	1
Catenulispora acidiphila	GH1	2.886534124	1
Erwinia billingiae	GH1	3.247037685	1
Escherichia coli	GH2	4.951896641	1
Akkermansia muciniphila	GH2	5.317459779	1
Novosphingobium aromaticivorans	GH2	0.766564697	0
Agrobacterium vitis	GH2	2.861500523	1
Chitinophaga pinensis	GH2	3.132181861	1
Lactobacillus plantarum	GH1	3.483288678	1
Mycoplasma penetrans	GH1	2.707941934	1
Akkermansia muciniphila	GH2	5.317459779	1
Opitutus terrae	GH2	2.849000161	1
Koribacter versatilis	GH1	2.871309697	1
Lactococcus lactis	GH1	4.535318878	1
Stigmatella aurantiaca	GH1	2.897849609	1
Lactobacillus sakei	GH1	3.906365165	1
Rhizobium leguminosarum	GH1	2.765664497	1
Solibacter usitatus	GH2	3.694382176	1
Chitinophaga pinensis	GH2	3.132181861	1
Enterococcus faecalis	GH1	4.493392116	1
Clostridium cellulovorans	GH1	4.295967255	1
Lactococcus lactis	GH1	4.535318878	1
Lactococcus lactis	GH1	4.535318878	1
Enterobacter lignolyticus	GH1	3.968988727	1
Rothia dentocariosa	GH1	3.957202895	1
Opitutus terrae	GH2	2.849000161	1
Caldicellulosiruptor bescii	GH2	3.125737391	1

Bacteroides thetaiotaomicron	GH30	5.668359112	1
Escherichia coli	GH2	4.951896641	1
Sebaldella termitidis	GH2	2.899978786	1
Saccharopolyspora erythraea	GH2	2.829954331	1
Lactococcus lactis	GH2	4.535318878	1
Caldicellulosiruptor kronotskyensis	GH2	3.162669177	1
Bacteroides thetaiotaomicron	GH2	5.668359112	1
Ruminococcus gnavus	GH2	5.39652351	1
Escherichia coli	GH2	4.951896641	1
Escherichia coli	GH2	4.951896641	1
Flavobacterium johnsoniae	GH2	3.525155341	1
Ruminiclostridium cellulolyticum	GH2	4.261509628	1
Faecalibacterium prausnitzii	GH2	5.703104557	1
Coraliomargarita akajimensis	GH2	3.018355261	1
Clostridium perfringens	GH2	4.092439715	1
Shigella flexneri	GH2	4.384218202	1
Gramella forsetii	GH2	3.311848451	1
Burkholderia pseudomallei	GH2	3.93219767	1
Chloroflexus aurantiacus	GH2	1.343763416	1
Streptococcus pyogenes	GH2	4.645909728	1
Bacteroides vulgatus	GH2	5.981194327	1
Rhodococcus opacus	GH1	2.691346867	1
Escherichia coli	GH1	4.951896641	1
Streptococcus uberis	GH1	4.209153639	1
Listeria innocua	GH1	2.642937634	1
Pseudoalteromonas atlantica	GH2	2.341949533	1
Bacteroides fragilis	GH2	5.330440553	1
Streptococcus thermophilus	GH2	3.238181275	1
Frankia inefficax	GH2	2.921941994	1
Escherichia coli	GH1	4.951896641	1
Butyrivibrio fibrisolvens	GH1	4.079675342	1
Pseudothermotoga lettingae	GH1	2.003060928	1
Shewanella frigidimarina	GH1	2.793897044	1
Oceanobacillus iheyensis	GH1	3.188323218	1
Bacillus megaterium	GH1	3.09272887	1
Streptomyces griseus	GH1	2.688132045	1
Streptococcus pyogenes	GH1	4.645909728	1
Actinosynnema mirum	GH1	2.698718791	1
Nocardiopsis dassonvillei	GH1	2.731716088	1
Thermobispora bispora	GH1	2.603002819	1

Beutenbergia cavernae	GH1	2.742712219	1
Pantoea vagans	GH1	3.252520094	1
Clostridium acetobutylicum	GH1	1.970128234	0
Lactobacillus plantarum	GH1	3.483288678	1
Sorangium cellulosum	GH30	2.502583787	1
Teredinibacter turnerae	GH30	2.455699954	1
Dyadobacter fermentans	GH30	3.417983563	1
Actinobacillus pleuropneumoniae	GH2	2.854550453	1
Streptococcus thermophilus	GH2	3.238181275	1
Catenulispora acidiphila	GH2	2.886534124	1
Coraliomargarita akajimensis	GH2	3.018355261	1
Bacteroides vulgatus	GH2	5.981194327	1
Shigella flexneri	GH2	4.384218202	1
Clostridium perfringens	GH2	4.092439715	1
Mycoplasma penetrans	GH1	2.707941934	1
Lactobacillus acidophilus	GH1	4.391043616	1
Prevotella ruminicola	GH2	4.464744795	1
Saccharophagus degradans	GH2	2.105502422	0
Yersinia pseudotuberculosis	GH2	3.271820733	1
Agathobacter rectalis	GH2	5.641970263	1
Streptobacillus moniliformis	GH2	3.496122915	1
Lactobacillus fermentum	GH2	4.102183676	1
Streptomyces bingchenggensis	GH2	2.546261927	1
Pantoea ananatis	GH1	3.19668615	1
Cutibacterium acnes	GH1	3.525541687	1
Erwinia amylovora	GH1	2.90156558	1
Sphingobium japonicum	GH1	2.420100395	1
Paenibacillus polymyxa	GH1	3.727193955	1
Lactobacillus helveticus	GH1	4.191208361	1
Thermoanaerobacterium thermosaccharolyticum	GH1	3.608969791	1
Staphylococcus aureus	GH1	3.253816682	1
Clavibacter michiganensis	GH2	3.019422612	1
Thermoanaerobacter sp.	GH2	3.471840657	1
Escherichia coli	GH2	4.951896641	1
Streptobacillus moniliformis	GH1	3.496122915	1
Lactobacillus johnsonii	GH1	3.821988045	1
Clavibacter michiganensis	GH1	3.019422612	1
Lactococcus lactis	GH1	4.535318878	1
Faecalibacterium prausnitzii	GH1	5.703104557	1
Brachyspira pilosicoli	GH1	3.888206756	1

Bacillus clausii	GH1	3.122214447	1
Salinispora arenicola	GH1	2.461696715	1
Streptomyces griseus	GH1	2.688132045	1
Streptomyces griseus	GH1	2.688132045	1
Paenibacillus sp.	GH2	3.716301192	1
Bacteroides vulgatus	GH2	5.981194327	1
Paenibacillus sp.	GH2	3.716301192	1
Escherichia coli	GH2	4.951896641	1
Thermomonospora curvata	GH1	2.806528372	1
Leptothrix cholodnii	GH1	3.372479372	1
Cyanothece sp.	GH1	2.72078854	1
Lactobacillus crispatus	GH1	3.911834731	1
Citrobacter koseri	GH1	4.495223425	1
Escherichia coli	GH1	4.951896641	1
Klebsiella pneumoniae	GH1	3.527499461	1
Ruminococcus gnavus	GH1	5.39652351	1
Salmonella typhimurium	GH1	3.278675607	1
Thermosipho africanus	GH1	2.118718832	0
Paenibacillus polymyxa	GH1	3.727193955	1
Faecalitalea cylindroides	GH1	5.253809626	1
Lactococcus lactis	GH1	4.535318878	1
Bifidobacterium longum	GH1	5.049759625	1
Frankia sp.	GH2	2.890273357	1
Paenibacillus sp.	GH2	3.716301192	1
Pseudarthrobacter chlorophenolicus	GH1	2.741360004	1
Chitinophaga pinensis	GH2	3.132181861	1
Streptococcus uberis	GH1	4.209153639	1
Dickeya paradisiaca	GH1	3.018719322	1
Kineococcus radiotolerans	GH1	3.03645742	1
Dickeya paradisiaca	GH1	3.018719322	1
Saccharophagus degradans	GH1	2.105502422	0
Sebaldella termitidis	GH1	2.899978786	1
Sebaldella termitidis	GH1	2.899978786	1
Mesoplasma florum	GH1	3.526749672	1
Butyrivibrio proteoclasticus	GH1	4.213375923	1
Bifidobacterium longum	GH2	5.049759625	1
Streptococcus pneumoniae	GH2	3.898009613	1
Roseburia intestinalis	GH2	5.494855325	1
[Eubacterium] rectale	GH2	5.645131414	1
Streptomyces bingchenggensis	GH2	2.546261927	1

Alteromonas mediterranea	GH2	2.114222949	1
Escherichia coli	GH2	4.951896641	1
Bacillus selenitireducens	GH2	3.381057312	1
Rhizobium etli	GH1	2.801391781	1
Dickeya chrysanthemi	GH1	2.985934376	1
Pedobacter heparinus	GH2	3.537943992	1
Porphyromonas gingivalis	GH2	4.203215583	1
Pectobacterium carotovorum	GH1	3.146442611	1
Klebsiella pneumoniae	GH1	3.527499461	1
Clostridium saccharolyticum	GH1	5.056490176	1
Streptococcus pyogenes	GH1	4.645909728	1
Paraburkholderia phytofirmans	GH1	3.21698638	1
Bacillus pumilus	GH1	3.248670682	1
Escherichia coli	GH1	4.951896641	1
Rubrobacter xylanophilus	GH1	2.512074083	1
Lactococcus lactis	GH1	4.535318878	1
Streptococcus pyogenes	GH1	4.645909728	1
Klebsiella pneumoniae	GH1	3.527499461	1
Clostridium beijerinckii	GH1	4.172192327	1
Bifidobacterium longum	GH2	5.049759625	1
Escherichia coli	GH2	4.951896641	1
Shigella dysenteriae	GH2	4.389565899	1
Lactobacillus johnsonii	GH1	3.821988045	1
Staphylococcus carnosus	GH1	3.461188466	1
Petrotoga mobilis	GH1	2.060153867	1
Bacillus subtilis	GH1	3.020649231	1
Listeria innocua	GH1	2.642937634	1
Lactobacillus casei	GH1	4.054150519	1
Clostridium perfringens	GH1	4.092439715	1
Rhodopseudomonas palustris	GH1	2.986919212	1
Curvibacter putative	GH2	1.139914283	0
Escherichia coli	GH2	4.951896641	1
Streptococcus equi	GH2	3.491210821	1
Nocardiopsis dassonvillei	GH2	2.731716088	1
Geobacillus kaustophilus	GH1	3.176799249	1
Escherichia coli	GH1	4.951896641	1
Stigmatella aurantiaca	GH1	2.897849609	1
Streptococcus pneumoniae	GH1	3.898009613	1
Oenococcus oeni	GH1	3.610261575	1
Klebsiella pneumoniae	GH1	3.527499461	1

Escherichia coli	GH1	4.951896641	1
Ruminococcus gnavus	GH2	5.39652351	1
Geobacillus sp.	GH30	3.64830473	1
Kitasatospora setae	GH30	2.897849609	1
Stigmatella aurantiaca	GH30	2.897849609	1
Escherichia coli	GH2	4.951896641	1
Alicyclobacillus acidocaldarius	GH2	3.380511029	1
Thermobifida fusca	GH2	2.489421356	1
Opitutus terrae	GH30	2.849000161	1
Kribbella flavida	GH30	2.574757512	1
Colwellia psychrerythraea	GH30	2.109088489	1
Fibrobacter succinogenes	GH30	3.403591123	1
Caldicellulosiruptor hydrothermalis	GH30	3.083906066	1
Xanthomonas axonopodis	GH30	1.902261106	1
Bifidobacterium longum	GH30	5.049759625	1
Methylobacillus flagellatus	GH2	3.28551259	1
Stigmatella aurantiaca	GH2	2.897849609	1
Ruminiclostridium cellulolyticum	GH30	4.261509628	1
Streptomyces scabiei	GH30	2.583957419	1
Micromonospora aurantiaca	GH30	2.656747513	1
Butyrivibrio fibrisolvens	GH30	4.079675342	1
Streptococcus dysgalactiae	GH1	3.643525026	1
Escherichia coli	GH1	4.951896641	1
Faecalitalea cylindroides	GH1	5.253809626	1
Lactobacillus reuteri	GH2	3.257320782	1
Brachyspira murdochii	GH2	2.974668271	1
Bacteroides fragilis	GH2	5.330440553	1
Lactobacillus johnsonii	GH1	3.821988045	1
Pantoea vagans	GH1	3.252520094	1
Bacteroides xylanisolvens	GH2	5.755848669	1
Bacteroides vulgatus	GH2	5.981194327	1
Cellvibrio japonicus	GH2	2.097488885	0
Catenulispora acidiphila	GH30	2.886534124	1
Caulobacter segnis	GH30	2.163370478	1
Halothermothrix orenii	GH30	3.329596854	1
Clostridium perfringens	GH2	4.092439715	1
Brevundimonas subvibrioides	GH2	2.525486274	1
Shigella flexneri	GH2	4.384218202	1
Brachyspira pilosicoli	GH1	3.888206756	1
Yersinia enterocolitica	GH1	3.206616339	1

Faecalibacterium prausnitzii	GH1	5.703104557	1
Micromonospora aurantiaca	GH2	2.656747513	1
Escherichia coli	GH2	4.951896641	1
Leuconostoc lactis	GH2	2.868933009	1
Pedobacter heparinus	GH2	3.537943992	1
Thermomonospora curvata	GH1	2.806528372	1
Xanthomonas axonopodis	GH30	1.902261106	1
Acidobacterium capsulatum	GH30	2.325925309	1
Lactobacillus brevis	GH30	3.475230992	1
Zunongwangia profunda	GH30	3.493179318	1
Streptomyces scabiei	GH30	2.583957419	1
Fibrobacter succinogenes	GH30	3.403591123	1
Bifidobacterium longum	GH2	5.049759625	1
Parabacteroides distasonis	GH2	5.5003263	1
Aliivibrio fischeri	GH2	2.148976013	1
Bacteroides thetaiotaomicron	GH2	5.668359112	1
Saccharopolyspora erythraea	GH1	2.829954331	1
Bacillus velezensis	GH30	3.007591831	1
Dickeya dadantii	GH30	3.173053214	1
Capnocytophaga ochracea	GH30	3.168311961	1
Stigmatella aurantiaca	GH30	2.897849609	1
Dickeya paradisiaca	GH30	3.018719322	1
Bradyrhizobium sp.	GH1	2.919717144	1
Mannheimia succiniciproducens	GH2	2.754523878	1
Escherichia coli	GH2	4.951896641	1
Bacteroides fragilis	GH2	5.330440553	1
Haemophilus parainfluenzae	GH2	4.7186941	1
Yersinia pestis	GH2	3.433167805	1
Bacteroides thetaiotaomicron	GH2	5.668359112	1
Truepera radiovictrix	GH2	2.750266906	1
Cronobacter sakazakii	GH1	3.663917102	1
Clostridium acetobutylicum	GH1	1.970128234	0
Lactococcus lactis	GH1	4.535318878	1
Frankia inefficax	GH1	2.921941994	1
Klebsiella pneumoniae	GH1	3.527499461	1
Caulobacter sp.	GH2	2.573978441	1
Chitinophaga pinensis	GH2	3.132181861	1
Xanthomonas campestris	GH2	2.836321535	1
Acaryochloris marina	GH2	2.383863923	1
Shigella sp.	GH2	1.565835599	1

Streptococcus thermophilus	GH2	3.238181275	1
Paenibacillus polymyxa	GH30	3.727193955	1
Caldicellulosiruptor saccharolyticus	GH30	3.082502309	1
Bacteroides xylanisolvens	GH2	5.755848669	1
Caulobacter sp.	GH1	2.573978441	1
Novosphingobium aromaticivorans	GH1	0.766564697	0
Bacillus thuringiensis	GH1	3.037291773	1
Lactobacillus acidophilus	GH1	4.391043616	1
Sebaldella termitidis	GH1	2.899978786	1
Fervidobacterium nodosum	GH1	2.056375335	1
Coprococcus sp.	GH1	5.416845915	1
Staphylococcus sciuri	GH1	0.576337049	0
Saccharomonospora viridis	GH2	2.402906227	1
Paludibacter propionicigenes	GH2	3.910160743	1
Shigella sp.	GH2	1.565835599	1
Clostridium cellulovorans	GH1	4.295967255	1
Burkholderia sp.	GH1	3.185120973	1
Lactobacillus crispatus	GH1	3.911834731	1
Streptococcus uberis	GH1	4.209153639	1
Bacillus cereus	GH1	3.080867958	1
Bifidobacterium adolescentis	GH1	5.563837622	1
Nocardia farcinica	GH1	2.681048152	1
Shigella sonnei	GH1	4.50932433	1
Streptococcus pneumoniae	GH1	3.898009613	1
Enterobacter cloacae	GH2	2.178925626	1
Leptotrichia buccalis	GH1	3.234674622	1
Lactobacillus plantarum	GH1	3.483288678	1
Streptococcus gordonii	GH1	4.329159897	1
Bifidobacterium animalis	GH2	4.326031942	1
Edwardsiella tarda	GH2	3.149009708	1
Bifidobacterium bifidum	GH2	5.013833965	1
Lactococcus garvieae	GH1	2.40846932	1
Lactobacillus crispatus	GH1	3.911834731	1
Dickeya chrysanthemi	GH1	2.985934376	1
Dickeya chrysanthemi	GH1	2.985934376	1
Escherichia coli	GH1	4.951896641	1
Streptomyces bingchenggensis	GH2	2.546261927	1
Listeria welshimeri	GH1	3.164987093	1
Salmonella typhimurium	GH30	3.278675607	1
Opitutus terrae	GH30	2.849000161	1

Spirosoma linguale	GH30	3.535049207	1
Bifidobacterium animalis	GH30	4.326031942	1
Staphylococcus epidermidis	GH1	3.151773558	1
butyrate-producing bacterium	GH1	5.116587758	1
Flavobacterium johnsoniae	GH2	3.525155341	1
Shigella flexneri	GH2	4.384218202	1
Lactobacillus amylovorus	GH1	4.651593108	1
Lactobacillus johnsonii	GH1	3.821988045	1
Lactococcus garvieae	GH1	2.40846932	1
Chitinophaga pinensis	GH2	3.132181861	1
Escherichia coli	GH2	4.951896641	1
Shigella sonnei	GH2	4.50932433	1
Bacteroides fragilis	GH2	5.330440553	1
Bacillus licheniformis	GH1	2.921693481	1
Roseburia intestinalis	GH1	5.494855325	1
Lactococcus lactis	GH1	4.535318878	1
Paenibacillus polymyxa	GH30	3.727193955	1
Bacteroides vulgatus	GH30	5.981194327	1
Sebaldella termitidis	GH1	2.899978786	1
Staphylococcus aureus	GH1	3.253816682	1
Salmonella arizonae	GH2	3.769206766	1
Paenibacillus sp.	GH2	3.716301192	1
Bacteroides fragilis	GH2	5.330440553	1
Solibacter usitatus	GH2	3.694382176	1
Butyrivibrio proteoclasticus	GH2	4.213375923	1
Caldanaerobacter subterraneus	GH30	3.090958877	1
Leadbetterella byssophila	GH30	3.698360007	1
Pantoea vagans	GH1	3.252520094	1
Bacteroides vulgatus	GH2	5.981194327	1
Lactobacillus delbrueckii	GH2	4.330538982	1
Streptococcus dysgalactiae	GH1	3.643525026	1
Oenococcus oeni	GH30	3.610261575	1
Fervidobacterium nodosum	GH30	2.056375335	1
Yersinia pestis	GH2	3.433167805	1
Paenibacillus polymyxa	GH2	3.727193955	1
Staphylococcus haemolyticus	GH2	3.184925706	1
Clostridium beijerinckii	GH30	4.172192327	1
Clostridium saccharolyticum	GH30	5.056490176	1
Caldicellulosiruptor obsidiansis	GH1	3.232298508	1
Salmonella paratyphi	GH1	3.414481535	1

Deinococcus deserti	GH1	2.963585295	1
Lactococcus lactis	GH1	4.535318878	1
Saccharopolyspora erythraea	GH2	2.829954331	1
Ruminococcus gnavus	GH1	5.39652351	1
Sorangium cellulosum	GH1	2.502583787	1
Lactococcus lactis	GH1	4.535318878	1
Jonesia denitrificans	GH2	2.93633819	1
[Eubacterium] rectale	GH2	5.645131414	1
Escherichia coli	GH2	4.951896641	1
Bacteroides vulgatus	GH2	5.981194327	1
Shigella sonnei	GH2	4.50932433	1
[Eubacterium] rectale	GH2	5.645131414	1
Spirosoma linguale	GH2	3.535049207	1
Shigella dysenteriae	GH2	4.389565899	1
Escherichia coli	GH2	4.951896641	1
Escherichia coli	GH2	4.951896641	1
Edwardsiella tarda	GH2	3.149009708	1
Escherichia coli	GH2	4.951896641	1
Escherichia coli	GH2	4.951896641	1
Vibrio vulnificus	GH2	1.625869887	0
Catenulispora acidiphila	GH2	2.886534124	1
Brachyspira murdochii	GH2	2.974668271	1
Bifidobacterium longum	GH2	5.049759625	1
Catenulispora acidiphila	GH2	2.886534124	1
Escherichia coli	GH1	4.951896641	1
Streptococcus pyogenes	GH1	4.645909728	1
Escherichia coli	GH2	4.951896641	1
Conexibacter woesei	GH2	2.992179949	1
Streptococcus pneumoniae	GH1	3.898009613	1
Arthrobacter sp.	GH1	2.788922725	1
Streptococcus pyogenes	GH1	4.645909728	1
Staphylococcus aureus	GH1	3.253816682	1
Thermoanaerobacter italicus	GH1	3.176195199	1
Lactococcus lactis	GH1	4.535318878	1
Dickeya dadantii	GH1	3.173053214	1
Lactococcus lactis	GH1	4.535318878	1
Bacillus subtilis	GH1	3.020649231	1
Streptococcus pneumoniae	GH1	3.898009613	1
Staphylococcus aureus	GH1	3.253816682	1
Micromonospora aurantiaca	GH1	2.656747513	1

Staphylococcus aureus	GH1	3.253816682	1
Shigella boydii	GH1	4.467400665	1
Bacteroides xylanisolvens	GH2	5.755848669	1
Actinosynnema mirum	GH30	2.698718791	1
Bacillus pumilus	GH1	3.248670682	1
Streptococcus uberis	GH1	4.209153639	1
Corynebacterium glutamicum	GH1	2.510167543	1
Lactobacillus acidophilus	GH1	4.391043616	1
Erwinia amylovora	GH1	2.90156558	1
Bacteroides fragilis	GH2	5.330440553	1
Kribbella flavida	GH1	2.574757512	1
Listeria monocytogenes	GH1	2.652162063	1
Clostridium botulinum	GH1	4.053974807	1
Streptococcus uberis	GH1	4.209153639	1
Ruminococcus gnavus	GH1	5.39652351	1
Clostridioides difficile	GH1	5.039965306	1
Staphylococcus aureus	GH1	3.253816682	1
Halothermothrix orenii	GH1	3.329596854	1
Yersinia enterocolitica	GH1	3.206616339	1
Clostridium botulinum	GH1	4.053974807	1
Saccharopolyspora erythraea	GH1	2.829954331	1
Clostridium perfringens	GH1	4.092439715	1
Rhodobacter capsulatus	GH1	2.871309697	1
Streptococcus dysgalactiae	GH1	3.643525026	1
Roseburia intestinalis	GH2	5.494855325	1
Dickeya zeae	GH1	3.05858332	1
Thermosipho melanesiensis	GH1	1.894736198	0
Bacteroides fragilis	GH2	5.330440553	1
Roseiflexus sp.	GH2	2.092405838	1
Nocardiopsis dassonvillei	GH1	2.731716088	1
Caldicellulosiruptor saccharolyticus	GH1	3.082502309	1
Streptococcus pyogenes	GH1	4.645909728	1
Akkermansia muciniphila	GH2	5.317459779	1
Brachybacterium faecium	GH2	2.322733424	1
Kosmotoga olearia	GH1	2.483235038	1
Bacillus subtilis	GH1	3.020649231	1
Shewanella piezotolerans	GH1	2.071245846	1
[Clostridium] cf.	GH2	5.281472964	1
Bacillus subtilis	GH30	3.020649231	1
Sorangium cellulosum	GH30	2.502583787	1

Paludibacter propionicigenes	GH30	3.910160743	1
Acidobacterium capsulatum	GH30	2.325925309	1
Kribbella flavida	GH30	2.574757512	1
Clostridium acetobutylicum	GH30	1.970128234	0
Stigmatella aurantiaca	GH30	2.897849609	1
Caldanaerobacter subterraneus	GH30	3.090958877	1
Spirosoma linguale	GH1	3.535049207	1
Roseiflexus sp.	GH1	2.092405838	1
Allochromatium vinosum	GH1	2.752017149	1
Caldicellulosiruptor bescii	GH30	3.125737391	1
Shigella boydii	GH2	4.467400665	1
Enterobacter cloacae	GH2	2.178925626	1
Streptococcus equi	GH2	3.491210821	1
Caldicellulosiruptor obsidiansis	GH2	3.232298508	1
Sebaldella termitidis	GH1	2.899978786	1
Arthrobacter sp.	GH2	2.788922725	1
Xylella fastidiosa	GH2	2.313735638	1
Lactococcus lactis	GH1	4.535318878	1
Thermoanaerobacter italicus	GH1	3.176195199	1
Escherichia coli	GH2	4.951896641	1
Burkholderia sp.	GH2	3.185120973	1
Opitutus terrae	GH2	2.849000161	1
Dictyoglomus turgidum	GH1	2.698198103	1
Solibacter usitatus	GH1	3.694382176	1
Dictyoglomus thermophilum	GH1	2.597989089	1
Lactobacillus amylovorus	GH1	4.651593108	1
Dickeya zeae	GH1	3.05858332	1
Klebsiella pneumoniae	GH1	3.527499461	1
Cyanothece sp.	GH1	2.72078854	1
Porphyromonas gingivalis	GH2	4.203215583	1
Bifidobacterium dentium	GH1	4.766725591	1
Shigella flexneri	GH2	4.384218202	1
Clostridium botulinum	GH30	4.053974807	1
Clostridium saccharolyticum	GH2	5.056490176	1
Clavibacter michiganensis	GH1	3.019422612	1
Streptococcus uberis	GH1	4.209153639	1
Atlantibacter hermannii	GH2	0.680539219	0
Gordonia bronchialis	GH1	2.602291518	1
Clostridium botulinum	GH1	4.053974807	1
Xylanimonas cellulosilytica	GH1	3.169152732	1

Sphaerobacter thermophilus	GH1	2.269246833	1
Aliivibrio fischeri	GH1	2.148976013	1
Bacillus cytotoxicus	GH1	3.068512384	1
Streptococcus equi	GH1	3.491210821	1
Streptomyces griseus	GH30	2.688132045	1
Prevotella ruminicola	GH2	4.464744795	1
Clostridium botulinum	GH30	4.053974807	1
Streptococcus pneumoniae	GH2	3.898009613	1
Fibrobacter succinogenes	GH30	3.403591123	1
Saccharomonospora viridis	GH2	2.402906227	1
Alistipes shahii	GH30	5.507004832	1
Rhodococcus erythropolis	GH2	4.017441033	1
Burkholderia thailandensis	GH2	3.076840585	1
Bifidobacterium dentium	GH2	4.766725591	1
Flavobacterium johnsoniae	GH2	3.525155341	1
Frankia inefficax	GH2	2.921941994	1
Roseburia intestinalis	GH2	5.494855325	1
Bacillus clausii	GH2	3.122214447	1
Prevotella ruminicola	GH2	4.464744795	1
Escherichia coli	GH2	4.951896641	1
Spirosoma linguale	GH1	3.535049207	1
Paenibacillus polymyxa	GH1	3.727193955	1
Actinobacillus pleuropneumoniae	GH2	2.854550453	1
Bacteroides vulgatus	GH2	5.981194327	1
[Eubacterium] siraeum	GH2	5.238059667	1
Caldicellulosiruptor obsidiansis	GH2	3.232298508	1
Geobacillus sp.	GH2	3.64830473	1
Brachyspira murdochii	GH1	2.974668271	1
Exiguobacterium sp.	GH1	3.604041401	1
Leptotrichia buccalis	GH1	3.234674622	1
Chitinophaga pinensis	GH30	3.132181861	1
Caldicellulosiruptor kronotskyensis	GH30	3.162669177	1
Enterobacter lignolyticus	GH1	3.968988727	1
Bacillus cereus	GH1	3.080867958	1
Escherichia coli	GH1	4.951896641	1
Gramella forsetii	GH2	3.311848451	1
Escherichia coli	GH2	4.951896641	1
Yersinia pseudotuberculosis	GH2	3.271820733	1
Pseudarthrobacter chlorophenolicus	GH1	2.741360004	1
Erwinia amylovora	GH2	2.90156558	1

Escherichia coli	GH2	4.951896641	1
Thermoanaerobacterium thermosaccharolyticum	GH2	3.608969791	1
Bacillus pumilus	GH1	3.248670682	1
Ruminococcus gnavus	GH1	5.39652351	1
Brevibacillus brevis	GH2	3.589469845	1
Sinorhizobium fredii	GH2	2.853225066	1
Geobacillus sp.	GH2	3.64830473	1
Clostridium cellulovorans	GH1	4.295967255	1
Kribbella flavida	GH1	2.574757512	1
Oenococcus oeni	GH1	3.610261575	1
Agrobacterium radiobacter	GH2	2.773077398	1
Shigella sonnei	GH2	4.50932433	1
Bifidobacterium adolescentis	GH30	5.563837622	1
Tolumonas auensis	GH1	3.248437991	1
Catenulispora acidiphila	GH2	2.886534124	1
uncultured bacterium	GH2	4.006358463	1
Lactobacillus acidophilus	GH1	4.391043616	1
Segniliparus rotundus	GH1	2.392276228	1
Rhizobium etli	GH2	2.801391781	1
Vibrio cholerae	GH1	2.401983537	1
Paenibacillus polymyxa	GH1	3.727193955	1
Geobacillus sp.	GH2	3.64830473	1
Roseiflexus castenholzii	GH1	1.70877002	1
Cutibacterium acnes	GH2	3.525541687	1
Listeria innocua	GH1	2.642937634	1
Xylanimonas cellulosilytica	GH2	3.169152732	1
Bifidobacterium adolescentis	GH2	5.563837622	1
Chitinophaga pinensis	GH2	3.132181861	1
Bacillus subtilis	GH1	3.020649231	1
Escherichia coli	GH1	4.951896641	1
Streptococcus pneumoniae	GH2	3.898009613	1
Staphylococcus saprophyticus	GH2	3.069986965	1
Clostridium botulinum	GH1	4.053974807	1
Escherichia coli	GH2	4.951896641	1
Cutibacterium acnes	GH2	3.525541687	1
Xanthomonas campestris	GH2	2.836321535	1
Bacillus megaterium	GH1	3.09272887	1
Shigella sonnei	GH2	4.50932433	1
Fervidobacterium nodosum	GH1	2.056375335	1
Dictyoglomus thermophilum	GH2	2.597989089	1

Escherichia coli	GH30	4.951896641	1
Bifidobacterium animalis	GH30	4.326031942	1
Solibacter usitatus	GH2	3.694382176	1
Clostridium perfringens	GH2	4.092439715	1
Truepera radiovictrix	GH1	2.750266906	1
Erwinia billingiae	GH1	3.247037685	1
Rhizobium meliloti	GH2	2.887519452	1
Escherichia coli	GH2	4.951896641	1
Catenulispora acidiphila	GH1	2.886534124	1
Escherichia coli	GH2	4.951896641	1
Arthrobacter sp.	GH1	2.788922725	1
Streptococcus pneumoniae	GH1	3.898009613	1
Frankia inefficax	GH1	2.921941994	1
Clostridium botulinum	GH30	4.053974807	1
Streptomyces ambofaciens	GH1	2.51396916	1
Shigella sonnei	GH1	4.50932433	1
Cronobacter sakazakii	GH1	3.663917102	1
Shigella dysenteriae	GH2	4.389565899	1
Shigella boydii	GH2	4.467400665	1
Exiguobacterium sp.	GH1	3.604041401	1
Micromonospora aurantiaca	GH2	2.656747513	1
Bacillus velezensis	GH1	3.007591831	1
Escherichia coli	GH2	4.951896641	1
Streptococcus pneumoniae	GH1	3.898009613	1
Leptospira biflexa	GH2	2.694610008	1
Azospirillum sp.	GH1	2.549653156	1
Clostridium cellulovorans	GH1	4.295967255	1
Bacteroides xylanisolvens	GH2	5.755848669	1
[Eubacterium] rectale	GH1	5.645131414	1
Porphyromonas gingivalis	GH2	4.203215583	1
Paenibacillus sp.	GH2	3.716301192	1
Maribacter sp.	GH2	3.442416285	1
Spirosoma linguale	GH1	3.535049207	1
Cellvibrio japonicus	GH2	2.097488885	0
Thermotoga petrophila	GH2	1.906618403	0
Streptomyces avermitilis	GH2	2.587827185	1
Shigella boydii	GH1	4.467400665	1
Escherichia coli	GH1	4.951896641	1
Escherichia coli	GH2	4.951896641	1
Chloroflexus aggregans	GH2	1.316259304	1

Pseudarthrobacter chlorophenolicus	GH1	2.741360004	1
Marinomonas sp.	GH1	3.075451459	1
Acidobacterium capsulatum	GH2	2.325925309	1
Thermobispora bispora	GH1	2.603002819	1
[Eubacterium] siraeum	GH2	5.238059667	1
Faecalitalea cylindroides	GH1	5.253809626	1
Bacteroides fragilis	GH2	5.330440553	1
Catenulispora acidiphila	GH30	2.886534124	1
Streptococcus thermophilus	GH2	3.238181275	1
Cupriavidus pinatubonensis	GH2	3.164662741	1
Myxococcus xanthus	GH1	3.04510265	1
Paenibacillus sp.	GH1	3.716301192	1
Bacteroides vulgatus	GH2	5.981194327	1
Alistipes shahii	GH2	5.507004832	1
Leuconostoc lactis	GH2	2.868933009	1
Beutenbergia cavernae	GH89	2.742712219	1
Halanaerobium praevalens	CBM32	3.109148225	1
Saccharophagus degradans	CBM32	2.105502422	0
Bacteroides fragilis	GH89	5.330440553	1
Caulobacter vibrioides	GH89	1.916277954	1
Paludibacter propionicigenes	GH89	3.910160743	1
Akkermansia muciniphila	GH89	5.317459779	1
Cellvibrio japonicus	CBM32	2.097488885	0
Bacteroides xylanisolvens	GH89	5.755848669	1
Acidobacterium capsulatum	GH89	2.325925309	1
Streptomyces bingchenggensis	CBM32	2.546261927	1
Caulobacter segnis	GH89	2.163370478	1
Clostridium perfringens	CBM32	4.092439715	1
Hahella chejuensis	CBM32	2.204844609	0
Caldicellulosiruptor saccharolyticus	CBM32	3.082502309	1
Clostridium perfringens	CBM32	4.092439715	1
Pedobacter heparinus	GH89	3.537943992	1
Chitinophaga pinensis	GH89	3.132181861	1
Bacteroides xylanisolvens	CBM32	5.755848669	1
Bacteroides thetaiotaomicron	CBM32	5.668359112	1
Roseburia intestinalis	CBM32	5.494855325	1
Thermobaculum terrenum	CBM32	2.7917651	1
Yersinia enterocolitica	CBM32	3.206616339	1
Xanthomonas axonopodis	GH89	1.902261106	1
Pedobacter heparinus	GH89	3.537943992	1

Bacteroides xylanisolvens	GH89	5.755848669	1
Bifidobacterium bifidum	CBM32	5.013833965	1
Zunongwangia profunda	GH89	3.493179318	1
Bacteroides xylanisolvens	GH89	5.755848669	1
Bifidobacterium bifidum	CBM32	5.013833965	1
Thermosipho africanus	CBM32	2.118718832	0
Stigmatella aurantiaca	CBM32	2.897849609	1
Saccharophagus degradans	CBM32	2.105502422	0
Stenotrophomonas maltophilia	CBM32	2.815564918	1
Bacteroides xylanisolvens	GH89	5.755848669	1
Akkermansia muciniphila	GH89	5.317459779	1
Herpetosiphon aurantiacus	CBM32	2.467219737	1
Bacteroides fragilis	CBM32	5.330440553	1
Xanthomonas campestris	CBM32	2.836321535	1
Pirellula staleyi	CBM32	1.362526726	0
Salinispora arenicola	CBM32	2.461696715	1
Dictyoglomus thermophilum	CBM32	2.597989089	1
Catenulispora acidiphila	CBM32	2.886534124	1
Bacteroides thetaiotaomicron	GH89	5.668359112	1
Streptomyces bingchenggensis	GH89	2.546261927	1
Kitasatospora setae	CBM32	2.897849609	1
Clostridium cellulovorans	CBM32	4.295967255	1
Bacteroides thetaiotaomicron	GH89	5.668359112	1
Kitasatospora setae	CBM32	2.897849609	1
Streptomyces bingchenggensis	GH89	2.546261927	1
Stenotrophomonas maltophilia	CBM32	2.815564918	1
Streptomyces ambofaciens	CBM32	2.51396916	1
Clostridium perfringens	CBM32	4.092439715	1
Clostridium perfringens	CBM32	4.092439715	1
Bacteroides fragilis	CBM32	5.330440553	1
Streptococcus suis	CBM32	4.75869111	1
Bacteroides fragilis	GH89	5.330440553	1
Prevotella ruminicola	GH89	4.464744795	1
Clostridium perfringens	GH89	4.092439715	1
Salinispora arenicola	CBM32	2.461696715	1
Anaerococcus prevotii	CBM32	3.578456843	1
Bifidobacterium bifidum	CBM32	5.013833965	1
Clostridium perfringens	CBM32	4.092439715	1
Paenibacillus sp.	CBM32	3.716301192	1
Streptomyces bingchenggensis	CBM32	2.546261927	1

Paenibacillus sp.	CBM32	3.716301192	1
Catenulispora acidiphila	CBM32	2.886534124	1
Herpetosiphon aurantiacus	CBM32	2.467219737	1
Paenibacillus sp.	CBM32	3.716301192	1
Clostridium perfringens	CBM32	4.092439715	1
Streptococcus mitis	CBM32	4.432157949	1
Catenulispora acidiphila	CBM32	2.886534124	1
Kitasatospora setae	CBM32	2.897849609	1
Bacteroides vulgatus	GH89	5.981194327	1
Brachybacterium faecium	GH89	2.322733424	1
Vibrio vulnificus	CBM32	1.625869887	0
Bacillus cereus	CBM32	3.080867958	1
Streptomyces scabiei	CBM32	2.583957419	1
Bifidobacterium bifidum	CBM32	5.013833965	1
Opitutus terrae	CBM32	2.849000161	1
Burkholderia lata	CBM32	2.633788296	1
Micromonospora aurantiaca	CBM32	2.656747513	1
Bacteroides thetaiotaomicron	CBM32	5.668359112	1
Stigmatella aurantiaca	CBM32	2.897849609	1
Bacteroides fragilis	CBM32	5.330440553	1
Bifidobacterium bifidum	GH89	5.013833965	1
Streptosporangium roseum	CBM32	2.74523594	1
Streptococcus mitis	CBM32	4.432157949	1
Herpetosiphon aurantiacus	CBM32	2.467219737	1
Streptomyces griseus	CBM32	2.688132045	1
Cutibacterium acnes	CBM32	3.525541687	1
Desulfofarcimen acetoxidans	CBM32	3.808845581	1
Geobacillus sp.	CBM32	3.64830473	1
Bacteroides vulgatus	CBM32	5.981194327	1
Stigmatella aurantiaca	CBM32	2.897849609	1
Lactococcus lactis	CBM32	4.535318878	1
Streptomyces bingchenggensis	CBM32	2.546261927	1
Kitasatospora setae	CBM32	2.897849609	1
Salinispora arenicola	CBM32	2.461696715	1
Lactobacillus plantarum	CBM32	3.483288678	1
Bacteroides xylanisolvens	CBM32	5.755848669	1
Streptomyces griseus	CBM32	2.688132045	1
Saccharophagus degradans	CBM32	2.105502422	0
Pectobacterium carotovorum	CBM32	3.146442611	1
Streptomyces ambofaciens	CBM32	2.51396916	1

Streptococcus equi	CBM32	3.491210821	1
Streptomyces bingchenggensis	CBM32	2.546261927	1
Colwellia psychrerythraea	CBM32	2.109088489	1
Catenulispora acidiphila	CBM32	2.886534124	1
Kitasatospora setae	CBM32	2.897849609	1
Kribbella flavida	CBM32	2.574757512	1
Herpetosiphon aurantiacus	CBM32	2.467219737	1
Stenotrophomonas maltophilia	CBM32	2.815564918	1
Streptosporangium roseum	CBM32	2.74523594	1
Streptococcus pneumoniae	CBM32	3.898009613	1
Zunongwangia profunda	CBM32	3.493179318	1
Arcanobacterium haemolyticum	CBM32	3.045776263	1
Geobacillus sp.	CBM32	3.64830473	1
Flavobacterium johnsoniae	CBM32	3.525155341	1
Streptomyces bingchenggensis	CBM32	2.546261927	1
Paenibacillus sp.	CBM32	3.716301192	1
Bacteroides fragilis	CBM32	5.330440553	1
Bacteroides thetaiotaomicron	CBM32	5.668359112	1
Bifidobacterium bifidum	CBM32	5.013833965	1
Nocardiopsis dassonvillei	CBM32	2.731716088	1
Streptococcus pneumoniae	CBM32	3.898009613	1
Clostridium perfringens	CBM32	4.092439715	1
Teredinibacter turnerae	CBM32	2.455699954	1
Paenibacillus sp.	CBM32	3.716301192	1
Streptococcus pneumoniae	CBM32	3.898009613	1
Kribbella flavida	CBM32	2.574757512	1
Cellvibrio japonicus	CBM32	2.097488885	0
Stigmatella aurantiaca	CBM32	2.897849609	1
Bacteroides thetaiotaomicron	CBM32	5.668359112	1
Streptococcus pneumoniae	CBM32	3.898009613	1
Methylococcus capsulatus	CBM32	2.920459839	1
Kitasatospora setae	CBM32	2.897849609	1
Paenibacillus sp.	CBM32	3.716301192	1
Kitasatospora setae	CBM32	2.897849609	1
Burkholderia glumae	CBM32	3.062451828	1
Stackebrandtia nassauensis	CBM32	2.705407885	1
Chitinophaga pinensis	CBM32	3.132181861	1
Desulfitobacterium hafniense	CBM32	3.849511971	1
Bifidobacterium longum	CBM32	5.049759625	1
Prevotella ruminicola	CBM32	4.464744795	1

Paenibacillus sp.	CBM32	3.716301192	1
Catenulispora acidiphila	CBM32	2.886534124	1
Bifidobacterium bifidum	CBM32	5.013833965	1
Microbacterium sp.	CBM32	0.993644905	0
Streptomyces bingchenggensis	CBM32	2.546261927	1
Catenulispora acidiphila	CBM32	2.886534124	1
Paenibacillus polymyxa	CBM32	3.727193955	1
Clostridium paraputrificum	CBM32	2.973291143	1
Catenulispora acidiphila	CBM32	2.886534124	1
Salmonella arizonae	CBM32	3.769206766	1
Streptosporangium roseum	CBM32	2.74523594	1
Streptococcus pneumoniae	CBM32	3.898009613	1
Caldicellulosiruptor kronotskyensis	CBM32	3.162669177	1
Catenulispora acidiphila	CBM32	2.886534124	1
Stigmatella aurantiaca	CBM32	2.897849609	1
Haliangium ochraceum	CBM32	2.906275029	1
Paenibacillus sp.	CBM32	3.716301192	1
Catenulispora acidiphila	CBM32	2.886534124	1
Saccharophagus degradans	CBM32	2.105502422	0
Chitinophaga pinensis	CBM32	3.132181861	1
Haliangium ochraceum	CBM32	2.906275029	1
Bacteroides fragilis	CBM32	5.330440553	1
Streptococcus suis	CBM32	4.75869111	1
Paenibacillus polymyxa	CBM32	3.727193955	1
Stigmatella aurantiaca	CBM32	2.897849609	1
Xylella fastidiosa	CBM32	2.313735638	1
Streptosporangium roseum	CBM32	2.74523594	1
Segniliparus rotundus	CBM32	2.392276228	1
Saccharophagus degradans	CBM32	2.105502422	0
Desulfitobacterium hafniense	CBM32	3.849511971	1
Streptococcus pneumoniae	CBM32	3.898009613	1
Salinispora tropica	CBM32	2.512074083	1
Streptosporangium roseum	CBM32	2.74523594	1
Bifidobacterium bifidum	CBM32	5.013833965	1
Desulfofarcimen acetoxidans	CBM32	3.808845581	1
Flavobacterium johnsoniae	GH89	3.525155341	1
Clostridium beijerinckii	CBM32	4.172192327	1
Streptomyces scabiei	GH89	2.583957419	1
Saccharophagus degradans	CBM32	2.105502422	0
Brevibacillus brevis	CBM32	3.589469845	1

Stigmatella aurantiaca	CBM32	2.897849609	1
Clostridium perfringens	CBM32	4.092439715	1
Catenulispora acidiphila	CBM32	2.886534124	1
Chitinophaga pinensis	CBM32	3.132181861	1
Cutibacterium acnes	CBM32	3.525541687	1
Catenulispora acidiphila	CBM32	2.886534124	1
Bacteroides fragilis	CBM32	5.330440553	1
Streptomyces ambofaciens	CBM32	2.51396916	1
Colwellia psychrerythraea	CBM32	2.109088489	1
Clostridium perfringens	CBM32	4.092439715	1
Paenibacillus sp.	CBM32	3.716301192	1
Streptomyces griseus	CBM32	2.688132045	1
Myxococcus xanthus	CBM32	3.04510265	1
Streptosporangium roseum	CBM32	2.74523594	1
Bacteroides thetaiotaomicron	CBM32	5.668359112	1
Chitinophaga pinensis	CBM32	3.132181861	1
Bacteroides vulgatus	CBM32	5.981194327	1
Caldicellulosiruptor kronotskyensis	CBM32	3.162669177	1
Catenulispora acidiphila	CBM32	2.886534124	1
Paenibacillus sp.	CBM32	3.716301192	1
Clostridium perfringens	CBM32	4.092439715	1
Bacillus cereus	CBM32	3.080867958	1
Kribbella flavida	CBM32	2.574757512	1
Bifidobacterium longum	CBM32	5.049759625	1
Paenibacillus sp.	CBM32	3.716301192	1
Kribbella flavida	CBM32	2.574757512	1
Paenibacillus polymyxa	CBM32	3.727193955	1
Paenibacillus sp.	CBM32	3.716301192	1
Bacteroides xylanisolvens	CBM32	5.755848669	1
Streptosporangium roseum	CBM32	2.74523594	1
Dickeya dadantii	CBM32	3.173053214	1
Catenulispora acidiphila	CBM32	2.886534124	1
Streptococcus pneumoniae	CBM32	3.898009613	1
Herpetosiphon aurantiacus	CBM32	2.467219737	1
Streptomyces bingchenggensis	CBM32	2.546261927	1
Caldicellulosiruptor kronotskyensis	CBM32	3.162669177	1
Streptomyces scabiei	CBM32	2.583957419	1
Kitasatospora setae	CBM32	2.897849609	1
Streptosporangium roseum	CBM32	2.74523594	1
Streptomyces bingchenggensis	CBM32	2.546261927	1

Burkholderia cenocepacia	CBM32	2.663298853	1
Micromonospora aurantiaca	CBM32	2.656747513	1
Lachnoclostridium phytofermentans	CBM32	4.440620629	1
Streptosporangium roseum	CBM32	2.74523594	1
Streptomyces griseus	CBM32	2.688132045	1
Pedobacter heparinus	CBM32	3.537943992	1
Akkermansia muciniphila	CBM32	5.317459779	1
Streptomyces ambofaciens	CBM32	2.51396916	1
Kribbella flavida	CBM32	2.574757512	1
Paludibacter propionicigenes	CBM32	3.910160743	1
Chitinophaga pinensis	CBM32	3.132181861	1
Saccharopolyspora erythraea	CBM32	2.829954331	1
Kitasatospora setae	CBM32	2.897849609	1
Caldicellulosiruptor owensensis	CBM32	3.105077595	1
Catenulispora acidiphila	CBM32	2.886534124	1
Catenulispora acidiphila	CBM32	2.886534124	1
Sanguibacter keddieii	CBM32	2.855701308	1
Streptosporangium roseum	CBM32	2.74523594	1
Hahella chejuensis	CBM32	2.204844609	0
Saccharophagus degradans	CBM32	2.105502422	0
Caldicellulosiruptor hydrothermalis	CBM32	3.083906066	1
Streptomyces scabiei	CBM32	2.583957419	1
Kribbella flavida	CBM32	2.574757512	1
Paenibacillus polymyxa	CBM32	3.727193955	1
Streptococcus pneumoniae	CBM32	3.898009613	1
Cellvibrio japonicus	CBM32	2.097488885	0
Myxococcus xanthus	CBM32	3.04510265	1
Bacteroides thetaiotaomicron	CBM32	5.668359112	1
Clostridium cellulovorans	CBM32	4.295967255	1
Streptomyces ambofaciens	GH89	2.51396916	1
Lachnoclostridium phytofermentans	CBM32	4.440620629	1
Lactobacillus johnsonii	CBM32	3.821988045	1
Lactococcus lactis	CBM32	4.535318878	1
Chitinophaga pinensis	CBM32	3.132181861	1
Sorangium cellulosum	CBM32	2.502583787	1
Clostridium perfringens	CBM32	4.092439715	1
Chitinophaga pinensis	CBM32	3.132181861	1
Catenulispora acidiphila	CBM32	2.886534124	1
Saccharophagus degradans	CBM32	2.105502422	0
Beutenbergia cavernae	CBM32	2.742712219	1

Haliangium ochraceum	CBM32	2.906275029	1
Haliangium ochraceum	CBM32	2.906275029	1
Caldicellulosiruptor kronotskyensis	CBM32	3.162669177	1
Mycoplasma crocodyli	CBM32	2.717915877	1
Kitasatospora setae	CBM32	2.897849609	1
Bacteroides fragilis	CBM32	5.330440553	1
Clostridium perfringens	CBM32	4.092439715	1
Stenotrophomonas maltophilia	CBM32	2.815564918	1
Bacteroides fragilis	CBM32	5.330440553	1
Bifidobacterium bifidum	CBM32	5.013833965	1
Xylella fastidiosa	CBM32	2.313735638	1
Brevundimonas subvibrioides	CBM32	2.525486274	1
Dyadobacter fermentans	CBM32	3.417983563	1
Catenulispora acidiphila	CBM32	2.886534124	1
Bifidobacterium bifidum	CBM32	5.013833965	1
Bacteroides thetaiotaomicron	CBM32	5.668359112	1
Paenibacillus sp.	CBM32	3.716301192	1
Bacteroides thetaiotaomicron	CBM32	5.668359112	1
Streptosporangium roseum	CBM32	2.74523594	1
Mycobacterium marinum	CBM32	2.21015987	0
Myxococcus xanthus	CBM32	3.04510265	1
Streptococcus pneumoniae	CBM32	3.898009613	1
Nocardiopsis dassonvillei	CBM32	2.731716088	1
Streptosporangium roseum	CBM32	2.74523594	1
Myxococcus fulvus	CBM32	3.087758264	1
Burkholderia ambifaria	CBM32	2.822519768	1
Caldicellulosiruptor kronotskyensis	CBM32	3.162669177	1
Bacteroides thetaiotaomicron	CBM32	5.668359112	1
Catenulispora acidiphila	CBM32	2.886534124	1
Spirochaeta thermophila	CBM32	3.412755316	1
Stackebrandtia nassauensis	GH89	2.705407885	1
Stigmatella aurantiaca	CBM32	2.897849609	1
Catenulispora acidiphila	CBM32	2.886534124	1
Lachnoclostridium phytofermentans	CBM32	4.440620629	1
Teredinibacter turnerae	CBM32	2.455699954	1
Ruminiclostridium cellulolyticum	CBM32	4.261509628	1
Streptococcus pneumoniae	CBM32	3.898009613	1
Pedobacter heparinus	GH89	3.537943992	1
Myxococcus xanthus	CBM32	3.04510265	1
Planctopirus limnophila	CBM32	0.835346957	0

Saccharophagus degradans	CBM32	2.105502422	0
Saccharophagus degradans	CBM32	2.105502422	0
Kribbella flavida	CBM32	2.574757512	1
Ralstonia solanacearum	CBM32	2.778990783	1
Bacteroides fragilis	CBM32	5.330440553	1
Chitinophaga pinensis	CBM32	3.132181861	1
Dictyoglomus turgidum	CBM32	2.698198103	1
Hungateiclostridium thermocellum	CBM32	4.161244363	1
Bacteroides fragilis	CBM32	5.330440553	1
Bacteroides vulgatus	CBM32	5.981194327	1
Rubrobacter xylanophilus	CBM32	2.512074083	1
Saccharophagus degradans	CBM32	2.105502422	0
Pseudoalteromonas atlantica	CBM32	2.341949533	1
Hahella chejuensis	CBM32	2.204844609	0
Myxococcus xanthus	CBM32	3.04510265	1
Bacillus halodurans	CBM32	3.382316202	1
Streptomyces ambofaciens	CBM32	2.51396916	1
Stigmatella aurantiaca	CBM32	2.897849609	1
Streptomyces griseus	CBM32	2.688132045	1
Clostridium perfringens	CBM32	4.092439715	1
Geobacillus sp.	CBM32	3.64830473	1
Hahella chejuensis	CBM32	2.204844609	0
Ralstonia pickettii	CBM32	3.121899211	1
Lachnoclostridium phytofermentans	CBM32	4.440620629	1
Kribbella flavida	CBM32	2.574757512	1
Geobacillus sp.	CBM32	3.64830473	1
Actinosynnema mirum	CBM32	2.698718791	1
Salinispora tropica	CBM32	2.512074083	1
Alistipes shahii	CBM32	5.507004832	1
Thermosipho melanesiensis	CBM32	1.894736198	0
Catenulispora acidiphila	CBM32	2.886534124	1
Bifidobacterium bifidum	CBM32	5.013833965	1
Bacteroides thetaiotaomicron	GH89	5.668359112	1
Stackebrandtia nassauensis	GH89	2.705407885	1
Catenulispora acidiphila	CBM32	2.886534124	1
Streptomyces griseus	CBM32	2.688132045	1
Clostridium perfringens	GH89	4.092439715	1
Streptomyces scabiei	CBM32	2.583957419	1
Catenulispora acidiphila	CBM32	2.886534124	1
Streptococcus equi	CBM32	3.491210821	1

Bifidobacterium bifidum	CBM32	5.013833965	1
Bifidobacterium longum	CBM32	5.049759625	1
Stigmatella aurantiaca	CBM32	2.897849609	1
Cellvibrio japonicus	CBM32	2.097488885	0
Streptococcus pneumoniae	CBM32	3.898009613	1
Spirochaeta thermophila	CBM32	3.412755316	1
Clostridium perfringens	CBM32	4.092439715	1
Paenibacillus sp.	CBM32	3.716301192	1
Bacteroides vulgatus	CBM32	5.981194327	1
Caldicellulosiruptor kronotskyensis	CBM32	3.162669177	1
Stigmatella aurantiaca	CBM32	2.897849609	1
Streptosporangium roseum	CBM32	2.74523594	1
Geobacillus sp.	CBM32	3.64830473	1
Salinispora arenicola	CBM32	2.461696715	1
Ruminiclostridium cellulolyticum	CBM32	4.261509628	1
Enterococcus faecalis	CBM32	4.493392116	1
Caldicellulosiruptor hydrothermalis	CBM32	3.083906066	1
Bacteroides thetaiotaomicron	CBM32	5.668359112	1
Atopobium parvulum	CBM32	3.852930421	1
Catenulispora acidiphila	CBM32	2.886534124	1
Streptomyces scabiei	CBM32	2.583957419	1
Streptosporangium roseum	CBM32	2.74523594	1
Stigmatella aurantiaca	CBM32	2.897849609	1
Stigmatella aurantiaca	CBM32	2.897849609	1
Streptomyces griseus	CBM32	2.688132045	1
Bacteroides fragilis	CBM32	5.330440553	1
Frankia alni	CBM32	2.650947694	1
Haliangium ochraceum	CBM32	2.906275029	1
Prevotella ruminicola	CBM32	4.464744795	1
Geobacillus sp.	CBM32	3.64830473	1
Catenulispora acidiphila	CBM32	2.886534124	1
Ruminiclostridium cellulolyticum	CBM32	4.261509628	1
Arcanobacterium haemolyticum	CBM32	3.045776263	1
Opitutus terrae	CBM32	2.849000161	1
Streptococcus pneumoniae	CBM32	3.898009613	1
Kitasatospora setae	CBM32	2.897849609	1
Saccharophagus degradans	CBM32	2.105502422	0
Alistipes shahii	GH89	5.507004832	1
Streptomyces bingchenggensis	CBM32	2.546261927	1
Saccharophagus degradans	CBM32	2.105502422	0

Streptosporangium roseum	CBM32	2.74523594	1
Streptosporangium roseum	CBM32	2.74523594	1
Geobacillus sp.	CBM32	3.64830473	1
Arcanobacterium haemolyticum	CBM32	3.045776263	1
Clostridium perfringens	CBM32	4.092439715	1
Kribbella flavida	CBM32	2.574757512	1
Catenulispora acidiphila	CBM32	2.886534124	1
Thermobispora bispora	CBM32	2.603002819	1
Kribbella flavida	CBM32	2.574757512	1
Bacteroides fragilis	CBM32	5.330440553	1
Bacteroides thetaiotaomicron	CBM32	5.668359112	1
Catenulispora acidiphila	CBM32	2.886534124	1
Streptococcus pneumoniae	CBM32	3.898009613	1
Saccharophagus degradans	CBM32	2.105502422	0
Streptococcus pneumoniae	CBM32	3.898009613	1
Streptomyces griseus	CBM32	2.688132045	1
Opitutus terrae	CBM32	2.849000161	1
Geobacillus sp.	CBM32	3.64830473	1
Salmonella arizonae	GH89	3.769206766	1
Caldicellulosiruptor obsidiansis	CBM32	3.232298508	1
Cutibacterium acnes	CBM32	3.525541687	1
Saccharophagus degradans	CBM32	2.105502422	0
Catenulispora acidiphila	CBM32	2.886534124	1
Bacteroides fragilis	GH89	5.330440553	1
Kribbella flavida	CBM32	2.574757512	1
Teredinibacter turnerae	CBM32	2.455699954	1
Streptococcus mitis	CBM32	4.432157949	1
Ralstonia pickettii	CBM32	3.121899211	1
Micromonospora aurantiaca	CBM32	2.656747513	1
Bacteroides thetaiotaomicron	CBM32	5.668359112	1
Cellvibrio japonicus	CBM32	2.097488885	0
Catenulispora acidiphila	CBM32	2.886534124	1
Salinispora arenicola	CBM32	2.461696715	1
Kribbella flavida	CBM32	2.574757512	1
Bifidobacterium bifidum	CBM32	5.013833965	1
Halanaerobium praevalens	CBM32	3.109148225	1
Chitinophaga pinensis	CBM32	3.132181861	1
Stenotrophomonas maltophilia	CBM32	2.815564918	1
Streptomyces griseus	CBM32	2.688132045	1
Heliobacterium modesticaldum	CBM32	3.825840835	1

Bacteroides fragilis	CBM32	5.330440553	1
Salinispora tropica	CBM32	2.512074083	1
Bifidobacterium longum	CBM32	5.049759625	1
Kribbella flavida	CBM32	2.574757512	1
Geobacter uraniireducens	CBM32	2.994140543	1
Akkermansia muciniphila	CBM32	5.317459779	1
Stigmatella aurantiaca	CBM32	2.897849609	1
Salinispora arenicola	CBM32	2.461696715	1
Streptomyces bingchenggensis	CBM32	2.546261927	1
Kitasatospora setae	CBM32	2.897849609	1
Chloroflexus aurantiacus	GH39	1.343763416	1
Roseiflexus castenholzii	GH39	1.70877002	1
Photorhabdus laumondii	GH39	2.248422138	1
Paludibacter propionicigenes	GH39	3.910160743	1
Fibrobacter succinogenes	GH39	3.403591123	1
Paraburkholderia phytofirmans	GH39	3.21698638	1
Arthrospira platensis	GH39	3.417854196	1
Caldicellulosiruptor bescii	GH39	3.125737391	1
Chloroflexus aurantiacus	GH39	1.343763416	1
Burkholderia pseudomallei	GH39	3.93219767	1
Clostridium botulinum	GH39	4.053974807	1
Dyadobacter fermentans	GH39	3.417983563	1
Chloroflexus aurantiacus	GH39	1.343763416	1
Opitutus terrae	GH39	2.849000161	1
Dictyoglomus turgidum	GH39	2.698198103	1
Roseiflexus sp.	GH39	2.092405838	1
Caldicellulosiruptor kronotskyensis	GH39	3.162669177	1
Cellulomonas flavigena	GH39	2.903929771	1
Xylanimonas cellulosilytica	GH39	3.169152732	1
Streptomyces bingchenggensis	GH39	2.546261927	1
Rhodococcus erythropolis	GH39	4.017441033	1
Burkholderia thailandensis	GH39	3.076840585	1
Geobacillus thermodenitrificans	GH39	3.274686288	1
Caldicellulosiruptor kronotskyensis	GH39	3.162669177	1
Roseiflexus castenholzii	GH39	1.70877002	1
Chloroflexus aurantiacus	GH39	1.343763416	1
Bacillus halodurans	GH39	3.382316202	1
Catenulispora acidiphila	GH39	2.886534124	1
Spirosoma linguale	GH39	3.535049207	1
[Ruminococcus] torques	GH39	5.495459591	1

Sphaerobacter thermophilus	GH39	2.269246833	1
Xanthomonas axonopodis	GH39	1.902261106	1
Caldicellulosiruptor bescii	GH39	3.125737391	1
Roseiflexus sp.	GH39	2.092405838	1
Cupriavidus taiwanensis	GH39	4.031877333	1
Flavobacterium johnsoniae	GH39	3.525155341	1
Conexibacter woesei	GH39	2.992179949	1
Pseudomonas mendocina	GH39	3.633057352	1
Roseiflexus sp.	GH39	2.092405838	1
Cellvibrio japonicus	GH39	2.097488885	0
Rhizobium leguminosarum	GH39	2.765664497	1
Streptomyces scabiei	GH39	2.583957419	1
Sphaerobacter thermophilus	GH39	2.269246833	1
Sphaerobacter thermophilus	GH39	2.269246833	1
Roseburia intestinalis	GH39	5.494855325	1
Burkholderia mallei	GH39	3.727548914	1
Roseiflexus sp.	GH39	2.092405838	1
Rhodopseudomonas palustris	GH39	2.986919212	1
Chloroflexus aggregans	GH39	1.316259304	1
Pseudomonas aeruginosa	GH39	3.269274895	1
gamma proteobacterium	GH39	3.904932646	1
Thermomicrobium roseum	GH39	2.028694511	0
Teredinibacter turnerae	GH39	2.455699954	1
Roseiflexus sp.	GH39	2.092405838	1
Roseiflexus sp.	GH39	2.092405838	1
Chloroflexus aggregans	GH39	1.316259304	1
Catenulispora acidiphila	GH39	2.886534124	1
Acidobacterium capsulatum	GH39	2.325925309	1
Paraburkholderia phymatum	GH39	0.762869044	0
Herpetosiphon aurantiacus	GH39	2.467219737	1
Streptomyces bingchenggensis	GH39	2.546261927	1
Bradyrhizobium sp.	GH39	2.919717144	1
Dictyoglomus thermophilum	GH39	2.597989089	1
Sanguibacter keddieii	GH39	2.855701308	1
Streptomyces scabiei	GH39	2.583957419	1
Geobacter bemidjiensis	GH39	2.993336071	1
Sphaerobacter thermophilus	GH39	2.269246833	1
Catenulispora acidiphila	GH39	2.886534124	1
Rhodococcus erythropolis	GH39	4.017441033	1
Xylanimonas cellulosilytica	GH39	3.169152732	1

Xanthobacter autotrophicus	GH39	3.677310993	1
Roseiflexus sp.	GH39	2.092405838	1
Photorhabdus laumondii	GH39	2.248422138	1
Klebsiella pneumoniae	GH39	3.527499461	1
Alkaliphilus oremlandii	GH39	4.685824844	1
Streptomyces bingchenggensis	GH39	2.546261927	1
Herpetosiphon aurantiacus	GH39	2.467219737	1
Xanthobacter autotrophicus	GH39	3.677310993	1
Actinosynnema mirum	GH39	2.698718791	1
Xanthobacter autotrophicus	GH39	3.677310993	1
Caldicellulosiruptor saccharolyticus	GH39	3.082502309	1
Thermomicrobium roseum	GH39	2.028694511	0
Roseiflexus castenholzii	GH39	1.70877002	1
Herpetosiphon aurantiacus	GH39	2.467219737	1
Bradyrhizobium sp.	GH39	2.919717144	1
Thermobispora bispora	GH39	2.603002819	1
Pseudomonas fluorescens	GH39	3.783203479	1
Serratia proteamaculans	GH39	3.231309256	1
Acidimicrobium ferrooxidans	GH39	2.557947421	1
Rhizobium leguminosarum	GH39	2.765664497	1
Sphaerobacter thermophilus	GH39	2.269246833	1
Thermotoga petrophila	GH39	1.906618403	0
Ruminiclostridium cellulolyticum	GH39	4.261509628	1
Pseudomonas stutzeri	GH39	3.504762609	1
Caldicellulosiruptor owensensis	GH39	3.105077595	1
Clostridium beijerinckii	GH39	4.172192327	1
Chitinophaga pinensis	GH39	3.132181861	1
Roseiflexus sp.	GH39	2.092405838	1
Roseiflexus castenholzii	GH39	1.70877002	1
Stackebrandtia nassauensis	GH39	2.705407885	1
gamma proteobacterium	GH39	3.904932646	1
Stackebrandtia nassauensis	GH39	2.705407885	1
Pseudomonas aeruginosa	GH39	3.269274895	1
Hungateiclostridium thermocellum	GH39	4.161244363	1
Chloroflexus aggregans	GH39	1.316259304	1
Xylanimonas cellulosilytica	GH39	3.169152732	1
Roseiflexus castenholzii	GH39	1.70877002	1
Chitinophaga pinensis	GH39	3.132181861	1
Spirosoma linguale	GH39	3.535049207	1
Bacillus megaterium	GH39	3.09272887	1

Chloroflexus aurantiacus	GH39	1.343763416	1
Burkholderia pseudomallei	GH39	3.93219767	1
Roseburia intestinalis	GH39	5.494855325	1
Catenulispora acidiphila	GH39	2.886534124	1
Kribbella flavida	GH39	2.574757512	1
Thermoanaerobacterium thermosaccharolyticum	GH39	3.608969791	1
Paraburkholderia xenovorans	GH39	3.175892682	1
Micromonospora aurantiaca	GH39	2.656747513	1
Chloroflexus aggregans	GH39	1.316259304	1
Burkholderia pseudomallei	GH39	3.93219767	1
Paenibacillus sp.	GH39	3.716301192	1
Pseudomonas savastanoi	GH39	3.06349684	1
Kineococcus radiotolerans	GH39	3.03645742	1
Roseiflexus sp.	GH39	2.092405838	1
Cellulomonas flavigena	GH39	2.903929771	1
Acidobacterium capsulatum	GH39	2.325925309	1
Roseiflexus castenholzii	GH39	1.70877002	1
Chloroflexus aggregans	GH39	1.316259304	1
Burkholderia mallei	GH39	3.727548914	1
Herpetosiphon aurantiacus	GH39	2.467219737	1
Roseiflexus sp.	GH39	2.092405838	1
Streptomyces scabiei	GH39	2.583957419	1
Caldicellulosiruptor owensensis	GH39	3.105077595	1
Burkholderia cenocepacia	GH39	2.663298853	1
Paenibacillus sp.	GH39	3.716301192	1
Burkholderia pseudomallei	GH39	3.93219767	1
Geobacter daltonii	GH39	3.509852462	1
Butyrivibrio proteoclasticus	GH39	4.213375923	1
Beijerinckia indica	GH39	3.78413834	1
Burkholderia pseudomallei	GH39	3.93219767	1
Bradyrhizobium diazoefficiens	GH39	3.166834227	1
Jonesia denitrificans	GH39	2.93633819	1
Chloroflexus aggregans	GH39	1.316259304	1
Klebsiella pneumoniae	GH39	3.527499461	1
Burkholderia mallei	GH39	3.727548914	1
Tsukamurella paurometabola	GH39	2.358358956	1
Herpetosiphon aurantiacus	GH39	2.467219737	1
Streptomyces bingchenggensis	GH39	2.546261927	1
Rhodopseudomonas palustris	GH39	2.986919212	1
Roseiflexus castenholzii	GH39	1.70877002	1

Caulobacter sp.	GH39	2.573978441	1
Xanthomonas campestris	GH39	2.836321535	1
Acaryochloris marina	GH39	2.383863923	1
Oceanithermus profundus	GH39	3.563551428	1
Brachybacterium faecium	GH39	2.322733424	1
Brevundimonas subvibrioides	GH39	2.525486274	1
Roseiflexus castenholzii	GH39	1.70877002	1
Thermoanaerobacterium thermosaccharolyticum	GH39	3.608969791	1
Methylacidiphilum infernorum	GH39	1.894736198	0
Herpetosiphon aurantiacus	GH39	2.467219737	1
Caldicellulosiruptor saccharolyticus	GH39	3.082502309	1
Roseiflexus castenholzii	GH39	1.70877002	1
Teredinibacter turnerae	GH39	2.455699954	1
Nitrospira defluvii	GH39	3.077741322	1
Kribbella flavida	GH39	2.574757512	1
Acidobacterium capsulatum	GH39	2.325925309	1
Pseudomonas mendocina	GH39	3.633057352	1
Catenulispora acidiphila	GH39	2.886534124	1
Roseiflexus castenholzii	GH39	1.70877002	1
Burkholderia pseudomallei	GH39	3.93219767	1
Solibacter usitatus	GH39	3.694382176	1
Beutenbergia cavernae	GH39	2.742712219	1
Clostridium acetobutylicum	GH39	1.970128234	0
Burkholderia pseudomallei	GH39	3.93219767	1
Pseudomonas syringae	GH39	3.439634781	1
Roseburia intestinalis	GH39	5.494855325	1
Pseudomonas fluorescens	GH39	3.783203479	1
Caldicellulosiruptor kronotskyensis	GH39	3.162669177	1
Conexibacter woesei	GH39	2.992179949	1
Chloroflexus aggregans	GH39	1.316259304	1
Thermobispora bispora	GH39	2.603002819	1
Caulobacter vibrioides	GH39	1.916277954	1
Roseiflexus sp.	GH39	2.092405838	1
Chloroflexus aurantiacus	GH39	1.343763416	1
Beutenbergia cavernae	GH39	2.742712219	1
Pedobacter heparinus	GH39	3.537943992	1
Conexibacter woesei	GH39	2.992179949	1
Pseudomonas syringae	GH39	3.439634781	1
Thermomicrobium roseum	GH39	2.028694511	0
Chloroflexus aurantiacus	GH39	1.343763416	1

Pseudomonas syringae	GH39	3.439634781	1
Catenulispora acidiphila	GH39	2.886534124	1
Kribbella flavida	GH39	2.574757512	1
Burkholderia thailandensis	GH39	3.076840585	1
Roseiflexus castenholzii	GH39	1.70877002	1
Clostridium cellulovorans	GH39	4.295967255	1
